# Techniques for Transvenous Lead Extraction of Cardiac Implantable Electronic Devices: A Network Meta‐Analysis

**DOI:** 10.1111/pace.70052

**Published:** 2025-09-26

**Authors:** Charles Karel Martins Santos, Maria Clara Ramos Miranda, Gabriel Alves Barbosa, Antônio da Silva Menezes Júnior

**Affiliations:** ^1^ Medical Department School of Medical and Life Sciences Pontifical Catholic University of Goiás Goiânia Goiás Brazil; ^2^ Medical Department Medical Faculty Federal University of Goiás Goiânia Goiás Brazil

**Keywords:** cardiac implantable electronic device, femoral approach, laser sheaths, rotating mechanical sheaths, transvenous lead extraction

## Abstract

**Background:**

Transvenous lead extraction (TLE) is procedurally complex and carries significant risk. Evidence on optimal TLE techniques is limited and lacks comparative studies.

**Methods:**

PubMed, Embase, Cochrane Library, and Web of Science were searched through November 27, 2024. We included randomized clinical trials (RCTs) or non‐randomized controlled trials (non‐RCTs) comparing two or more TLE methods in adults undergoing lead extraction. A network meta‐analysis was conducted to estimate pooled outcomes with 95% CIs. P‐scores ranked treatments.

**Results:**

Eleven non‐RCTs and one RCT were included. No statistically significant differences were observed in patient‐level clinical success or lead‐level procedural success. The femoral approach was associated with a significantly lower risk of significant complications compared to the use of laser sheaths (odds ratio, 0.28; 95% CI, 0.09–0.89). Rotating mechanical sheaths (RMS) ranked highest for clinical (*p* = 0.7470) and procedural success (*p* = 0.7357), while the femoral approach ranked highest for safety (*p* = 0.8368). Laser sheaths ranked lowest across all outcomes.

**Conclusion:**

No single technique was superior in terms of success rates. RMS and the femoral approach had the highest rankings for efficacy and safety, respectively. Laser sheaths ranked lowest for both. Rigorous prospective studies with direct comparative analyses are required to establish evidence‐based protocols and improve TLE patient outcomes.

## Graphical abstract

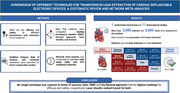

**Abbreviations**
CI: confidence Interval
CIED: cardiac implantable electronic device
ICD: implantable cardioverter‐defibrillator
MD: mean difference
NMA: network meta‐analysis
RCT: randomized clinical trial
RMS: rotating mechanical sheaths
RoB 2: cochrane risk of bias tool for randomized trials
SVC: superior vena cava
TLE: transvenous lead extraction

## 1 Introduction

With the development of heart failure and arrhythmia therapies, cardiac implantable electronic devices (CIEDs) have become essential in disease management and implantation rates continue to rise [1, 2]. This increasing dependence has led to an increased requirement for transvenous lead extraction (TLE), primarily due to device‐related infections and hardware malfunctions [3, 4, 5]. Advances in cardiac pacing and defibrillation devices has expanded the population of patients receiving treatment. Despite technological and procedural innovations that have markedly increased the clinical success and long‐term survival, device‐related complications remain a significant issue [6].

TLE poses essential challenge due to fibrotic tissue formation at the lead‐vascular interface, which can progressively undergo calcification and anchor the leads to adjacent vasculature, other leads, or the myocardium [7]. These complexities have spurred extensive research into optimal extraction techniques, including meta‐analyses. Nevertheless, previous meta‐analyses often grouped extraction methods into general categories instead of examining specific techniques, thereby limiting their clinical applicability. To address this gap and incorporate the latest evidence, we performed an updated meta‐analysis comparing multiple TLE methods for CIED extraction using both direct and indirect comparisons. Consequently, our approach facilitates a deeper evaluation of specific techniques and comprehensive taxonomies to enhance clinical decision making.

## 2 Methods

This systematic review and network meta‐analysis (NMA) followed the Preferred Reporting Items for Systematic Reviews and Meta‐Analyses guidelines [1] (Supplemental Methods 1 and 2). Our NMA methodology adhered to the Cochrane Handbook for Systematic Reviews of Interventions (Chapter 11) [2], ensuring rigorous, standardized evidence synthesis. The study protocol was registered with the International Prospective Register of Systematic Reviews under the registration number CRD42024612238.

### 2.1 Eligibility Criteria

We included studies that meeting the following criteria: (1) randomized clinical trials (RCTs) or non‐randomized observational studies; (2) adults aged ≥ 18 years undergoing TLE; (3) direct comparison of ≥ 2 predefined TLE strategies; and (4) sufficient patient‐ or lead‐level data for ≥ 1 pre‐specified primary or secondary endpoint. The treatment arms of interest included the following TLE strategies.

- Rotating mechanical sheaths (RMS): Powered mechanical extraction using a rotational dilator system.
- Laser sheaths: Laser‐assisted lead extraction.
- Femoral approach: Snare‐based extraction via femoral access.
- Traction: Simple manual traction (with or without locking stylets).

We excluded studies that met any of the following criteria: (1) < 10 patients per group, case series, case reports, or conference abstracts; (2) single‐arm studies or those without direct TLE strategy comparisons; (3) studies using non‐powered mechanical sheaths, primary surgical extraction, or electrosurgical dissection sheaths; (4) pediatric populations (<18 years) or congenital heart disease; (5) lack of patient‐ or lead‐level outcomes; and (6) duplicate or overlapping patient populations. In these cases, only the most comprehensive and extensive datasets were retained.

### 2.2 Search Strategy

We searched PubMed, Embase, the Cochrane Library, and Web of Science from inception to November 27, 2024, with no restrictions on language, publication date, or geographic region to ensure maximal sensitivity. The search strategy incorporated descriptors, controlled vocabulary terms (Medical Subject Headings in PubMed and Emtree terms in Embase), free‐text keywords, including synonyms and key term variations, and Boolean operators. To minimize retrieval bias, we manually screened the reference lists of included studies and relevant systematic reviews for additional eligible publications. The complete search syntax for each database is detailed in Supplemental Methods 3.

### 2.3 Study Selection and Data Extraction

Two reviewers independently screened studies and extracted data in duplicate. Discrepancies were resolved by consensus with a third reviewer. Records were uploaded to Rayyan, and a two‐step screening process was performed (title/abstract, then full‐text review) after deduplication. A standardized form was used to collect study characteristics, patient demographics, and outcomes. When available, adjusted data (e.g., propensity‐matched) were prioritized. All extracted data were independently cross‐verified for accuracy.

### 2.4 Endpoints

The primary endpoints comprised two efficacy measures and one safety endpoint. The primary safety endpoint was the incidence of major complications. The primary efficacy endpoints were as follows: (1) patient‐level clinical success and (2) lead‐level procedural success. Because patients could have multiple leads, efficacy was assessed separately at the lead and patient levels to account for clustering and prevent unit‐of‐analysis errors. Secondary safety endpoints included procedure duration and fluoroscopy time. All endpoints were assessed according to definitions specified in the included studies.

### 2.5 Statistical Analysis

The fundamental transitivity assumption (i.e., the assumption that the relative effect between two treatments can be inferred via one or more intermediate comparators) was evaluated by comparing the distribution of potential effect modifiers across different direct comparisons in the data [3, 4]. A frequentist NMA was conducted by comparing the competing treatment arms [5]. Given the anticipated heterogeneity in accurate effect sizes across studies, a random‐effects model was applied using the restricted maximum likelihood estimator for tau‐squared, to account for variability arising from diverse populations and underlying distributions. Binary endpoints were expressed as odds ratios (ORs), and continuous endpoints were reported as mean differences (MDs) with 95% confidence intervals (CIs). When means and standard deviations were not reported, these were imputed medians, or interquartile ranges, or other summary statistics [6, 7, 8].

Treatment rankings were evaluated using P‐scores, the frequentist equivalent of the Bayesian surface under the cumulative ranking (SUCRA) curve. P‐scores estimate the probability that a treatment is superior to competing alternatives, with higher scores indicating a better relative performance. However, P‐scores alone do not convey a complete distribution of ranking probabilities across all possible positions. To improve the interpretability of the treatment hierarchies, we used the rankogram function (from the netmeta package), which computes the probability of each treatment attaining every possible rank supplemented by SUCRA curve values. This approach provides a more comprehensive assessment of the ranking uncertainty. Probabilities were derived by resampling from a multivariate normal distribution, incorporating the estimated network effects and their variance‐covariance matrix [9].

Cochran's *Q* and *I* ^2^ statistics were used to assess heterogeneity and inconsistency. An *I* ^2^ < 25% indicated low heterogeneity, 25%–50% moderate, and > 50% high [10]. *Q* was further decomposed into within‐design and between‐design components to evaluate heterogeneity among and design inconsistency, respectively. Additional assessments of inconsistency for primary endpoints included (1) *Q* statistic estimation using a complete design‐by‐treatment interaction random‐effects model [11], (2) visual comparisons between direct and indirect estimates via net splitting [12, 13], and (3) generation of net heat plots to identify study inconsistency contributions [14]. Statistical significance was defined as *p* < 0.05. All analyses were performed using the netmeta [15] and dmetar [16] packages in R, version 4.2.2 (R Foundation for Statistical Computing).

### 2.6 Sensitivity Analysis

Sensitivity analyses were performed to assess network transitivity and further explore residual heterogeneity and inconsistency in the primary analyses. Pre‐specified sensitivity analyses included (1) exclusion of studies with high or critical risk of bias, which could artificially influence effect estimates; (2) exclusion of studies from low‐volume centers (< 100 enrolled patients), given that low procedural volume may be associated with greater variability due to limited technical expertise and less standardized protocols; and (3) exclusion of devices implanted for < 12 months to minimize confounding from short‐term effects.

### 2.7 Quality Assessment

Two authors independently assessed risk of bias, resolving any discrepancies by consensus. The quality of randomized studies was evaluated independently using the Cochrane risk‐of‐bias tool for randomized trials (RoB 2) [17], which assess five domains: random sequence generation, allocation concealment, performance bias, detection bias, and attribution bias. Observational studies were evaluated using the Risk of Bias Tool for Non‐Randomized Studies of Interventions (ROBINS‐I) [18], which considers potential biases across pre‐intervention, intervention, and post‐intervention domains. Publication bias was evaluated using comparison‐adjusted funnel plots (visual inspection) for primary endpoints and Egger's regression test for endpoints with ≥ 10 studies [19, 20].

## 3 Results

### 3.1 Study Selection and Baseline Characteristics

**FIGURE 1:**
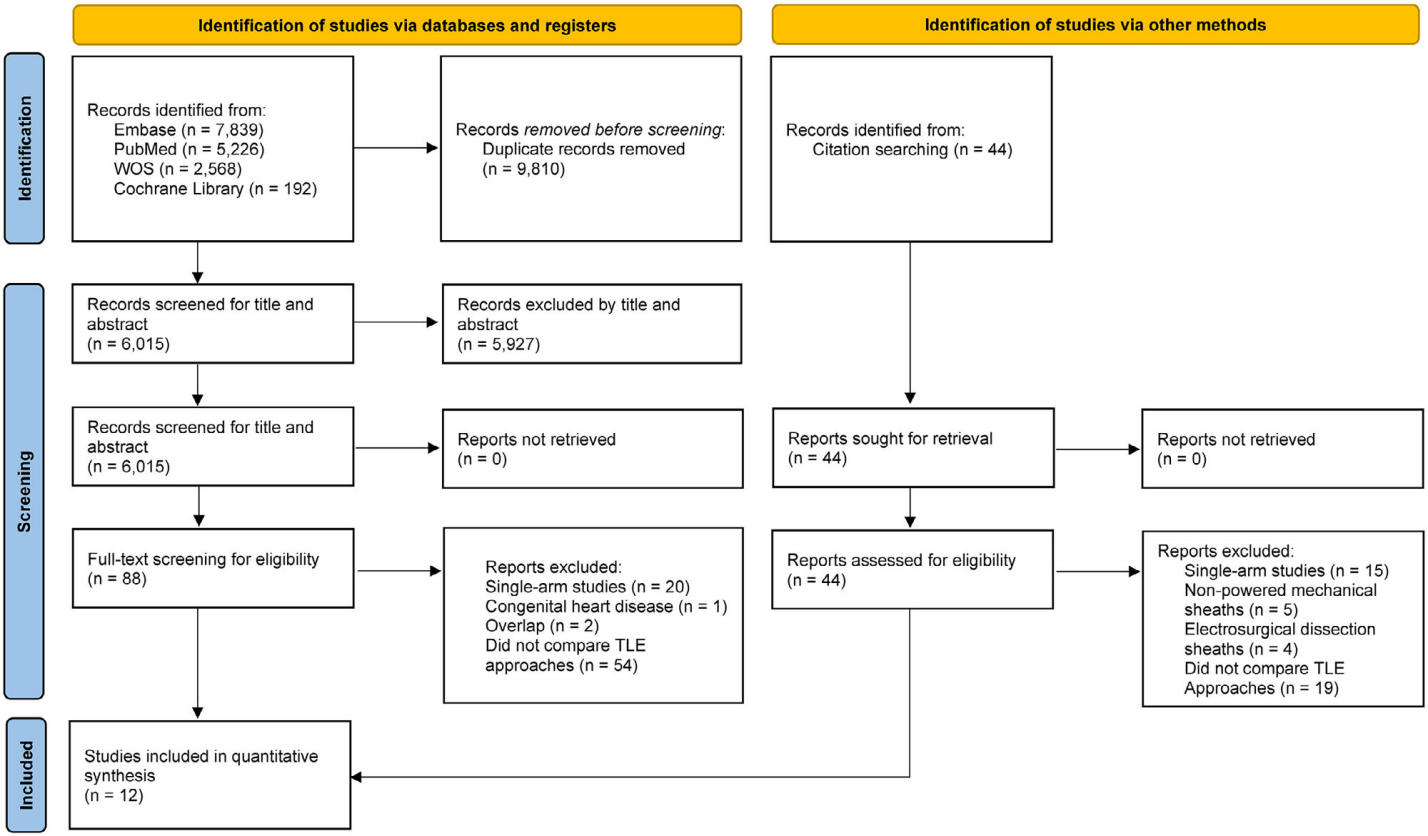
PRISMA flow diagram of study screening and selection. PRISMA flowchart illustrating the two‐step conventional double‐screening process for systematic reviews. PRISMA, Preferred Reporting Items for Systematic Reviews and Meta‐Analyses; RCT, randomized clinical trial; TLE, transvenous lead extraction; WOS, Web of Science. [Colour figure can be viewed at wileyonlinelibrary.com]

In total, 15,825 articles were initially retrieved, of which 6015 were screened after duplicate removal. Following title and abstract review, 5927 studies were excluded, leaving 88 articles for full‐text assessment. Among these, 20 were single‐arm studies, one included patients with congenital heart disease, and two had overlapping patient populations due to enrollment at the same center during concurrent periods. Additionally, 53 studies lacked comparative data on TLE tools. Ultimately, 12 studies [17, 18, 19, 20, 21, 22, 23, 24, 25, 26, 27, 28] met the inclusion criteria and were included in the final quantitative analysis (Figure 1).

Of the included studies, 11 were single‐center non‐RCTs, and one was a single‐center randomized trial. Sample sizes varied widely, ranging from 33 patients (60 leads) to 775 patients (1115 leads). Two studies compared the femoral approach with manual traction, one compared the femoral approach with RMS, five compared laser sheaths with RMS, one compared manual traction with RMS, and two compared the femoral approach with laser sheaths. One multi‐arm study simultaneously analyzed the femoral approach, laser sheaths, and RMS. The mean patient age ranged from 58.1 to 74 years, with male predominance (45.7%–82.2%). Lead dwell time ranged from 69.6 months to over 10 years. Infection was the primary indication for lead extraction in eight studies, reported in 36.5%–97.5% of cases (Table 1).

**TABLE 1:** Baseline characteristics of included studies.

Study, year	Design	Sample (*N*)	Intervention groups	Age (yr)^a^	Male sex (%)	Lead dwelling time^b^	Indication (%)^c^	
Uslu et al.2021 [21]	non‐RCT	94 pts137 leads	FA (*n* = 60 pts, 83 leads) T (*n* = 34 pts, 54 leads)	59.5 ± 16.8	70.2	FA: 87.5 ± 37.9 mo T: 31.3 ± 25.8 mo		Infection: 75.5 Non‐infection: 24.5
Jo et al.2016 [22]	non‐RCT	33 pts 60 leads	FA(*n* = 23 pts, 43 leads) T (*n* = 10 pts, 17 leads)	58.1 ± 14.1	69.7	106 (57–152) mo		Infection: 48.5 Non‐infection: 51.5
Starck et al. 2013 [13]	non‐RCT	138 leads	LS (*n* = 39 leads) RMS (*n* = 99 leads)	60.4 (23–89)	71.3	69.6 (1–384) mo		Infection: 41.3 Non‐infection: 58.7
Bordachar et al. 2010 [24]	RCT	101 pts	FA *n* = 51 pts) LS (*n* = 50 pts)	LS: 68.1 ± 14.34 FA: 67.0 ± 13.0	77.2	LS: 12 ± 6 yr FA: 13 ± 6 yr		Infection: 89.1 Non‐infection: 10.9
Kong et al. 2015 [25]	non‐RCT	76 pts 134 leads	FA (*n* = 59 pts, 103 leads) RMS (*n* = 17 pts, 31 leads)	68.1 ± 14.34	65.8	RMS: 10.8 ± 7.0 yr FA: 11.2 ± 8.4 yr		Infection: 97.4 Non‐infection: 2.6
Zsigmond et al. 2023 [26]	non‐RCT	142 pts 289 leads	LS (*n* = 93 pts, 159 leads) RMS (*n* = 49 pts, 86 leads)	65.4 ± 13.7	78	9.4 ± 6.3 yr		Infection: 94.4 Non‐infection: 5.6
Misra et al. 2021 [28]	non‐RCT	575 leads	LS (*n* = 395 leads) RMS (*n* = 180 leads)	RMS: 61.9 ± 16.1 LS: 63.9 ± 15.0	66.1	RMS: > 10 yr: 52.8% LS: > 10 yr: 28.4%		Infection: 36.5 Non‐infection: 63.5
Qin et al. 2021 [27]	non‐RCT	179 pts 342 leads	LS (*n* = 157 pts, 297 leads) RMS (*n* = 22 pts, 45 leads)	RMS: 66.9 ± 10.6 LS: 65.1 ± 14.3	67	RMS: 10.0 (2–25) yr LS: 8.2 (1–28) yr		Infection: 45.8 Non‐infection: 54.2
Lensvelt et al. 2021 [17]	non‐RCT	45 pts 95 leads	T (*n* = 10 pts, 24 leads) RMS (*n* = 35 pts, 71 leads)	RMS 71 (65–79) T: 74 (64–79)	82.2	RMS: 108 (86–155) mo T: 80 (72–98) mo		Infection: 86.7 Non‐infection: 13.3
Bracke et al. 2022 [18]	non‐RCT	775 pts 1115 leads	LS (*n* = 184 pts, 190 leads) FA (*n* = 321 pts, 717 leads) RMS (*n* = 270 pts, 208 leads)	70.3 (61–77.2)	74.5	RMS: 9.6 (6.5–14.7) yr LS: 8.1 (4.4–12.0) yr FA: 7.6 (4.5–11.2) yr		Infection: 89.4 Non‐infection: 10.6
Mazzone et al. 2013 [19]	non‐RCT	121 pts 208 leads	LS (*n* = 73 pts, 127 leads) RMS (*n* = 48 pts, 81 leads)	62.3 ± 14.4	45.7	77.5 ± 55 mo		Infection: 76.8 Non‐infection: 23.2
Zhou et al. 2021 [20]	non‐RCT	746 pts	FA (*n* = 692 pts) LS (*n* = 54 pts)	NI	71	NI		Infection: 88.9 Non‐infection: 11.1

*Note*: Summary of the design and demographic, clinical, and device‐related characteristics of the included studies.

Abbreviations: RCT, randomized clinical trial; FA, femoral approach; LS, laser sheaths; RMS, rotating mechanical sheaths; T, traction; SD, standard deviation; IQR, interquartile range; mo, month; yr, year; pt, patients; NI, not informed.

^a^ Age presented as mean ± SD or median (IQR).

^b^ Lead dwell time presented as mean ± SD, median (IQR), or % (mo/yr).

^c^ Primary indication for lead extraction.

Definitions and criteria adopted across studies are presented in Table S1. The studies encompassed a broad temporal range, with the earliest patient enrollment beginning in May 1997 and the most recent follow‐up ending in February 2021. Geographically, the studies included patients from Europe, Asia, and the Americas, with contributions from the United States, China, Netherlands, France, Italy, South Korea, and Hungary (Table S2). Clinical and demographic characteristics are summarized in Table S3. Device types varied across studies, with pacemakers being most frequently extracted, followed by implantable cardioverter‐defibrillators (ICDs) and cardiac resynchronization therapy devices. Most leads were located in the right ventricle, followed by the right atrium and coronary sinus (Table S4).

### 3.2 Primary Safety Endpoints

#### 3.2.1 Major Complications

**FIGURE 2:**
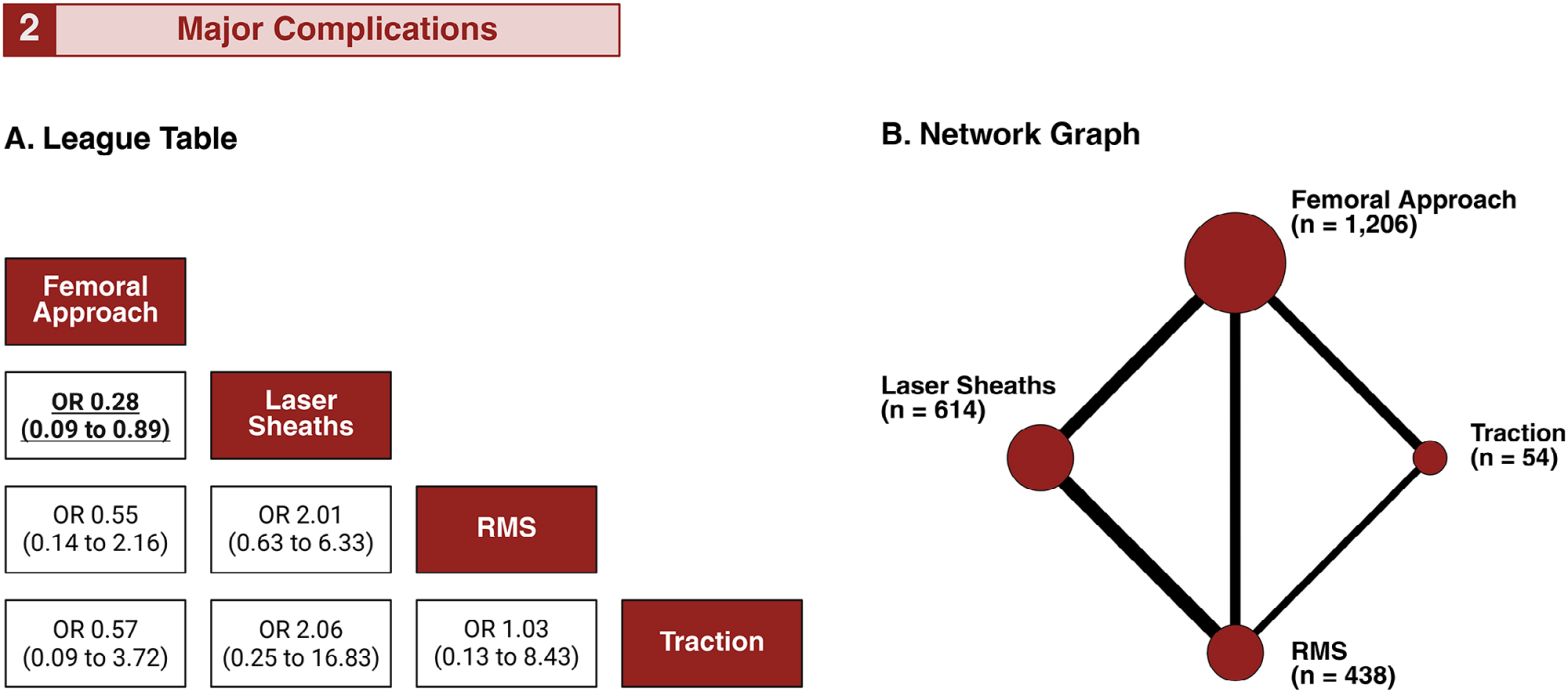
Network of treatment arms for primary safety endpoint. Network meta‐analysis of major complications. (A) League tables are displayed where the reported estimates (OR with 95% CI) represent column‐defining treatments compared to row‐defining treatments.(B) Net graphs are presented where nodes represent competing arms and edges indicate direct evidence between connected treatments. The node size was proportional to the total number of patients receiving each treatment across trials, and the edge thickness corresponded to the number of studies directly comparing connected strategies. CI, confidence interval; OR, odds ratio; RMS, rotating mechanical sheath. [Colour figure can be viewed at wileyonlinelibrary.com]

Figure 2 summarizes the network comparisons across 4 treatment strategies, spanning 6 distinct study designs, and 12 pairwise comparisons. Ten studies reported patient‐level major complications [17, 18, 19, 20, 21, 22, 24, 25, 26, 27], involving 2312 patients: 1206 in the femoral snare arm, 614 in the laser arm, 438 in the RMS arm, and 54 in the traction arm (Figure 2B). The femoral approach was associated with a significantly lower risk of significant complications than laser sheaths (OR 0.28; 95% CI 0.09–0.89) (Figure 2A). Forest plots for pairwise comparisons are provided in Supplemental Results, Section 1.

### 3.3 Primary Efficacy Endpoints

#### 3.3.1 Clinical Success

**FIGURE 3:**
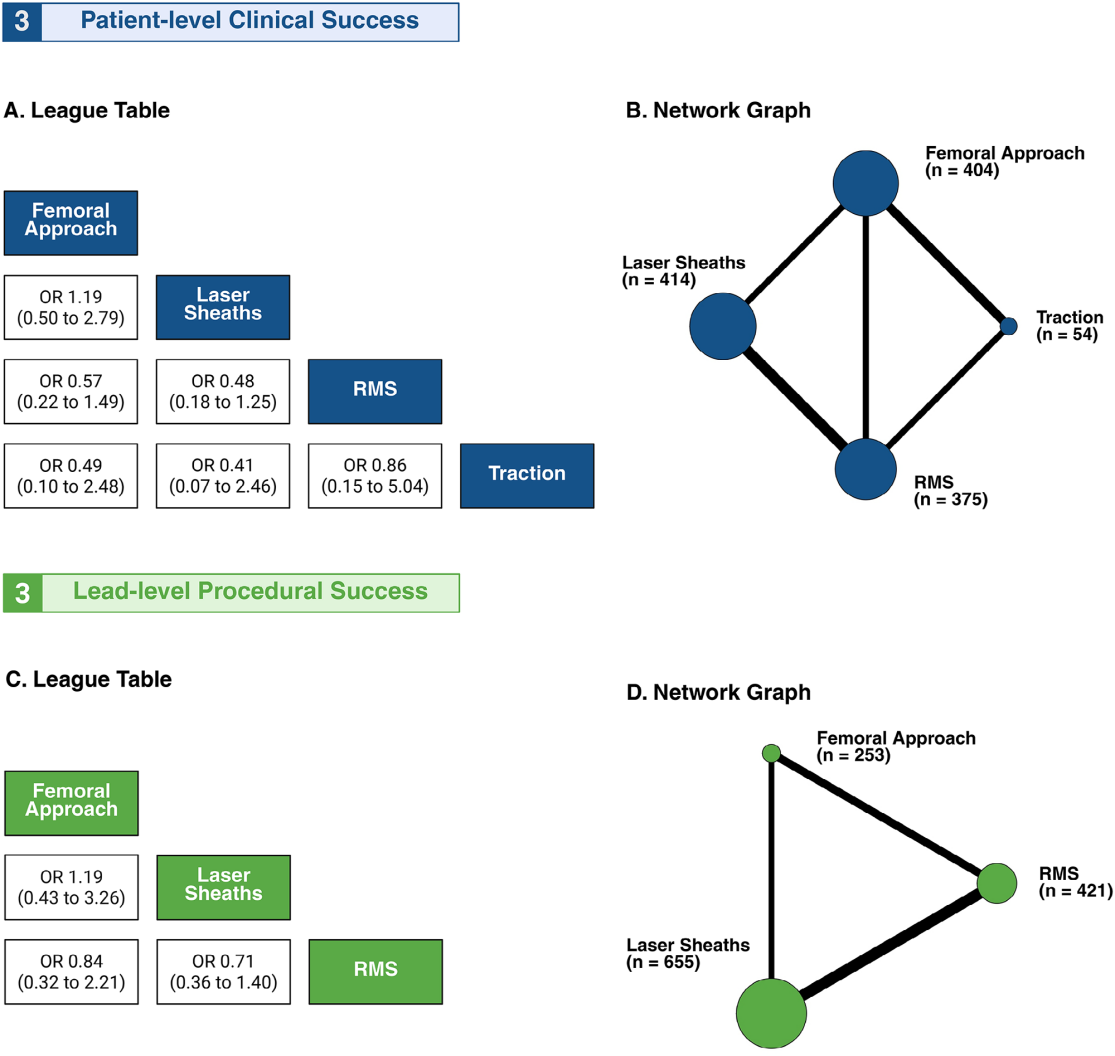
Network of treatment arms for primary efficacy endpoint. Network meta‐analysis of patient‐level clinical success (A, B) and lead‐level procedural success (C, D). A and C display league tables, where the reported estimates (OR with 95% CI) represent column‐defining treatments compared to row‐defining treatments. B and D present net graphs, where nodes represent competing arms and edges indicate direct evidence between the connected treatments. The node size was proportional to the total number of patients receiving each treatment across trials, and the edge thickness corresponded to the number of studies directly comparing connected strategies. CI, confidence interval; OR, odds ratio; RMS, rotating mechanical sheath. [Colour figure can be viewed at wileyonlinelibrary.com]

Figure 3 depicts the treatment network for the patient clinical success endpoint. The analysis comprised four treatment arms across four study designs and eight pairwise comparisons. Patient‐level clinical success was reported in six studies [17, 18, 19, 21, 22, 27] involving 1247 patients: 404 in the femoral snare arm, 414 in the laser arm, 375 in the RMS arm, and 54 in the traction arm (Figure 3B). No statistically significant differences were observed between treatment arms (Figure 3A). Forest plots for pairwise comparisons are provided in Supplemental Results, Section 2.

#### 3.3.2 Procedural Success

Figure 3 depicts the treatment network for lead‐level procedural success endpoint. The analysis comprised three treatment arms across three study designs and seven pairwise comparisons. Lead‐level procedural success was reported in five studies [18, 23, 25, 26, 27] involving 1,329 leads: 253 in the femoral snare arm, 655 in the laser arm, and 421 in the RMS arm (Figure 3D). No significant differences were observed between treatment arms (Figure 3C). Forest plots for pairwise comparisons are provided in Supplemental Results, Section 3.

### 3.4 Secondary Endpoints

#### 3.4.1 Procedure Time (minutes)

Supplemental Results 4 illustrate the treatment network for the procedure time analysis. Four treatment arms were assessed across three study designs and three pairwise comparisons. Procedure time was reported in three studies [17, 24, 25], totaling 222 observations: 110 in the femoral snare arm, 50 in the laser arm, 52 in the RMS arm, and 10 in the traction arm. The femoral approach required significantly longer procedure times compared with laser sheaths (MD 35 min; 95% CI 19.73–50.27), RMS (MD 31 min; 95% CI 15.00–47.00), and traction (MD 92.67 min; 95% CI 66.20–119.14; Supplemental Results 5). In contrast, traction alone demonstrated significantly shorter procedure times relative to both RMS (MD −61.67 min; 95% CI −82.76 to −40.58) and laser sheaths (MD −57.67 min; 95% CI −88.23 to −27.11; Supplemental Results 6).

#### 3.4.2 Fluoroscopy Time (minutes)

Supplemental Results 7 depict the network of treatment arms included in the fluoroscopy time analysis. Four treatment arms were evaluated across three study designs and three pairwise comparisons. Fluoroscopy time was reported in three studies [17, 24, 25], totaling 222 observations: 110 in the femoral snare arm, 50 in the laser arm, 52 in the RMS arm, and 10 in the traction arm. The femoral approach was associated with significantly prolonged fluoroscopy times versus laser sheaths (MD 14.00 min; 95% CI 8.95–19.05), RMS (MD 12.00 min; 95% CI 6.73–17.27), and traction (MD 15.33 min; 95% CI 9.29–21.37; Supplemental Results 8). Conversely, traction alone reduced fluoroscopy time significantly compared to RMS (MD −3.33 min; 95% CI −6.29 to −0.37) and the femoral approach (MD −15.33 min; 95% CI −21.37 to −9.29; Supplemental Results 9).

### 3.5 Ranking of TLE Strategies

**FIGURE 4:**
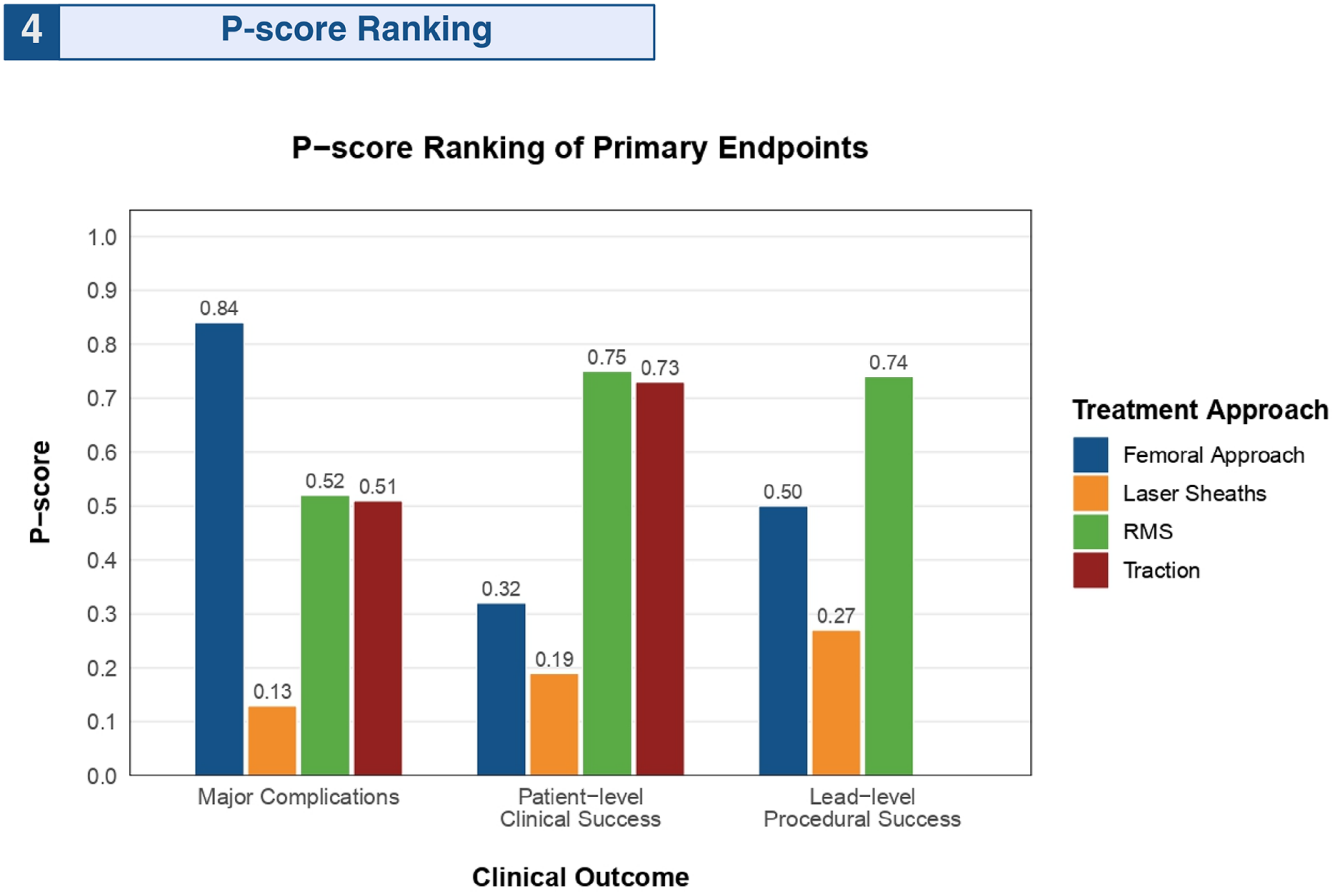
P‐score for treatment ranking in the network of primary safety and efficacy endpoints. Hierarchical ranking of treatment strategies based on p‐scores for major complications, clinical success, and procedural success. Higher p‐values indicated superior efficacy and safety. RMS, rotating mechanical sheath. [Colour figure can be viewed at wileyonlinelibrary.com]

Figure 4 displays the treatment arm rankings based on P‐score analysis. For safety outcomes, the femoral snare approach demonstrated the highest probability of minimizing major complications (P‐score = 0.8368), whereas laser sheaths showed the lowest probability (P‐score = 0.1276). For efficacy outcomes, RMS ranked highest for both patient‐level clinical success (P‐score = 0.7470) and procedural success (P‐score = 0.7357), while laser sheaths consistently ranked lowest in (clinical success, *p* = 0.1931; procedural success, *p* = 0.2664). Among secondary endpoints, the traction approach achieved the highest ranking for both procedure time reduction (P‐score = 1.0000) and fluoroscopy time minimization (P‐score = 0.8720). Conversely, the femoral approach ranked lowest for both temporal parameters (procedure time, *p* = 0; fluoroscopy time, *p* = 0). These findings were corroborated by SUCRA curve rankogram analyses, which showed strong concordance with the P‐score across all endpoints (Supplemental Results 10–12).

### 3.6 Sensitivity Analysis for Primary Endpoints

Three sequential sensitivity analyses were performed to evaluate the robustness of primary outcomes. First, exclusion of studies involving devices implanted for < 12 months revealed no statistically significant differences in major complications (four studies [18, 22, 24, 27], *n* = 1088 patients; Supplemental Results 13A), patient‐level clinical success (three studies [18, 22, 27], *n* = 987 patients; Supplemental Results 13B), or lead procedural success (three studies [18, 23, 27], *n* = 950 leads; Supplemental Results 13C). Second, after excluding small centers (< 100 patients), no significant outcome differences were observed in analyses of six studies [18, 19, 20, 24, 26, 27] (*n* = 2064 patients) for major complications, three studies [18, 19, 27] (*n* = 1075 patients) for clinical success, and four studies [18, 23, 26, 27] (*n* = 1195 leads) for procedural success (Supplemental Results 14). Finally, restricting the analysis to studies at low/moderate risk of bias yielded no significant outcome differences: four studies [17, 21, 24, 27] (*n* = 419 patients) for major complications, three studies [17, 21, 27] (*n* = 318 patients) for clinical success, and two studies [23, 27] (*n* = 480 leads) for procedural success (Supplemental Results 15).

#### 3.6.1 Inconsistency and Heterogeneity for Primary Endpoints

Moderate heterogeneity and significant between‐design inconsistencies were observed for major complications (*I* ^2^ = 29.5%; *p* for between‐design inconsistency = 0.7104; *p* for within‐design heterogeneity = 0.0415). The design‐by‐treatment interaction random‐effects model identified a source of inconsistency (Supplementary Results 17). However, separate direct and indirect evidence analyses revealed no significant differences (Supplemental Results 18–19). The net heat plot indicated that comparisons involving femoral approach and laser sheaths contributed to the observed inconsistency, as indicated by warmer‐colored cells and larger boxes (Supplemental Results 20).

No heterogeneity or inconsistency was observed for clinical success (*I* ^2^ = 0%; between‐design *p* = 0.5797; within‐design *p* = 0.9531). The design‐by‐treatment model confirmed consistency (Supplemental Results 17), and no discrepancies were observed between direct and indirect evidence (Supplemental Results 21–22). The net heat plot supported this finding, showing no concerning regions (Supplemental Results 23).

In contrast, substantial heterogeneity and inconsistency was observed for procedural success (*I* ^2^ = 60.3%), primarily driven by between‐design variability (*p* = 0.0646), rather than within‐design heterogeneity (*p* = 0.1005). A random‐effects model reduced inconsistency, suggesting improved model fit (Supplemental Results 17). No significant disagreement was noted between direct and indirect evidence (Supplemental Results 24–25). The net heat plot highlighted inconsistencies mainly in RMS versus LS comparisons, particularly in multiarm designs involving RMS, femoral approach, and laser sheaths; as illustrated by warmer‐colored cells and larger grey boxes at these intersections (Supplemental Results 26).

### 3.7 Quality Assessment

The ROBINS‐I assessment indicated moderate risk of bias in four studies [17, 21, 23, 27], and serious risk in six [18, 19, 20, 22, 25, 26]. According to the RoB 2 tool, one study [24] was classified as having moderate risk (Supplemental Results 27). In the major complication network, no evidence of publication bias was detected based on the comparison‐adjusted funnel plot and Egger test (*p* = 0.7197). Due to the limited number of studies, the Egger's test was not conducted for clinical and procedural success outcomes (Supplemental Results 28).

## 4 Discussion

This systematic review and NMA of 11 single‐center non‐RCTs and one randomized study compared the efficacy and safety of four TLE techniques: RMS, femoral approach, laser sheaths, and traction alone. The key findings were as follows: (1) The femoral approach significantly reduced major complications compared to laser sheaths in a network of 2312 patients, with an approximately threefold lower risk; (2) the femoral approach ranked highest for minimizing major complications among the four techniques; (3) RMS demonstrated superior performance in both patient‐level clinical success and lead‐level procedural success; (4) laser sheaths ranked lowest across patient‐level clinical success, lead‐level procedural success and major complications; and (5) fluoroscopy and procedure times were significantly longer with the femoral approach.

Direct comparative data on TLE techniques remain limited. Our safety results align with those of recently published studies. A 2021 meta‐analysis evaluated 14 studies on rotating sheaths and 34 studies on laser sheaths found laser sheaths to be associated with a 9.3‐fold increased risk of mortality [29]. However, this meta‐analysis predominantly included case series, introducing potential selection bias and high heterogeneity. Moreover, none of the studies directly compared procedural safety and powered sheaths, complicating interpretation. A 2023 meta‐analysis of 30 studies also aggregated non‐comparative data and reported a 2.2‐fold increased risk of procedural mortality with laser. Additionally, superior vena cava (SVC) injury occurred in 0.18% of RMS cases and 1.07% of laser cases, representing a 5.2‐fold increased risk [1]. Notably, not all studies included in this meta‐analysis directly compared laser and non‐laser techniques. Another 2023 proportion meta‐analysis reported a 1.9% rate of major complications with TLE using excimer laser sheaths, primarily consisting of SVC injury and procedure‐related death [30].

Laser‐assisted extraction has been investigated in multiple studies, with major complication rates ranging from 0.9% to 2.5% [31]. In the 1999 PLEXES trial (301 procedures), major complications occurred in three patients in the laser sheath group and none in the plastic sheath group [32]. In the LExICon study (*n* = 1449), a multicenter retrospective analysis, laser‐assisted extraction resulted in 4% of major adverse events, with 1.4% directly related to extraction [33]. Similarly, the GALLERY study—the largest laser lead extraction registry worldwide—reported a 4.32% overall complication rate among 2524 consecutive patients [31]. The ELECTRa study, involving 3510 patients who underwent TLE, reported a 1.7% rate of major procedure‐related complications [34]. Although ELECTRs was not powered to assess the comparative efficacy of different tools, subgroup analysis revealed a higher incidence of major cardiovascular complications—including vascular avulsion and cardiac avulsion or tear with tamponade—associated with laser sheaths (19.06%) compared to RMS (7.61%) [35]. However, a potential selection bias cannot excluded, as more complex cases may have been preferentially treated with powered sheaths.

One of the most catastrophic complications of transvenous pacemaker or ICD extraction is SVC perforation resulting from a tear, burn, or other injury, leading to uncontrolled hemorrhage into the right thorax [36]. Although rare, SVC laceration carries a high mortality rate (50%) and is considered the most severe complication [37]. The mechanism underlying SVC injury with laser sheaths remains unclear. One possible explanation is that laser sheaths emit tissue‐desiccating energy that affects areas beyond the sheath tip. Although the depth of laser penetration is generally shallow, repeated activation at fibrotic sites may cause thermal injury accumulation, leading to vascular damage [36, 38]. Another hypothesis is that laser sheaths induce explosive photothermal vaporization of cellular water, generating rapidly expanding bubbles. When these pressurized bubbles become trapped in front of the sheath, they may damage adjacent vascular structures [18, 37].

A 2019 retrospective study comparing mortality rates between rotating and laser sheaths during cardiac lead extraction reported 180 deaths (70%) over 6 years, of which 126 involved SVC injury. Rotating sheaths were implicated in 13 deaths, 92% attributed to cardiovascular injury, while laser sheaths were associated with 167 deaths, 95% cardiovascular in nature [38]. However, this analysis had several limitations. It lacked information on differences in patient characteristics, as the severity of conditions among patients using each sheath type could influence the observed outcomes. Furthermore, its retrospective nature introduced potential selection bias, including confounding by indication. Moreover, the concurrent use of both sheath types in some procedures complicated the attribution of mortality to a specific device.

Our NMA was underpowered to detect statistically significant differences in complication rates between RMS and laser sheaths. However, a significant difference was observed between laser sheaths and femoral approaches, with the laser sheaths associated with a 3.63‐fold increased risk of major complications. This outcome may be explained by the absence of upper thoracic vein manipulation in the femoral approach, reducing the likelihood of venous complications. In a 2021 randomized trial by Bordachar et al. [24], no significant differences were observed in procedural success or complication rates between the femoral and superior (laser sheaths) approaches. However, the femoral approach was associated with longer procedural and fluoroscopy times, consistent with our NMA results.

The efficacy and safety of RMS were analyzed in the PROMET study [30]. They reported a major complication rate of 1% and a procedure‐related mortality rate of 0.18%, suggesting that mechanical extraction tools are associated with low procedural risk. Notably, the nature of complications differed from those reported in laser‐based TLE, with a notably lower incidence of SVC injury in the PROMET cohort. Real‐world data also demonstrate comparable efficacy trends. In PROMET [30], mechanical TLE achieved clinical success in 97.0% of procedures, with complete extraction in 96.5% of leads. In contrast, the LEXICON study [33] reported a clinical success rate of 97.7% and procedural success of 98.8% with laser extraction. Similarly, the GALLERY study [31] demonstrated a 94.85% complete lead removal rate and 97.86% clinical success in laser‐based extraction. The PLEXES trial [32] reported a 94% complete removal rate in the laser group.

The combined use of different extraction tools is often required during TLE. Definitions of procedural and clinical success incorporate both technical success and safety‐related outcomes, which may be influenced by adverse tool‐related complications [39]. Although our study was underpowered for statistically significant pairwise comparisons, treatment ranking analysis revealed that RMS had the highest probability of clinical and procedural success, whereas laser sheaths ranked lowest. To mitigate potential bias from varying definitions and avoid unit‐of‐analysis errors, we evaluated patient‐level clinical success and lead‐level procedural success as defined in each study, in alignment with expert consensus on lead extraction. Our findings are consistent with previous meta‐analyses. Lee et al. [29] reported a lower complete lead removal rate with laser sheaths (93%) compared with RMS (95%). Similarly, Akhtar et al. [40] found that rotational tools were superior in terms of clinical success (99.1% vs. 97.4%) and complete success per lead (97.4% vs. 95%). Notably, RMS enables prolonged application at sites with dense scar tissue without causing vascular lacerations. Its less aggressive, forward‐directed design may better preserve adjacent vasculature [19].

Despite these observations, evidence supporting the rational combination of extraction tools for optimizing clinical outcomes remains limited. Further research is necessary to establish evidence‐based guidelines for tool selection and sequencing in clinical practice [37, 41, 42]. Although primary laser sheath use is associated with higher complication rates, attributing these solely to the device is reductive. Additionally, the choice of tools is not randomized but determined by availability and operator experience. Operators often switch to an alternative technique when insufficient progress is encountered or when continuing with the initial attempt is deemed too risky. In real‐world practice, tool changes during the same procedure are common and may confound the attribution of procedural outcomes to a single device. Optimizing the risk‐benefit profile of TLE depends on patient selection, center and operator experience, and other contextual factors. However, high‐quality comparative studies and RCTs are necessary to establish definitive guidelines. As the risks of TLE must always be carefully weighed against potential benefits, the perceived efficacy of laser sheaths influences thresholds for determining candidacy. Identifying risk factors independently associated with major complications, mortality, and clinical failure is crucial for guiding decision‐making.

Our NMA has several limitations. (1) Most included studies were non‐randomized and retrospective, potentially introducing selection bias and confounding, thereby reducing the reliability of the findings. (2) All studies were conducted at single centers, limiting their external validity and the generalizability of the results to broader populations or diverse clinical settings. (3) Significant heterogeneity was observed both between and within studies, and several confounding factors could not be fully accounted for in our analysis, such as operator experience, center volume, lead dwell time, and the proportion of infected versus non‐infected leads. (4) The study period spanned a broad timeframe, during which extraction technologies evolved. (5) Certain treatment arms included a limited number of patients, reducing statistical power to detect significant intergroup differences. (6) Subgroup analyses based on device type, indication, or device location could not be performed due to insufficient data, thereby limiting the exploration of heterogeneity and differential effects. (7) Intraprocedural crossovers between sheath types introduced additional variability that could not be fully accounted for in the analysis. The widespread use of crossover strategies may introduce misclassification bias, as procedural and clinical success rates can reflect the combined effect of multiple tools rather than the performance of a single device. (8) Non‐powered mechanical sheaths were excluded from our analysis, which may limit the generalizability of our results to centers where these tools are still widely used as first‐line extraction strategies. (9) Simple traction cases may have biased comparisons, as these procedures are typically performed in leads with short implant durations. Additionally, lead dwell time could not be systematically stratified due to insufficient reporting in the primary studies. (10) Finally, it is essential to note that P‐score rankings represent probabilistic estimates rather than absolute measures of clinical superiority.

## 5 Conclusion

TLE is generally safe and effective. No single technique demonstrated superiority in terms of overall success. Despite longer procedural and fluoroscopy times, the femoral approach reduced major complications compared to laser sheaths. RMS ranked highest in improving clinical and procedural success, while laser sheaths had the lowest rankings for success endpoints and major complications. Nonetheless, study design heterogeneity, operator expertise, inconsistencies in periprocedural management reporting, and limited statistical power constrain the strength of these conclusions. Future research should prioritize prospective registries with standardized, detailed procedural reporting of crossover events to allow more accurate analyses.

## Author Contributions

Study conception and design: Antônio da Silva Menezes Júnior. Statistical analysis: Charles Karel Martins Santos. Acquisition and interpretation of data: Charles Karel Martins Santos, Maria Clara Ramos Miranda, and Gabriel Alves Barbosa. Visualization: Maria Clara Ramos Miranda. Drafting the article and/or revising it critically for important intellectual content: Charles Karel Martins Santos, Maria Clara Ramos Miranda, Gabriel Alves Barbosa, and Antônio da Silva Menezes Júnior. Final approval of the version to be submitted: Charles Karel Martins Santos, Maria Clara Ramos Miranda, Gabriel Alves Barbosa, and Antônio da Silva Menezes Júnior.

## Conflicts of Interest

The authors declare no conflicts of interest.

## Ethics Statement

This study is based on published data and did not involve human or animal subjects.

## Permission to Reproduce Material From Other Sources

No material requiring permission for reproduction from other sources was used.

## Clinical Trial Registration

The protocol of this systematic review and network meta‐analysis was previously registered with PROSPERO (CRD42024612238).

## Supporting information




**Supplemental Table 1**. Definitions and Criteria Adopted by the Included Studies.
**Supplemental Table 2**. Settings and Study Design‐Related Characteristics.
**Supplemental Table 3**. Clinical and Demographic Characteristics of the Population.
**Supplemental Table 4**. Device‐ and Extraction‐Related Characteristics.

## Data Availability

The data supporting the findings of this study are available from the corresponding author upon reasonable request.
