# Supplementary material for: Techniques for Transvenous Lead Extraction of Cardiac Implantable Electronic Devices: A Network Meta‐Analysis
Source: Pacing Clin Electrophysiol. 2025 Sep 26;48(12):1373–83. doi: 10.1111/pace.70052 (PMC12671475; doi:10.1111/pace.70052)
Supplement: Supplementary file 1 — Supplemental Table 1. Definitions and Criteria Adopted by the Included Studies. Supplemental Table 2. Settings and Study Design‐Related Characteristics. Supplemental Table 3. Clinical and Demographic Characteristics of the Population. Supplemental Table 4. Device‐ and Extraction‐Related Characteristics. [file PACE-48-1373-s001.docx]

**Supplementary Materials**

**COMPARISON OF DIFFERENT TECHNIQUES FOR TRANSVENOUS LEAD EXTRACTION OF CARDIAC IMPLANTABLE ELECTRONIC DEVICES: A SYSTEMATIC REVIEW AND NETWORK META-ANALYSIS**

Charles Karel Martins Santos, M.S.†; Maria Clara Ramos Miranda, M.S.†; Gabriel Alves Barbosa, M.S.†; Antônio da Silva Menezes Júnior, M.D., Ph.D.*†

†Medical Department, Medical Sciences and Life School, Pontifical Catholic University of Goiás, Goiânia, Goiás, Brazil

*Medical Department, Medical Faculty, Federal University of Goiás, Goiânia, Goiás, Brazil.

**CONTENTS**

[**Supplemental Methods 1.** PRISMA 2020 Checklist 5](#_Toc198806536)

[**Supplemental Methods 2.** PRISMA 2020 Checklist for Abstracts 8](#_Toc198806537)

[**Supplemental Methods 3.** Full Search Strategy for Databases 9](#_Toc198806538)

[**Supplemental Table 1.** Definitions and Criteria Adopted by the Included Studies 12](#_Toc198806539)

[**Supplemental Table 2.** Settings and Study Design-Related Characteristics 16](#_Toc198806540)

[**Supplemental Table 3.** Clinical and Demographic Characteristics of the Population 18](#_Toc198806541)

[**Supplemental Table 4.** Device- and Extraction-Related Characteristics 21](#_Toc198806542)

[**Supplemental Results 1.** Forest Plot Comparing Major Complications Between Competing Arms 26](#_Toc198806543)

[**Supplemental Results 2.** Forest Plot Comparing Clinical Success Between Competing Arms 27](#_Toc198806544)

[**Supplemental Results 3.** Forest Plot Comparing Procedural Success Between Competing Arms 28](#_Toc198806545)

[**Supplemental Results 4.** Network Graph for Procedure Time 29](#_Toc198806546)

[**Supplemental Results 5.** Network League Table for Procedure Time Comparisons 30](#_Toc198806547)

[**Supplemental Results 6.** Forest Plot Comparing Procedure Time Between Competing Arms 31](#_Toc198806548)

[**Supplemental Results 7.** Network Graph for Fluoroscopy Time 32](#_Toc198806549)

[**Supplemental Results 8.** Network League Table for Fluoroscopy Time Comparisons 33](#_Toc198806550)

[**Supplemental Results 9.** Forest Plot Comparing Fluoroscopy Time Between Competing Arms 34](#_Toc198806551)

[**Supplemental Results 10.** Beading Plot of P-score for Primary Endpoints 35](#_Toc198806552)

[**Supplemental Results 11.** Beading Plot of P-score for Secondary Endpoints 36](#_Toc198806553)

[**Supplemental Results 12.** SUCRA Rankogram for Primary and Secondary Endpoints 37](#_Toc198806554)

[**Supplemental Results 13.** Sensitivity Analysis for Primary Endpoints Restricted to Device Implant Time >12 Months 42](#_Toc198806555)

[**Supplemental Results 13A.** Sensitivity Analysis for Devices with Implant Time > 12 Months in Major Complications 42](#_Toc198806556)

[**Supplemental Results 13B.** Sensitivity Analysis for Devices with Implant Time > 12 Months in Clinical Success 43](#_Toc198806557)

[**Supplemental Results 13C.** Sensitivity Analysis for Devices with Implant Time > 12 Months in Procedural Success 44](#_Toc198806558)

[**Supplemental Results 14.** Sensitivity Analysis for Primary Endpoints Restricted to Study Sample Size >100 45](#_Toc198806559)

[**Supplemental Results 14A.** Sensitivity Analysis for Study Sample Size >100 in Major Complications 45](#_Toc198806560)

[**Supplemental Results 14B.** Sensitivity Analysis for Study Sample Size >100 in Clinical Success 46](#_Toc198806561)

[**Supplemental Results 14C.** Sensitivity Analysis for Study Sample Size >100 in Procedural Success 47](#_Toc198806562)

[**Supplemental Results 15.** Sensitivity Analysis for Primary Endpoints Excluding Studies with High/Serious Risk of Bias 48](#_Toc198806563)

[**Supplemental Results 15A.** Sensitivity Analysis for Risk of Bias in Major Complications 48](#_Toc198806564)

[**Supplemental Results 15B.** Sensitivity Analysis for Risk of Bias in Clinical Success 49](#_Toc198806565)

[**Supplemental Results 15C.** Sensitivity Analysis for Risk of Bias in Procedural Success 50](#_Toc198806566)

[**Supplemental Results 16.** Exploring heterogeneity/inconsistency for primary endpoints 51](#_Toc198806567)

[**Supplemental Results 17.** Heterogeneity/Inconsistency and Design-Based Decomposition of Cochran’s Q 54](#_Toc198806568)

[**Supplemental Results 18.** Net-Split Plot of Direct vs. Indirect Evidence for Major Complications 55](#_Toc198806569)

[**Supplemental Results 19.** Proportion of Direct and Indirect Evidence for Major Complications 56](#_Toc198806570)

[**Supplemental Results 20.** Net Heat Plot for Inconsistency Assessment in Network Meta-Analysis of Major Complications 57](#_Toc198806571)

[**Supplemental Results 21.** Net-Split Plot of Direct vs. Indirect Evidence for Clinical Success 58](#_Toc198806572)

[**Supplemental Results 22.** Proportion of Direct and Indirect Evidence for Clinical Success 59](#_Toc198806573)

[**Supplemental Results 23.** Net Heat Plot for Inconsistency Assessment in Network Meta-Analysis of Clinical Success 60](#_Toc198806574)

[**Supplemental Results 24.** Net-Split Plot of Direct vs. Indirect Evidence for Procedural Success 61](#_Toc198806575)

[**Supplemental Results 25.** Proportion of Direct and Indirect Evidence for Procedural Success 62](#_Toc198806576)

[**Supplemental Results 26.** Net Heat Plot for Inconsistency Assessment in Network Meta-Analysis of Procedural Success 63](#_Toc198806577)

[**Supplemental Results 27.** Risk of Bias Assessment 64](#_Toc198806578)

[**Supplemental Results 27A.** ROBINS-I Traffic Light Plot for Bias Assessment 64](#_Toc198806579)

[**Supplemental Results 27B.** ROBINS-I Summary Bar Plot for Bias Assessment 65](#_Toc198806580)

[**Supplemental Results 27C.** RoB 2 Traffic Light Plot for Bias Assessment 65](#_Toc198806581)

[**Supplemental Results 28.** Publication Bias Assessment 66](#_Toc198806582)

[**Supplemental Results 28A.** Comparison-Adjusted Funnel Plot for Major Complications 66](#_Toc198806583)

[**Supplemental Results 28B.** Comparison-Adjusted Funnel Plot for Clinical Success 67](#_Toc198806584)

[**Supplemental Results 28C.** Comparison-Adjusted Funnel Plot for Procedural Success 68](#_Toc198806585)

[**Supplemental References** 69](#_Toc198806586)

## **Supplemental Methods 1.** PRISMA 2020 Checklist

| **Section and Topic** | **Item #** | **Checklist item** | **Location where item is reported** |
| --- | --- | --- | --- |
| **TITLE** | | |  |
| Title | 1 | Identify the report as a systematic review. | p. 1 at MS |
| **ABSTRACT** | | |  |
| Abstract | 2 | See the PRISMA 2020 for Abstracts checklist. | Suppl. Methods 2 |
| **INTRODUCTION** | | |  |
| Rationale | 3 | Describe the rationale for the review in the context of existing knowledge. | p. 3 and 4 at MS |
| Objectives | 4 | Provide an explicit statement of the objective(s) or question(s) the review addresses. | p. 3 and 4 at MS |
| **METHODS** | | |  |
| Eligibility criteria | 5 | Specify the inclusion and exclusion criteria for the review and how studies were grouped for the syntheses. | p. 4 and 5 at MS |
| Information sources | 6 | Specify all databases, registers, websites, organisations, reference lists and other sources searched or consulted to identify studies. Specify the date when each source was last searched or consulted. | p. 5 and 6 at MS |
| Search strategy | 7 | Present the full search strategies for all databases, registers and websites, including any filters and limits used. | Suppl. Methods 3 |
| Selection process | 8 | Specify the methods used to decide whether a study met the inclusion criteria of the review, including how many reviewers screened each record and each report retrieved, whether they worked independently, and if applicable, details of automation tools used in the process. | p. 6 at MS |
| Data collection process | 9 | Specify the methods used to collect data from reports, including how many reviewers collected data from each report, whether they worked independently, any processes for obtaining or confirming data from study investigators, and if applicable, details of automation tools used in the process. | p. 6 at MS |
| Data items | 10a | List and define all outcomes for which data were sought. Specify whether all results that were compatible with each outcome domain in each study were sought (e.g. for all measures, time points, analyses), and if not, the methods used to decide which results to collect. | p. 6 at MS |
|  | 10b | List and define all other variables for which data were sought (e.g. participant and intervention characteristics, funding sources). Describe any assumptions made about any missing or unclear information. | p. 6 at MS |
| Study risk of bias assessment | 11 | Specify the methods used to assess risk of bias in the included studies, including details of the tool(s) used, how many reviewers assessed each study and whether they worked independently, and if applicable, details of automation tools used in the process. | p. 8 and 9 at MS |
| Effect measures | 12 | Specify for each outcome the effect measure(s) (e.g. risk ratio, mean difference) used in the synthesis or presentation of results. | p. 7 at MS |
| Synthesis methods | 13a | Describe the processes used to decide which studies were eligible for each synthesis (e.g. tabulating the study intervention characteristics and comparing against the planned groups for each synthesis (item #5)). | p. 7 and 8 at MS |
|  | 13b | Describe any methods required to prepare the data for presentation or synthesis, such as handling of missing summary statistics, or data conversions. | p. 7 and 8 at MS |
|  | 13c | Describe any methods used to tabulate or visually display results of individual studies and syntheses. | p. 7 and 8 at MS |
|  | 13d | Describe any methods used to synthesize results and provide a rationale for the choice(s). If meta-analysis was performed, describe the model(s), method(s) to identify the presence and extent of statistical heterogeneity, and software package(s) used. | p. 7 and 8 at MS |
|  | 13e | Describe any methods used to explore possible causes of heterogeneity among study results (e.g. subgroup analysis, meta-regression). | p. 7 and 8 at MS |
|  | 13f | Describe any sensitivity analyses conducted to assess robustness of the synthesized results. | p. 7 and 8 at MS |
| Reporting bias assessment | 14 | Describe any methods used to assess risk of bias due to missing results in a synthesis (arising from reporting biases). | p. 9 at MS |
| Certainty assessment | 15 | Describe any methods used to assess certainty (or confidence) in the body of evidence for an outcome. | NA |
| **RESULTS** | | |  |
| Study selection | 16a | Describe the results of the search and selection process, from the number of records identified in the search to the number of studies included in the review, ideally using a flow diagram. | p. 9 at MS, and Figure 1 |
|  | 16b | Cite studies that might appear to meet the inclusion criteria, but which were excluded, and explain why they were excluded. | NA |
| Study characteristics | 17 | Cite each included study and present its characteristics. | p. 9 and 10 at MS, Table 1 |
| Risk of bias in studies | 18 | Present assessments of risk of bias for each included study. | p. 15 at MS, Suppl. Results 27 |
| Results of individual studies | 19 | For all outcomes, present, for each study: (a) summary statistics for each group (where appropriate) and (b) an effect estimate and its precision (e.g. confidence/credible interval), ideally using structured tables or plots. | p. 10-13 at MS, Suppl. Results 1-9 |
| Results of syntheses | 20a | For each synthesis, briefly summarise the characteristics and risk of bias among contributing studies. | p. 10-14 at MS |
|  | 20b | Present results of all statistical syntheses conducted. If meta-analysis was done, present for each the summary estimate and its precision (e.g. confidence/credible interval) and measures of statistical heterogeneity. If comparing groups, describe the direction of the effect. | p. 10-13 at MS, Suppl. Results 1-9 |
|  | 20c | Present results of all investigations of possible causes of heterogeneity among study results. | p. 13-14 at MS |
|  | 20d | Present results of all sensitivity analyses conducted to assess the robustness of the synthesized results. | p. 13-14 at MS |
| Reporting biases | 21 | Present assessments of risk of bias due to missing results (arising from reporting biases) for each synthesis assessed. | p. 15 at MS, Suppl. Results 28 |
| Certainty of evidence | 22 | Present assessments of certainty (or confidence) in the body of evidence for each outcome assessed. | NA |
| **DISCUSSION** | | |  |
| Discussion | 23a | Provide a general interpretation of the results in the context of other evidence. | p. 15-19 at MS |
|  | 23b | Discuss any limitations of the evidence included in the review. | p. 19-20 at MS |
|  | 23c | Discuss any limitations of the review processes used. | p. 19-20 at MS |
|  | 23d | Discuss implications of the results for practice, policy, and future research. | p. 21 at MS |
| **OTHER INFORMATION** | | |  |
| Registration and protocol | 24a | Provide registration information for the review, including register name and registration number, or state that the review was not registered. | PROSPERO (CRD42024612238) |
|  | 24b | Indicate where the review protocol can be accessed, or state that a protocol was not prepared. | https://www.crd.york.ac.uk/  PROSPERO/view/CRD420  24612238 |
|  | 24c | Describe and explain any amendments to information provided at registration or in the protocol. | NA |
| Support | 25 | Describe sources of financial or non-financial support for the review, and the role of the funders or sponsors in the review. | NA |
| Competing interests | 26 | Declare any competing interests of review authors. | NA |
| Availability of data, code and other materials | 27 | Report which of the following are publicly available and where they can be found: template data collection forms; data extracted from included studies; data used for all analyses; analytic code; any other materials used in the review. | NA |

**Abbreviations:** MS, Manuscript; Suppl, Supplementary.

## **Supplemental Methods 2.** PRISMA 2020 Checklist for Abstracts

| **Section and Topic** | **Item #** | **Checklist item** | **Reported (Yes/No)** |
| --- | --- | --- | --- |
| **TITLE** | | |  |
| Title | 1 | Identify the report as a systematic review. | Yes |
| **BACKGROUND** | | |  |
| Objectives | 2 | Provide an explicit statement of the main objective(s) or question(s) the review addresses. | Yes |
| **METHODS** | | |  |
| Eligibility criteria | 3 | Specify the inclusion and exclusion criteria for the review. | Yes |
| Information sources | 4 | Specify the information sources (e.g. databases, registers) used to identify studies and the date when each was last searched. | Yes |
| Risk of bias | 5 | Specify the methods used to assess risk of bias in the included studies. | No |
| Synthesis of results | 6 | Specify the methods used to present and synthesise results. | Yes |
| **RESULTS** | | |  |
| Included studies | 7 | Give the total number of included studies and participants and summarise relevant characteristics of studies. | Yes |
| Synthesis of results | 8 | Present results for main outcomes, preferably indicating the number of included studies and participants for each. If meta-analysis was done, report the summary estimate and confidence/credible interval. If comparing groups, indicate the direction of the effect (i.e. which group is favoured). | Yes |
| **DISCUSSION** | | |  |
| Limitations of evidence | 9 | Provide a brief summary of the limitations of the evidence included in the review (e.g. study risk of bias, inconsistency and imprecision). | No |
| Interpretation | 10 | Provide a general interpretation of the results and important implications. | Yes |
| **OTHER** | | |  |
| Funding | 11 | Specify the primary source of funding for the review. | No |
| Registration | 12 | Provide the register name and registration number. | No |

## **Supplemental Methods 3.** Full Search Strategy for Databases

| **Database (Articles Retrieved)** | **Search Strategy** |
| --- | --- |
| **PubMed**  **(5,226 results)** | (("Tandem"[All Fields] OR "telescoping sheath*"[All Fields] OR "electrosurgical"[All Fields] OR "manual traction"[All Fields] OR "simple traction"[All Fields] OR "locking stylet"[All Fields] OR "powered sheath*"[All Fields] OR "laser sheath*"[All Fields] OR "excimer laser sheath*"[All Fields] OR "excimer laser system"[All Fields] OR "glidelight laser sheath*"[All Fields] OR "GlideLight"[All Fields] OR ("laser s"[All Fields] OR "lasers"[MeSH Terms] OR "lasers"[All Fields] OR "laser"[All Fields] OR "lasered"[All Fields] OR "lasering"[All Fields]) OR "laser extraction"[All Fields] OR "non-laser"[All Fields] OR "non-laser"[All Fields] OR "femoral approach"[All Fields] OR "femoral snare"[All Fields] OR "femoral sheath*"[All Fields] OR "femoral workstation"[All Fields] OR "Mechanical Dilatation"[All Fields] OR "rotating mechanical sheath*"[All Fields] OR "mechanical sheath*"[All Fields] OR "rotating sheath*"[All Fields] OR "mechanical dilator sheath*"[All Fields] OR "rotational mechanical sheath*"[All Fields] OR "mechanical extraction"[All Fields] OR "TightRail"[All Fields] OR "Evolution RL"[All Fields] OR ("rotate"[All Fields] OR "rotated"[All Fields] OR "rotates"[All Fields] OR "rotating"[All Fields] OR "rotation"[MeSH Terms] OR "rotation"[All Fields] OR "rotations"[All Fields] OR "rotational"[All Fields] OR "rotator"[All Fields] OR "rotators"[All Fields])) AND ("cardiac implantable electronic device*"[All Fields] OR "pacemaker, artificial"[MeSH Terms] OR ("pacemaker s"[All Fields] OR "pacemaker, artificial"[MeSH Terms] OR ("pacemaker"[All Fields] AND "artificial"[All Fields]) OR "artificial pacemaker"[All Fields] OR "pacemaker"[All Fields] OR "pacemakers"[All Fields] OR "pacemaking"[All Fields]) OR "pacing lead*"[All Fields] OR "cardiac pacing, artificial"[MeSH Terms] OR "defibrillator lead*"[All Fields] OR "implantable defibrillator*"[All Fields] OR "defibrillators, implantable"[MeSH Terms] OR "Cardiac Resynchronization Therapy"[MeSH Terms] OR "Cardiac Resynchronization Therapy"[All Fields] OR "lead Extraction"[All Fields] OR "transvenous Lead Extraction"[All Fields] OR "Transvenous Extraction"[All Fields] OR "device removal"[MeSH Terms] OR "device removal"[All Fields] OR "Transvenous Lead Removal"[All Fields])) OR (("extract"[All Fields] OR "extract s"[All Fields] OR "extractabilities"[All Fields] OR "extractability"[All Fields] OR "extractable"[All Fields] OR "extractables"[All Fields] OR "extractant"[All Fields] OR "extractants"[All Fields] OR "extracted"[All Fields] OR "extractibility"[All Fields] OR "extractible"[All Fields] OR "extracting"[All Fields] OR "extraction"[All Fields] OR "extractions"[All Fields] OR "extractive"[All Fields] OR "extractives"[All Fields] OR "extracts"[All Fields] OR "Transvenous Extraction"[All Fields] OR "lead Extraction"[All Fields] OR "transvenous Lead Extraction"[All Fields] OR "device removal"[MeSH Terms] OR "device removal"[All Fields] OR "Transvenous Lead Removal"[All Fields]) AND ("cardiac implantable electronic device*"[All Fields] OR "pacemaker, artificial"[MeSH Terms] OR ("pacemaker s"[All Fields] OR "pacemaker, artificial"[MeSH Terms] OR ("pacemaker"[All Fields] AND "artificial"[All Fields]) OR "artificial pacemaker"[All Fields] OR "pacemaker"[All Fields] OR "pacemakers"[All Fields] OR "pacemaking"[All Fields]) OR "pacing lead*"[All Fields] OR "cardiac pacing, artificial"[MeSH Terms] OR "defibrillator lead*"[All Fields] OR "implantable defibrillator*"[All Fields] OR "defibrillators, implantable"[MeSH Terms] OR "Cardiac Resynchronization Therapy"[MeSH Terms] OR "Cardiac Resynchronization Therapy"[All Fields])) |
| **Embase**  **(7,839 results)** | ('tandem'/exp OR 'tandem' OR 'telescoping sheath' OR 'electrosurgical' OR 'manual traction' OR 'simple traction' OR 'locking stylet'/exp OR 'locking stylet' OR 'powered sheath' OR 'laser sheath'/exp OR 'laser sheath' OR 'excimer laser sheath' OR 'laser'/exp OR 'laser' OR 'excimer laser system' OR 'glidelight laser sheath' OR 'glidelight'/exp OR 'glidelight' OR 'laser extraction' OR 'non-laser' OR 'non laser' OR 'femoral approach'/exp OR 'femoral approach' OR 'femoral snare' OR 'femoral sheath'/exp OR 'femoral sheath' OR 'femoral workstation' OR 'rotating mechanical sheath' OR 'mechanical sheath' OR 'rotating sheath' OR 'mechanical dilator sheath'/exp OR 'mechanical dilator sheath' OR 'rotational mechanical sheath' OR 'tightrail'/exp OR 'tightrail' OR 'evolution rl'/exp OR 'evolution rl' OR 'evolution system' OR 'rotational' OR rotational) AND ('cardiac implantable electronic device'/exp OR 'cardiac implantable electronic device' OR 'pacemaker'/exp OR 'pacemaker' OR 'pacing lead'/exp OR 'pacing lead' OR 'defibrillator lead'/exp OR 'defibrillator lead' OR 'implantable defibrillator'/exp OR 'implantable defibrillator' OR 'cardiac resynchronization therapy'/exp OR 'cardiac resynchronization therapy' OR 'lead extraction'/exp OR 'lead extraction' OR 'transvenous lead extraction'/exp OR 'transvenous lead extraction' OR 'device removal'/exp OR 'device removal' OR 'transvenous extraction') OR (('transvenous extraction' OR 'lead extraction'/exp OR 'lead extraction' OR 'transvenous lead extraction'/exp OR 'transvenous lead extraction' OR 'device removal'/exp OR 'device removal') AND ('cardiac implantable electronic device'/exp OR 'cardiac implantable electronic device' OR 'pacemaker'/exp OR 'pacemaker' OR 'pacing lead'/exp OR 'pacing lead' OR 'defibrillator lead'/exp OR 'defibrillator lead' OR 'implantable defibrillator'/exp OR 'implantable defibrillator' OR 'cardiac resynchronization therapy'/exp OR 'cardiac resynchronization therapy')) |
| **Cochrane CENTRAL**  **(192 results)** | Title Abstract Keyword (( ( Tandem OR "telescoping sheath" OR "electrosurgical" OR "Manual Traction" OR "simple traction" OR "locking stylet" OR "powered sheath" OR "laser sheath" OR "excimer laser sheath" OR "excimer laser system" OR "glideLight laser sheath" OR "GlideLight" OR "laser extraction" OR "non-laser" OR "non laser" OR laser OR "femoral approach" OR "femoral snare" OR "femoral sheath" OR "femoral workstation" OR "rotating mechanical sheath" OR "mechanical sheath" OR "rotating sheath" OR "mechanical dilator sheath" OR "rotational mechanical sheath" OR "TightRail" OR "Evolution RL" OR "Evolution system" OR rotational) AND ( "Cardiac Implantable Electronic Device" OR "pacemaker" OR "pacing lead" OR "defibrillator lead" OR "Implantable Defibrillator" OR "Cardiac Resynchronization Therapy" OR "lead Extraction" OR "transvenous Lead Extraction" OR "device removal" OR "Transvenous Extraction") ) OR ( ( "Transvenous Extraction" OR extraction OR "lead Extraction" OR "transvenous Lead Extraction" OR "device removal" ) AND ( "Cardiac Implantable Electronic Device" OR "pacemaker" OR "pacing lead" OR "defibrillator lead" OR "Implantable Defibrillator" OR "Cardiac Resynchronization Therapy" ) )) |
| **Web of Science**  **(2,568 results)** | ALL=((( ( "Tandem" OR "telescoping sheath" OR "electrosurgical" OR "Manual Traction" OR "simple traction" OR "locking stylet" OR "powered sheath" OR "laser sheath" OR "excimer laser sheath" OR "excimer laser system" OR "glideLight laser sheath" OR "GlideLight" OR "laser extraction" OR "non-laser" OR "non laser" OR laser OR "femoral approach" OR "femoral snare" OR "femoral sheath" OR "femoral workstation" OR "rotating mechanical sheath" OR "mechanical sheath" OR "rotating sheath" OR "mechanical dilator sheath" OR "rotational mechanical sheath" OR "TightRail" OR "Evolution RL" OR "Evolution system" OR rotational) AND ( "Cardiac Implantable Electronic Device" OR "pacemaker" OR "pacing lead" OR "defibrillator lead" OR "Implantable Defibrillator" OR "Cardiac Resynchronization Therapy" OR "lead Extraction" OR "transvenous Lead Extraction" OR "device removal" OR "Transvenous Extraction" ) ) OR ( ( extraction OR "Transvenous Extraction" OR "lead Extraction" OR "transvenous Lead Extraction" OR "device removal" ) AND ( "Cardiac Implantable Electronic Device" OR "pacemaker" OR "pacing lead" OR "defibrillator lead" OR "Implantable Defibrillator" OR "Cardiac Resynchronization Therapy" ) ))) |

## **Supplemental Table 1.** Definitions and Criteria Adopted by the Included Studies

| **Study** | **Outcome Definition** | **Indication Criteria** | **Time elapsed  after implantation** |
| --- | --- | --- | --- |
| **Uslu et al.  2021**^1^ | The success of the extraction procedure was confirmed by clinical and radiographic criteria. Complete procedural success was defined as the removal of all lead and pacemaker generator components from the cardiac and vascular spaces without any major complications. The regression of systemic or local infection is an important determinant of the clinical success of the procedure. Complications were determined as major or minor based on previously published guidelines. Mortality related to the procedure, emergency cardiac operation, cardiac avulsion, laceration of a major thoracic vessel, pericardial effusion requiring pericardiocentesis, and pneumothorax or hemothorax requiring chest tube placement comprised the composite outcome of major complications. | The indications for lead extraction were based on the EHRA international consensus document on how to prevent, diagnose, and treat cardiac implantable electronic device infections. | NI |
| **Jo et al.  2016**^2^ | Outcome definitions have been previously reported in the HRS/AHA 2009 consensus document. Complete procedural success is defined as the removal of all targeted leads and all lead material from the vascular space, with the absence of any permanently disabling complication or procedure-related death. Clinical success is defined as the removal of all targeted leads and lead material from the vascular space or retention of a small portion of the lead that did not negatively impact the goals of the procedure. The definitions of major and minor complications related to the procedure are presented in the HRS/AHA 2009 consensus document. Major complications are defined as those that were life-threatening or that resulted in death. Minor complications are defined as those related to the procedure which required medical intervention or additional procedural intervention | The indications for transvenous lead extraction were determined according to the HRS/AHA 2009 consensus document. | > 1 year |
| **Starck et al.  2013**^3^ | Success was defined either as complete procedural success or as clinical success as defined by the expert consensus statement of the HRS. Complete procedural success represented removal of all lead material confirmed by fluoroscopy with the absence of any permanently disabling complication or procedure-related death. Clinical success was defined as removal of all lead material or retention of a small portion of the lead that does not negatively affect the outcome goals of the procedure. Complications were described as minor or major according to the expert consensus on transvenous lead extractions of the HRS. Complication rates were calculated as the number of complications in relation to the overall number of patients treated. | Indications for lead extraction of all lead extraction procedures between 2009 and 2012 with regard to the different indication groups defined in the Expert Consensus of the HRS. | > 12 months |
| **Bordachar et al. 2010**^4^ | Complete extraction was defined as the removal of the entire lead, and partial extraction was defined as the removal of most of the lead components, except for the electrode tip or <2.0 cm of wire or insulation. Major complications were life-threatening or required a major treatment intervention, such as venous or myocardial perforation, pericardial tamponade, major tricuspid regurgitation, symptomatic pulmonary embolism, and lead migration. Minor complications included pericardial or thoracic effusion requiring no intervention, pocket hematoma, silent pulmonary embolism, and trivial tricuspid regurgitation. | NI | > 4 years |
| **Kong et al.  2015**^5^ | The success of extraction (i.e., complete extraction) was determined by means of radiographic criteria, namely, the complete removal of all lead components. Major complications were life-threatening or required a major treatment intervention, such as venous or myocardial perforation, pericardial tamponade, major tricuspid regurgitation, symptomatic pulmonary embolism, and lead migration. Minor complications included pericardial or thoracic effusion without further intervention, pocket hematoma, silent pulmonary embolism, and trivial tricuspid regurgitation. | NI | NI |
| **Zsigmond et al.  2023**^6^ | Procedural outcomes were defined in accordance with current guidelines (2018 EHRA Expert Consensus Statement). Complete procedural success was defined as the removal of all targeted leads without any remnants or any lasting or irreversible complications. Clinical success was defined as the retention of a small portion of a lead that does not negatively impact procedural goals, does not increase the risk of perforation, embolic events, perpetuation of infection, or cause any undesired outcomes. Complications were defined concordant to current guidelines: (2018 EHRA expert consensus statement on lead extraction: recommendations on definitions, endpoints, research trial design, and data collection requirements for clinical scientific studies and registries: endorsed by APHRS/HRS/LAHRS). | TLE indications were defined in accordance with current guidelines (2018 EHRA expert consensus statement on lead extraction: recommendations on definitions, endpoints, research trial design, and data collection requirements for clinical scientific studies and registries: endorsed by APHRS/HRS/LAHRS). Indications were classified as pocket infection (local signs of inflammation, without involvement of the transvenous part of the device), systemic infection (positive blood cultures, lead or valvular vegetations or clinical signs of systemic infection), and non-infectious (broken, dysfunctional lead, other complication, upgrade, etc.). | NI |
| **Misra et al.  2021**^7^ | Clinical success of TLE was the primary outcome of interest for this study and was defined as removal of all targeted leads and lead material, with any residual fragments less than 4 cm, and without any negative impact on the patient or procedural goals. | NI | NI |
| **Qin et al.  2021**^8^ | Procedure success was defined according to the criteria published by the HRS. Briefly, clinical success refers to the removal of all targeted leads and lead material from the vascular space, or retention of a small portion of the lead (<4.0 cm) that does not negatively affect the outcome goals of the procedure. Complete procedural success refers to removal of all targeted leads and all lead material from the vascular space, with the absence of any permanently disabling complication or procedure-related death. Major complications were defined as those related to the procedure that were life-threatening or resulted in death, or any unexpected event that caused persistent or significant disability, or any event that required significant surgical intervention. Complications that do not meet the major complication criteria are classified as minor complications. | NI | > 1 year |
| **Lensvelt et al.  2021**^9^ | Outcomes, including the complete and partial clinical and procedural success rates, were registered according to the definitions of the 2018 HRS Expert Consensus Statement on lead. Procedure-related complications were classified  as major or minor based on severity and reversibility. | Indications for lead extraction were based on  the 2018 HRS consensus statement. | NI |
| **Bracke et al.  2022**^10^ | The endpoints are adapted from the 2017 HRS expert consensus statement. For procedural lead success, the absence of any permanently disabling complication, procedure-related death, or any unscheduled major surgical intervention (even if followed by full recovery) was added. Clinical lead success is removal of the lead with the possible exception of a small portion (<4.0 cm) with similar restrictions. The incidence of major complications resulting from application of the tools also included those arising from their use as a backup tool. Major complications were those that posed an immediate threat to life or that resulted in death. Minor complications were all undesired adverse events that required medical intervention, including minor procedural interventions, but did not significantly affect the patient’s function. | NI | > 1 year |
| **Mazzone et al.  2013**^11^ | Complete success of the procedure was defined as the removal of all targeted leads and all lead material from the vascular space without the occurrence of any permanently disabling complication or procedure-related death. Clinical success was defined as the removal of all targeted leads and lead material from the vascular space or retention of a small portion of the lead (<4.0 cm) when the residual part did not increase the risk of perforation, embolic events, or perpetuation of infection, in the absence of complications. Major complications were defined as outcomes that were life-threatening, resulted in significant or permanent disability or death, or required surgical intervention. Minor complications were defined as events related to the procedure that required medical intervention or minor procedural intervention. | Indications for lead extraction were classified as infection, lead malfunction, upgrade of a pre-existing system, or others. | > 6 months |
| **Zhou et al.  2021**^12^ | The outcomes of TLE procedure were defined according to the 2017 HRS Expert Consensus Statement on Cardiovascular Implantable Electronic Device Lead Management and Extraction, and the 2018 EHRA Expert Consensus Statement on Lead Extraction. Major complication was defined as any of the outcomes related to the procedure, which was life‐threatening or resulted in death or any unexpected event that caused persistent or significant disability. | The indications for transvenous lead extraction were also defined by the guidelines (2018 EHRA expert consensus statement) | NI |

**Abbreviations:** EHRA, European Heart Rhythm Association; AHA, American Heart Association; HRS, Heart Rhythm Society; APHRS, Asian Pacific Heart Rhythm Society; LAHRS, Latin American Heart Rhythm Society; TLE, Transvenous Lead Extraction; NI, Not Informed.

## **Supplemental Table 2.** Settings and Study Design-Related Characteristics

| **Study** | **Design** | **Sample** | **Period** | **Country** | **Center** | **Arms** |
| --- | --- | --- | --- | --- | --- | --- |
| **Uslu et al.  2021**^1^ | non-RCT (Single-center study) | 94 patients  137 leads | 2016 to July 2019 | NI | NI | Femoral approach using gooseneck snare (n = 60 patients, 83 leads) Simple manual traction (n = 34 patients, 54 leads) |
| **Jo et al.  2016**^2^ | non-RCT  (Single-center  retrospective study) | 33 patients  60 leads | September 2008  to May 2015 | South Korea | Asan Medical Center | Gooseneck snare (n = 23 patients, 43 leads) Simple manual traction (n = 10 patients, 17 leads) |
| **Starck et al.  2013**^3^ | non-RCT  (Single-center retrospective study) | 138 leads | January 2009  to December 2012 | NI | NI | Laser extraction (n = 39 leads) Evolution sheaths (n = 99 leads) |
| **Bordachar et al. 2010**^4^ | RCT  (Single-center randomized study) | 101 patients | 2007  to February 2009 | French | Hospital of Pessac | Femoral approach (n = 51 patients) Laser Sheath (n = 50 patients) |
| **Kong et al.  2015**^5^ | non-RCT  (Single-center  retrospective study) | 76 patients 134 leads | July 2013  to July 2014 | China | Peking University  People’s Hospital | Snare group (n = 59 patients, 103 leads) Evolution group (n = 17 patients, 31 leads) |
| **Zsigmond et al.  2023**^6^ | non-RCT  (Single-center  retrospective study) | 142 patients 289 leads | January 2012  to February 2021 | Hungary | University of Szeged | Laser (n = 93 patients, 159 leads) Powered mechanical (n = 49 patients, 86 leads) |
| **Misra et al.  2021**^7^ | non-RCT (Single-center prospective study) | 575 leads | 2013 to 2019 | USA | Atrium Health Carolinas  Medical Center | Laser using Glidelight sheaths (n = 395 leads) TightRail (n = 180 leads) |
| **Qin et al.  2021**^8^ | non-RCT  (Single-center  retrospective study) | 179 patients 342 leads | January 1, 2015  to March 31, 2020 | USA | Massachusetts General  Hospital | GlideLight Laser Sheath only (n = 157 patients, 297 leads) TightRail Sheath only (n = 22 patients, 45 leads) |
| **Lensvelt et al.  2021**^9^ | non-RCT  (Single-center  retrospective study) | 45 patients 95 leads | January 2014  to December 2018 | Netherlands | Leiden University  Medical Center | Manual traction with or without locking stylet (n = 10 patients, 24 leads) TightRail Mechanical Sheath (n = 35 patients, 71 leads) |
| **Bracke et al.  2022**^10^ | non-RCT (Single-center  retrospective study) | 775 patients 1115 leads | May 1997  to August 2019 | Netherlands | Catharina Hospital | Spectranetics Laser Sheath (n = 184 patients, 190 leads) Femoral approach with Byrd Femoral  Workstation sheath and Needle’s Eye snare (n = 321 patients, 717 leads) Evolution and TightRail Sheaths (n = 270 patients, 208 leads) |
| **Mazzone et al.  2013**^11^ | non-RCT  (Single-center  retrospective study) | 121 patients 208 leads | April 2005  to August 2012 | Italy | San Raffaele Hospital | Laser system (n = 73 patients, 127 leads) Evolution system (n = 48 patients, 81 leads) |
| **Zhou et al.  2021**^12^ | non-RCT  (Single-center  retrospective study) | 746 patients | March 2013 to January 2021 | China | NI | Femoral access (n = 692 patients) Laser sheaths (n = 54 patients) |

**Abbreviations:** TLE, Transvenous Lead Extraction; RMS, Rotating Mechanical Sheaths; RCT, Randomized Controlled Trial; USA, United States of America; NI, Not Informed.

## **Supplemental Table 3.** Clinical and Demographic Characteristics of the Population

| **Study** | **Arms** | **Age, yr** | **Male sex, N (%)** | **BMI, kg/m² Mean ± SD or N (%)** | **DM, N (%)** | **HTN, N (%)** | **AF, N (%)** | **CAD, N (%)** | **HF, N (%)** | **NYHA 3-4, N (%)** | **LVEF, %** | **CKD/ESRD, N (%)** |
| --- | --- | --- | --- | --- | --- | --- | --- | --- | --- | --- | --- | --- |
| **Uslu et al.  2021**^1^ | **Total** | 59.5 ± 16.8 | 66 (70.2) | 27.7 ± 5.6 | 32 (34) | 50 (53.2) | 30 (31.9) | 38 (40.4) |  | 17 (18.1) | 43.9 ± 17.2 | 12 (12.7) |
|  | **Femoral  Approach** | 58.8 ± 18.0 | 41 (68.3) | 28.3 ± 5.9 | 22 (36.6) | 31 (51.7) | 19 (31.6) | 22 (36.6) |  | 11 (18.3) | 42.5 ± 17.8 | 8 (13.3) |
|  | **Traction** | 60.6 ± 14.7 | 25 (73.5) | 27.2 ± 5.4 | 10 (29.4) | 19 (55.9) | 11 (32.3) | 16 (47.0) |  | 6 (17.6) | 45.0 ± 18.0 | 4 (11.7) |
| **Jo et al.  2016**^2^ | **Total** | 58.1 ± 14.1 | 23 (69.7) |  | 8 (24.2) | 8 (24.2) |  | 4 (12.1) |  |  |  | 2 (6.1) |
|  | **Femoral  Approach** | 57.6 ± 13.6 | 18 (78.3) |  | 7 (30.4) | 8 (34.8) |  | 3 (13.0) |  |  |  | 2 (8.7) |
|  | **Traction** | 59.3 ± 15.9 | 5 (50) |  | 1 (10) | 0 (0.0) |  | 1 (10) |  |  |  | 0 (0.0) |
| **Starck et al.  2013**^3^ | **Total** | 60.4 (23–89) | 87 (71.3) |  |  |  |  |  |  |  |  |  |
| **Bordachar et al. 2010**^4^ | **Total** |  | 78 (77.2) |  |  |  |  |  |  |  |  |  |
|  | **Laser Sheaths** | 69 ±15 | 38 (76) |  |  |  |  |  |  |  | 57 ± 14 |  |
|  | **Femoral  Approach** | 72 ± 15 | 40 (78.4) |  |  |  |  |  |  |  | 53 ± 14 |  |
| **Kong et al.  2015**^5^ | **Total** | 68.1±14.34 | 50 (65.8) |  |  |  |  |  |  |  |  |  |
|  | **RMS** | 67.0 ± 13.0 | 10 (58.8) |  |  |  |  |  |  |  | 65.1 ± 9.6 |  |
|  | **Femoral  Approach** | 70.2 ± 9.1 | 40 (67.8) |  |  |  |  |  |  |  | 62.7 ± 6.8 |  |
| **Zsigmond et al.  2023**^6^ | **Total** | 65.4 ± 13.7 | 111 (78) |  | 38 (26.8) | 115 (81) | 52 (36.6) | 56 (39.4) | 69 (48.6) |  | 53.1 ± 17 | 14 (9.9) |
|  | **RMS** | 62.6 ± 13.8 | 41 (83.7) |  | 15 (30.6) | 44 (89.8) | 17 (34.7) | 20 (40.8) | 21 (42.9) |  | 49.2 ± 18.3 | 7 (14.3) |
|  | **Laser Sheaths** | 67 ± 13.6 | 70 (75.3) |  | 23 (24.7) | 71 (76.3) | 35 (37.6) | 36 (38.7) | 48 (51.6) |  | 55.3 ± 16 | 7 (7.5) |
| **Misra et al.  2021**^7^ | **RMS*** | 61.9 ± 16.1 | 113 (62.8) |  | 55 (30.6) | 104 (57.8) | 73 (40.6) |  |  |  |  |  |
|  | **Laser Sheaths*** | 63.9 ± 15.0 | 267 (67.6) |  | 128 (32.4) | 241 (61.0) | 152 (38.5) |  |  |  |  |  |
| **Qin et al.  2021**^8^ | **RMS** | 66.9 ± 10.6 | 14 (63.6) |  | 6 (27.3) | 9 (40.9) | 10 (45.5) |  | 12 (54.6) |  | 47.5 ± 16.7 |  |
|  | **Laser Sheaths** | 65.1 ± 14.3) | 106 (67.5) |  | 41 (26.1) | 102 (65.0) | 74 (47.1) |  | 115 (73.3) |  | 43.8 ± 16.2 |  |
| **Lensvelt et al.  2021**^9^ | **RMS** | 71 (65–79) | 28 (80) | 26 ± 5 |  | 21 (60) |  |  |  | Class 3: 1 (3) Class 4: 0 (0.0) | (≥ 55%): 8 (23) (45-54%): 7 (20) (30-44%): 15 (43) (< 30%): 5 (14) |  |
|  | **Traction** | 74 (64–79) | 9 (90) | 27 ± 3 |  | 5 (10) |  |  |  | Class 3: 3 (30)  Class 4: 0 (0.0) | (≥ 55%): 3 (30) (45-54%): 3 (30) (30-44%): 3 (30) (< 30%): 1 (10) |  |
| **Bracke et al.  2022**^10^ | **Total** | 70.3 (61–77.2) | 577 (74.5) |  |  |  |  |  |  |  |  |  |
| **Mazzone et al.  2013**^11^ | **Total** | 62.3 ± 14.4 | 95 (45.7) |  |  |  |  |  |  |  |  |  |
|  | **RMS** | 65.4 ± 14.4 | 37 (77.1) |  |  |  |  |  |  |  |  |  |
|  | **Laser Sheaths** | 60.2 ± 14.2 | 58 (79.5) |  |  |  |  |  |  |  |  |  |
| **Zhou et al. 2021**^12^ | **Total** |  | 786 (71) |  |  |  |  |  |  |  |  |  |

**Abbreviations:** RMS, Rotating Mechanical Sheaths; N, Number; yr, year(s); mo, month(s); BMI, Body Mass Index; DM, Diabetes Mellitus; HTN, Hypertension; AF, Atrial Fibrillation; CAD, Coronary Artery Disease; HF, Heart Failure; NYHA, New York Heart Association; LVEF, Left Ventricular Ejection Fraction; CKD, Chronic Kidney Disease; ESRD, End-Stage Renal Disease; SD, Standard Deviation.

## **Supplemental Table 4.** Device- and Extraction-Related Characteristics

| **Study** | **Arms** | **Number of Leads,  N** | **Dwelling Time, mon or yr Mean ± SD or median (IQR)** | **Indication,  %** | **Device type,  N (%)** | **Lead Location,  N (%)** |
| --- | --- | --- | --- | --- | --- | --- |
| **Uslu et al.  2021**^1^ | **Femoral  Approach** | 83 | 87.5 ± 37.9 mo | Pocket erosion/infection: 83.3 Lead dysfunction: 16.6 | ICD: 22 (36.6) CRT: 13 (21.6) DC-PM: 18 (30.0) SC-PM: 7 (11.6) | RV: 54 (60.7) A: 25 (28.1) CS: 10 (11.2) |
|  | **Traction** | 54 | 31.3 ± 25.8 mo | Pocket erosion/infection: 61.8 Lead dysfunction: 38.2 | ICD: 18 (52.9) CRT: 4 (11.8) DC-PM: 11 (32.4) SC-PM: 1 (2.9) | RV: 40 (58.8) A: 21 (30.9) CS: 7 (10.3) |
| **Jo et al.  2016**^2^ | **Total** | 60 | 106 (57–152) mo | Infection: 48.5 Lead malfunction: 48.5 Device upgrade: 3.0 Patient's discretion: 6.1 |  |  |
|  | **Femoral  Approach** | 43 | 121 (83–192) mo | Infection: 39.1 Lead malfunction: 47.8 Device upgrade: 4.3 Patient's discretion: 7.7 | PM: 18 (71.4) ICD: 5 (28.6) | RA: 19 (51.4) RV: 18 (48.6) |
|  | **Traction** | 17 | 56 (35–95) mo | Infection: 70 Lead malfunction: 30 Device upgrade: 0.0 Patient's discretion: 0.0 | PM 5 (50)  ICD 5 (50) | RA: 6 (54.5) RV: 5 (45.5) |
| **Starck et al.  2013**^3^ | **Total** |  | 69.6 (1–384) mo | Infection: 41.3 Chronic pain: 0.0 Thrombosis/venous stenosis: 3.9 Non-functional leads: 50.9 Functional leads: 3.9 |  |  |
|  | **RMS** |  | 95.4 (12–384) mo |  |  |  |
|  | **Laser Sheaths** |  | 83.1 (13–168) mo |  |  |  |
| **Bordachar et al. 2010**^4^ | **Laser Sheaths** | 2.3 ± 0.7* | 12 ± 6 yr | Endocarditis: 42 Pocket infection: 50 Dysfunction or upgrade: 8 | PM: 44 (88) ICD: 6 (12) |  |
|  | **Femoral  Approach** | 2.1 ± 0.6* | 13 ± 6 yr | Endocarditis: 37 Pocket infection: 49 Dysfunction or upgrade: 14 | PM: 43 (84) ICD: 8 (16) |  |
| **Kong et al.  2015**^5^ | **RMS** | 31 | 10.8 ± 7.0 yr | Endocarditis: 2 Pocket infection: 29 Dysfunction or upgrade: 0 | PM: 15 (15) ICD: 1 (2) CRT: 0 CRT-D: 1 (4) |  |
|  | **Femoral  Approach** | 103 | 11.2 ± 8.4 yr | Endocarditis: 7 Pocket infection: 94 Dysfunction or upgrade: 2 | PM: 53 (90) ICD: 2 (4) CRT: 3 (7) CRT-D: 1 (2) |  |
| **Zsigmond et al. 2023**^6^ | **Total** | 289 | 9.4 ± 6.3 yr |  | PL: 182 (74.3) DL: 50 (20.4) Single/dual coil ratio: 30/20 | RA: 93 (38.0) RV: 139 (56.7) CS: 13 (5.3) |
|  | **RMS** | 86 | 9.5 ± 7.1 yr | Pocket infection: 73.5 Systemic infection: 18.4 Non-infectious: 8.2 | PL: 64 (74.4) DL: 17 (19.8) Single/dual  coil ratio: 12/5 | CS: 5 (5.8) |
|  | **Laser Sheaths** | 159 | 9.4 ± 5.8 yr | Pocket infection: 74.2 Systemic infection: 21.5 Non-infectious: 4.3 | PL: 118 (74.2) DL: 33 (20.8) Single/dual coil ratio: 18/15 | CS: 8 (5) |
| **Misra et al.  2021**^7^ | **RMS** | 180 | ≤5 yr: 18.3% 6–10 yr: 28.9% >10 yr: 52.8% | Infection: 29.4 Non-infection: 70.6 | SC-ICD: 21 (11.7) DC-ICD: 37 (20.6) PL: 122 (67.8) | RA: 62 (34.4) RV: 109 (60.6) CS: 8 (4.4) SQ: 1 (0.6%) |
|  | **Laser Sheaths** | 395 | ≤5 yr: 23.8% 6–10 yr: 47.8% >10 yr: 28.4% | Infection: 39.7 Noninfection: 60.3 | SC-ICD: 74 (18.7) DC-ICD: 94 (23.8) PL: 227 (57.5) | RA: 107 (27.1) RV: 264 (66.8) CS: 24 (6.1) SQ: 0 (0.0) |
| **Qin et al.  2021**^8^ | **RMS** | 45 | 10.0 (2–25) yr | Isolated pocket infection: 0.0 Endocarditis: 36.4 Lead malfunction: 54.6 Device upgrade: 9.1 SVC syndrome: 0.0 | PL: 35 (77.7) SC-ICD: 5 (11.1) DC-ICD: 5 (11.1) | RA PL: 16 (35.5) RV PL: 16 (35.5) CS PL: 3 (6.7) |
|  | **Laser Sheaths** | 297 | 8.2 (1–28) yr | Isolated pocket infection: 15.9 Endocarditis: 31.2 Lead malfunction: 41.4 Device upgrade: 8.9 SVC syndrome: 2.6 | PL: 182 (61.3) SC-ICD: 38 (12.8) DC-ICD: 77 (25.9) | RA PL: 99 (33.3) RV PL: 50 (16.8) CS PL: 33 (11.1) |
| **Lensvelt et al.  2021**^9^ | **RMS** | 71 | 108 (86–155) mo | Pocket infection/decubitus: 66 Complicated bacteremia/endocarditis: 20 Lead failure: 14 | ICD: 11 (31) PM: 10 (29) CRT-D: 12 (34) CRT-P: 2 (6) Single-coil: 3 (4) Dual-coil: 18 (25) | Atrial PL: 31 (43.0) Ventricular PL: 14 (20.0) CS: 5 (7.0) |
|  | **Traction** | 24 | 80 (72–98) mo | Pocket infection/decubitus: 80 Complicated bacteremia/endocarditis: 10 Lead failure: 0.0 Other: 10 | ICD: 2 (20) PM: 2 (20) CRT-D: 6 (60) CRT-P: 0 (0) Single-coil: 2 (8) Dual-coil: 6 (25) | Atrial PL: 9 (38.0) Ventricular PL: 2 (8.0) CS: 5 (21.0) |
| **Bracke et al.  2022**^10^ | **Total** |  |  | Infection: 89.4 Non-infectious: 10.6 | ICD: 197 (25.4) CRT: 120 (15.5) |  |
|  | **RMS** | 208 | 9.6 (6.5–14.7) yr |  |  | A: 42 (20.2) V: 160 (76.9) CS: 6 (2.9) |
|  | **Laser Sheaths** | 190 | 8.1 (4.4–12.0) yr |  |  | A: 60 (31.6%) V: 130 (68.4%) CS: 0 (0.0%) |
|  | **Femoral  Approach** | 717 | 7.6 (4.5–11.2) yr |  |  | A: 328 (45.8) V: 338 (47.1) CS: 51 (7.1) |
| **Mazzone et al.  2013**^11^ | **Total** | 208 | 77.5 ± 55 mo | Infection: 76.8 Lead malfunction: 19 System upgrade: 1.7 Other indications: 2.5 |  | RA: 66 (31.7) RV PM: 37 (17.8) RV ICD: 96 (46.2) LV: 9 (4.30) |
|  | **RMS** | 81 | 101.1 ± 66.4 mo | Infection: 75 Lead malfunction: 18.8 System upgrade: 6.3 | PM: 11 (23.4) ICD: 13 (27.7) CRT-P: 1 (2.1) CRT-D: 22 (46.8) | RA: 23 (28.4) RV PM: 19 (23.5) LV: 3 (3.7) RV ICD: 36 (44.4) |
|  | **Laser Sheaths** | 127 | 62.4 ± 42.1 mo | Infection: 78.1 Lead malfunction: 19.2 System upgrade: 2.7 | PM: 13 (17.8) ICD: 25 (34.2) CRT-P: 2 (2.7) CRT-D: 33 (45.2) | RA: 43 (33.9) RV PM: 18 (14.2) LV: 6 (4.7) RV ICD: 60 (47.2) |
| **Zhou et al. 2021**^12^ | **Total** | 2382 |  | Infectious: 982 (88.9) Noninfectious: 124 (11.1) | ICD: 80 (6.9) CRT-D: 63 (5.5) PM: 917 (79.2) CRT-P: 45 (3.9) | A: 884 (37.1) RV: 1490 (62.6) CS: 108 (4.5) |

**Legend:** *: Average number of leads per patient. **Abbreviations:** V, Ventricular; A, Atrial; RA, Right Atrium; RV, Right Ventricle; CS, Coronary Sinus; LV, Left Ventricle; ICD, Implantable Cardioverter Defibrillator; CRT, Cardiac Resynchronization Therapy; DC-PM, Dual-Chamber Pacemaker; SC-PM, Single-Chamber Pacemaker; PM, Pacemaker; CRT-D, Cardiac Resynchronization Therapy Defibrillator; CRT-P, Cardiac Resynchronization Therapy Pacemaker; PL, Pacing Lead; DL, Defibrillator Lead; SC-ICD, Single-Coil Implantable Cardioverter Defibrillator; DC-ICD, Dual-Coil Implantable Cardioverter Defibrillator; mo, months; yr, years; RMS, Rotating Mechanical Sheaths.

## **Supplemental Results 1.** Forest Plot Comparing Major Complications Between Competing Arms

**
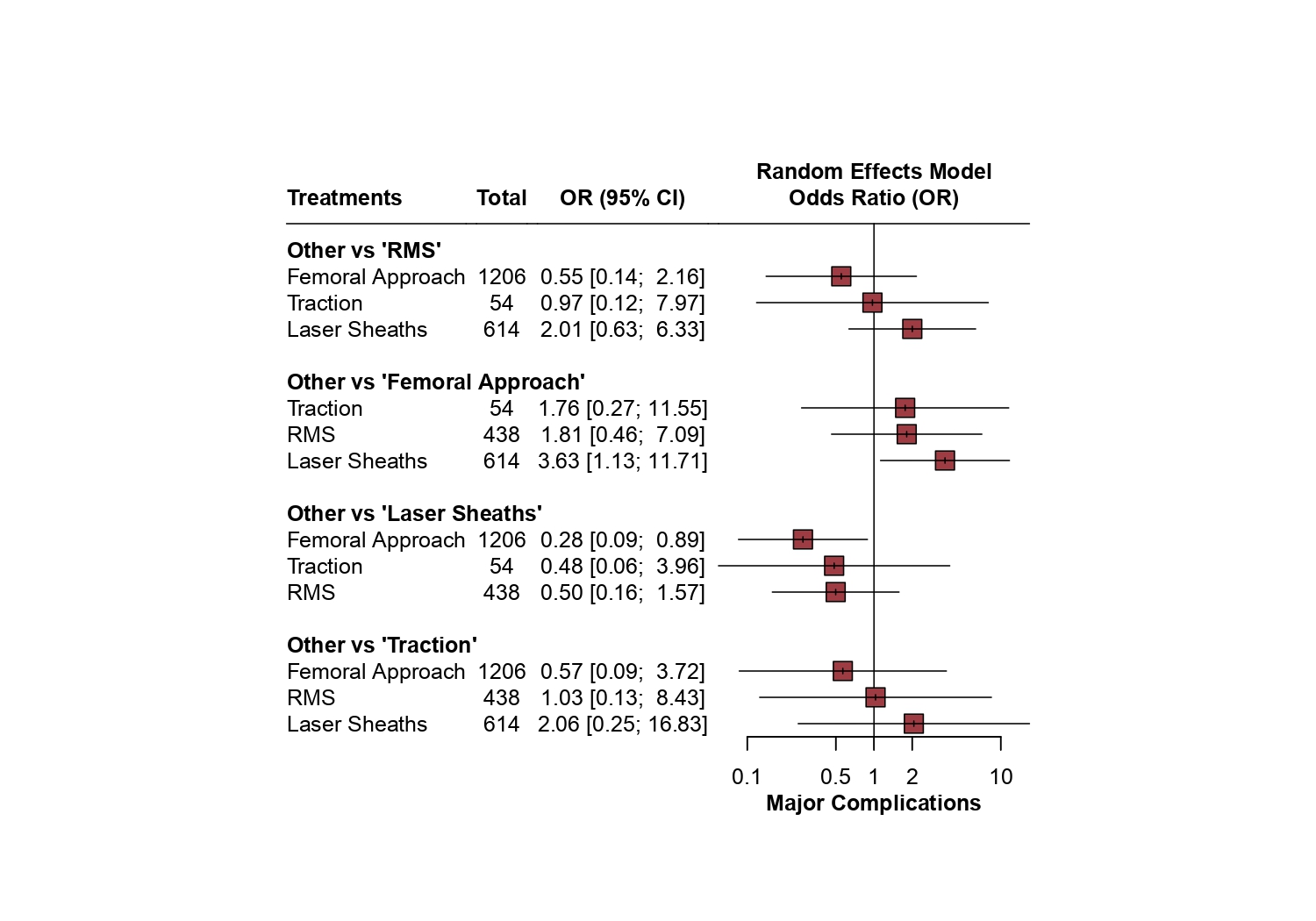
**

**Caption:** Forest plot displaying effect sizes for major complications comparing competing treatment arms. An OR less than 1 suggests that the respective intervention reduced major complications. A 95% CI excluding 1 indicates statistical significance (p < 0.05). **Abbreviations:** RMS, Rotating Mechanical Sheaths; OR, Odds Ratio; CI, Confidence Interval.

## **Supplemental Results 2.** Forest Plot Comparing Clinical Success Between Competing Arms

**
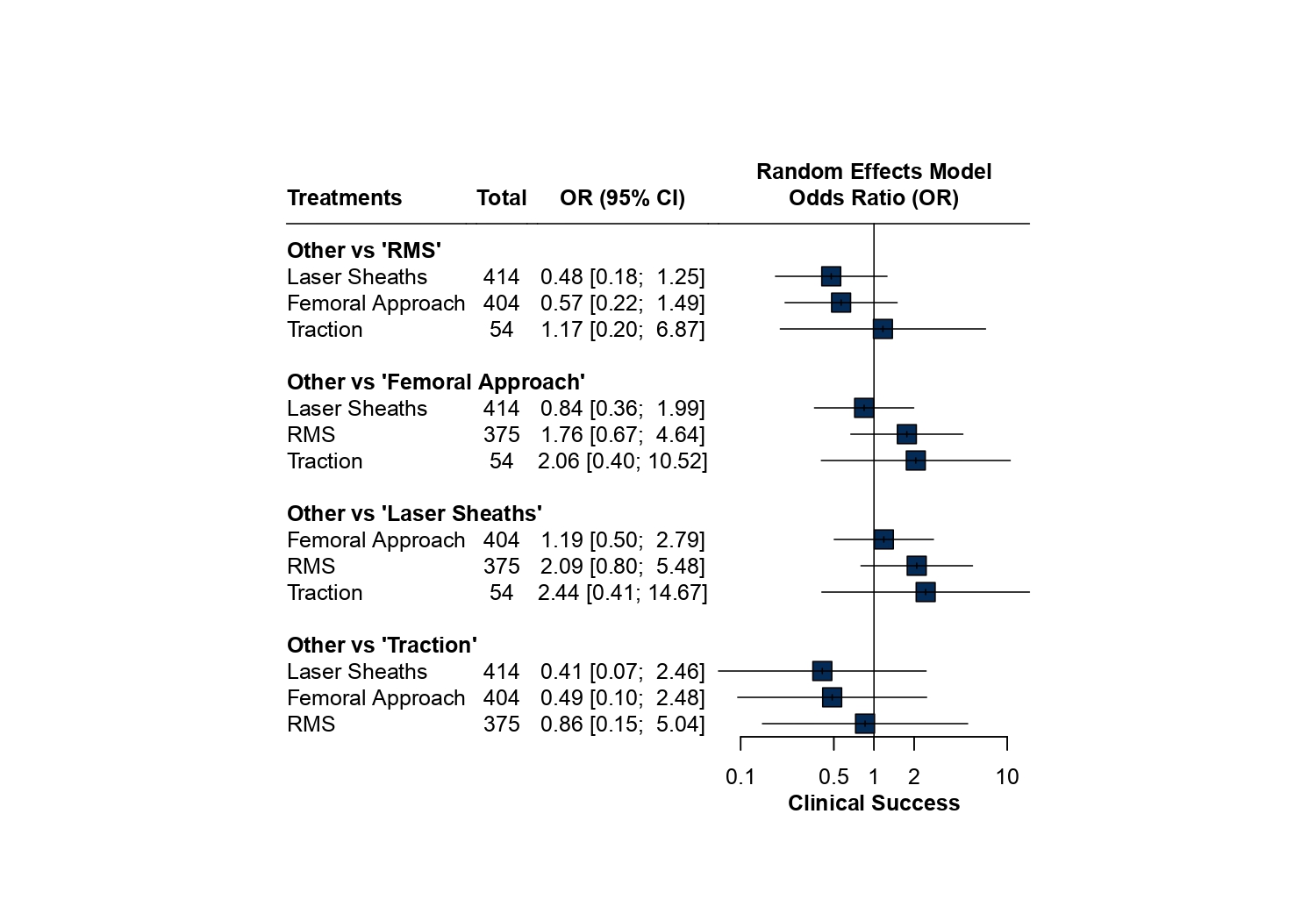
**

**Caption:** Forest plot displaying effect sizes for clinical success comparing competing treatment arms. An OR greater than 1 suggests that the respective intervention improved clinical success. A 95% CI excluding 1 indicates statistical significance (p < 0.05). **Abbreviations:** RMS, Rotating Mechanical Sheaths; OR, Odds Ratio; CI, Confidence Interval.

## **Supplemental Results 3.** Forest Plot Comparing Procedural Success Between Competing Arms


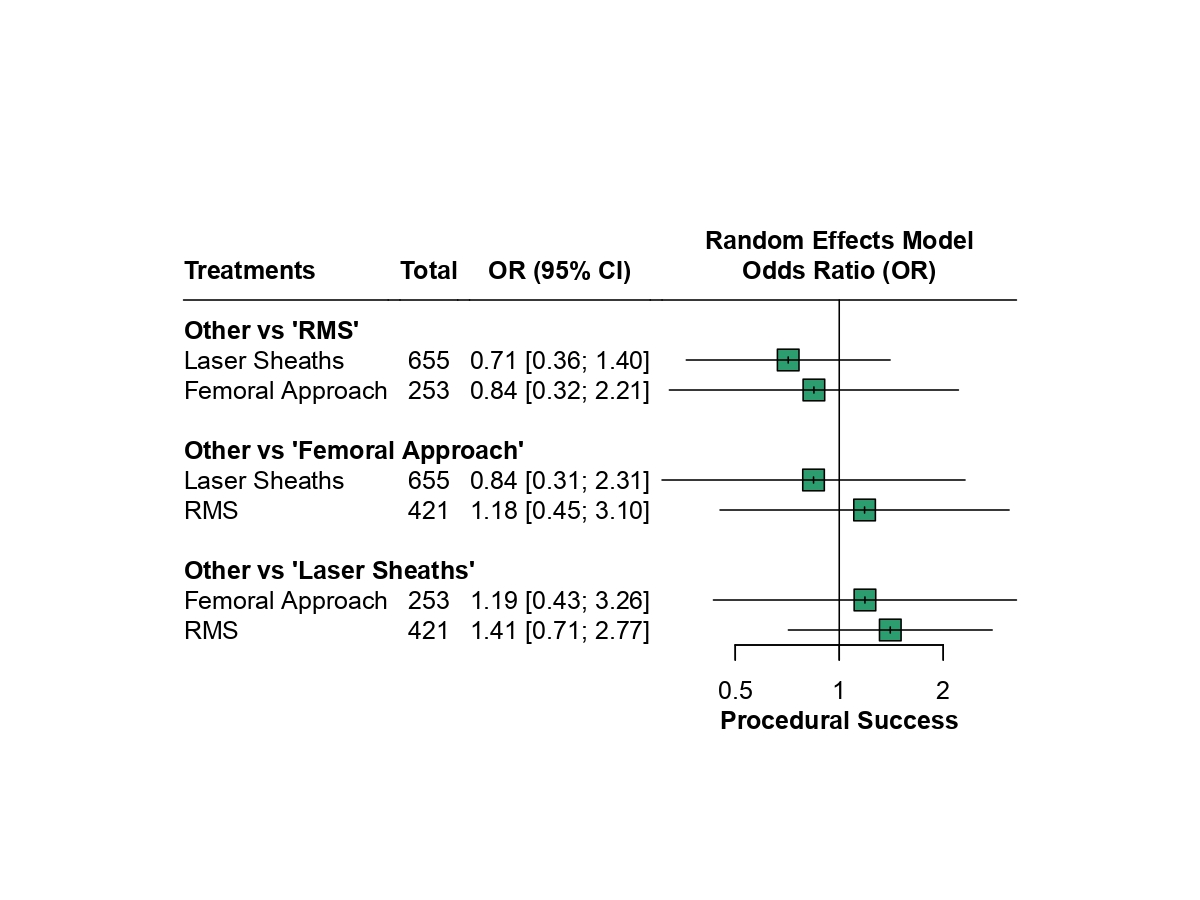


**Caption**: Forest plot displaying effect sizes for procedural success comparing competing treatment arms. An OR greater than 1 suggests that the respective intervention improved procedural success. A 95% CI excluding 1 indicates statistical significance (p < 0.05). **Abbreviations:** RMS, Rotating Mechanical Sheaths; OR, Odds Ratio; CI, Confidence Interval.

## **Supplemental Results 4.** Network Graph for Procedure Time


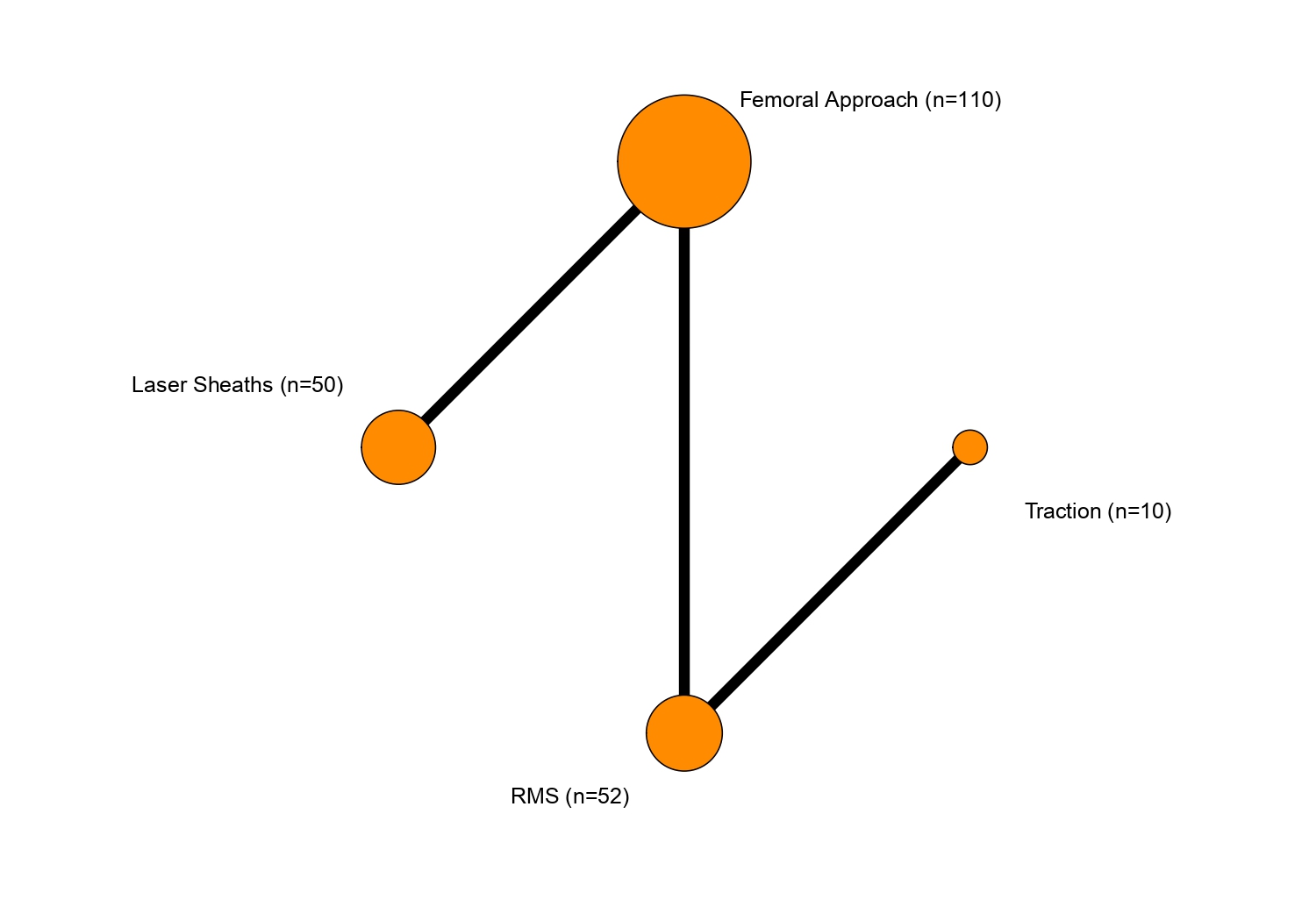


**Caption**: The nodes represent the competing interventions to be compared, and the edges represent the observed direct comparisons in the eligible studies. The size of each node is proportional to the number of patients assigned to the intervention arm. The width of the connecting lines is positively correlated with the number of studies providing direct comparisons. **Abbreviations:** RMS, Rotating Mechanical Sheaths.

## **Supplemental Results 5.** Network League Table for Procedure Time Comparisons

| **Femoral Approach** |  |  |  |
| --- | --- | --- | --- |
| **35.00 min**  **(19.73 to 50.27)** | **Laser Sheaths** |  |  |
| **31.00 min**  **(15.00 to 47.00)** | -4.00 min  (-26.12 to 18.12) | **RMS** |  |
| **92.67 min**  **(66.20 to 119.14)** | **57.67 min**  **(27.11 to 88.23)** | **61.67 min**  **(40.58 to 82.76)** | **Manual Traction** |

**Caption**: League tables showing the results of the network meta-analyses comparing the competing treatments. The table should be read from left to right. The mean difference (MD) and its corresponding 95% confidence interval (CI) for each comparison are displayed in the cell where the column-defining and row-defining treatments intersect. An MD greater than 0 means the top-left intervention increased procedure time. A 95% CI excluding 0 indicates statistical significance (p < 0.05). Statistically significant results are in bold. **Abbreviations:** RMS, Rotating Mechanical Sheaths; MD, Mean Difference; CI, Confidence Interval; min, minutes.

## **Supplemental Results 6.** Forest Plot Comparing Procedure Time Between Competing Arms


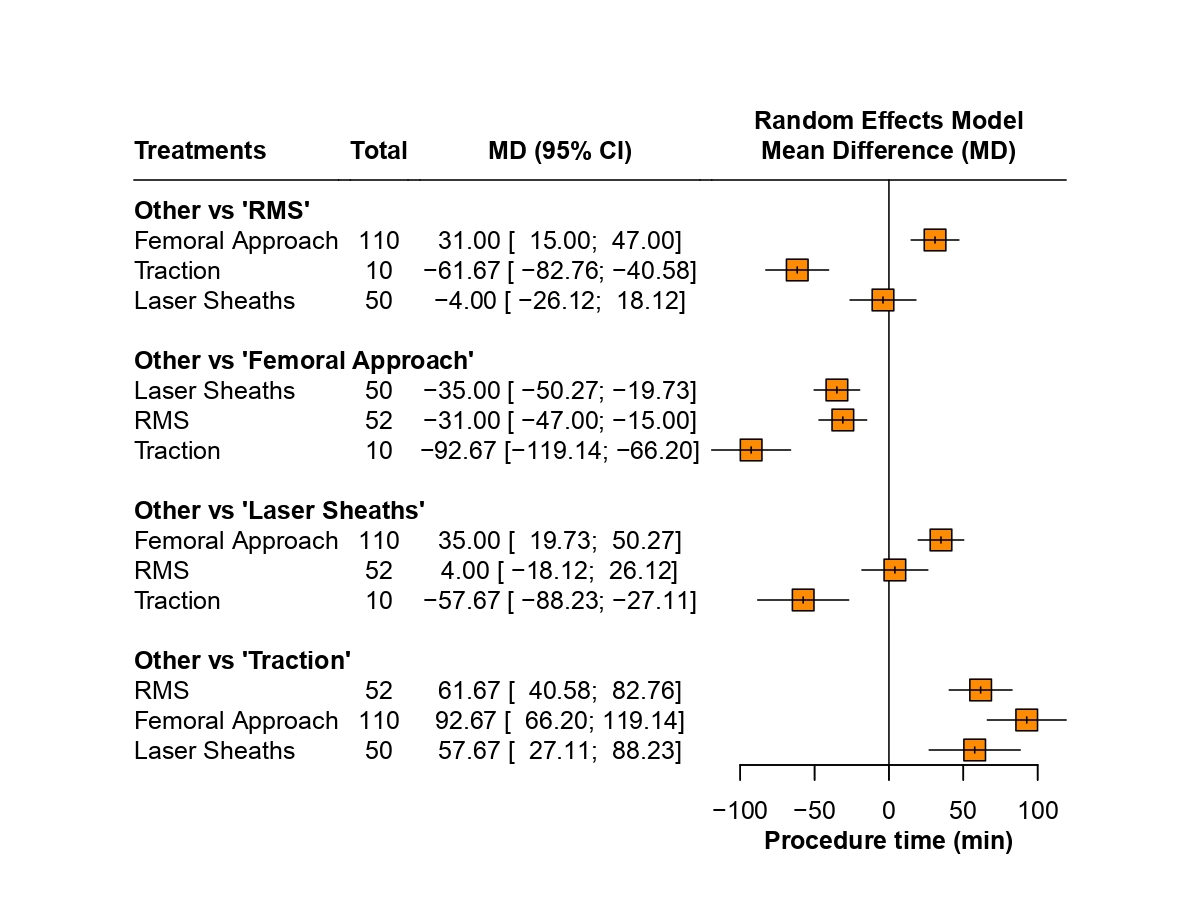


**Caption**: Forest plot displaying effect sizes for procedure time comparing competing treatment arms. An MD greater than 0 suggests that the respective intervention increased procedure time. A 95% CI excluding 0 indicates statistical significance (p < 0.05). **Abbreviations:** RMS, Rotating Mechanical Sheaths; MD, Mean Difference; CI, Confidence Interval; min, minutes.

## **Supplemental Results 7.** Network Graph for Fluoroscopy Time


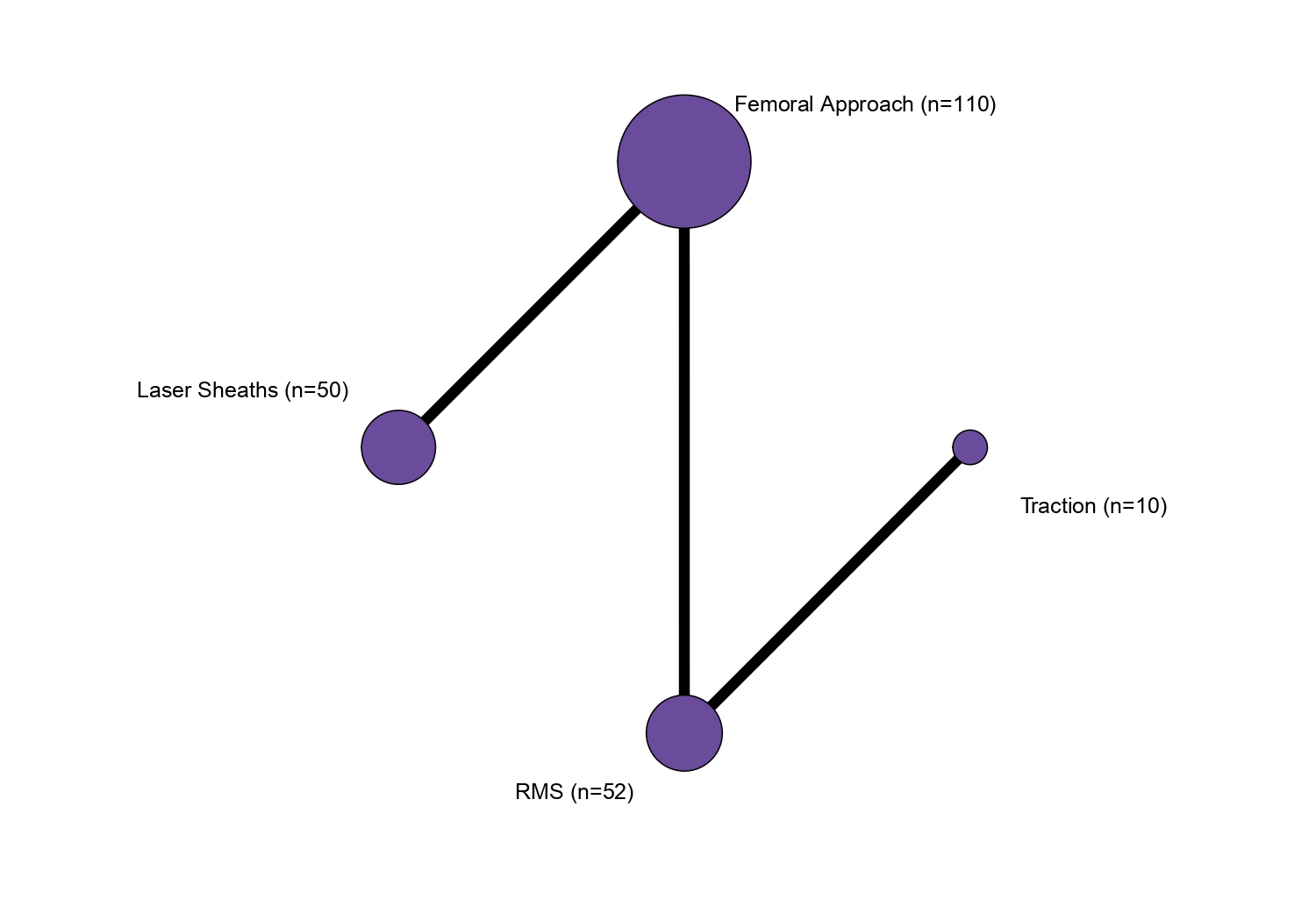


**Caption**: The nodes represent the competing interventions to be compared, and the edges represent the observed direct comparisons in the eligible studies. The size of each node is proportional to the number of patients assigned to the intervention arm. The width of the connecting lines is positively correlated with the number of studies providing direct comparisons. **Abbreviations:** RMS, Rotating Mechanical Sheaths.

## **Supplemental Results 8.** Network League Table for Fluoroscopy Time Comparisons

| **Femoral Approach** |  |  |  |
| --- | --- | --- | --- |
| **14.00 min**  **(8.95 to 19.05)** | **Laser Sheaths** |  |  |
| **12.00 min**  **(6.73 to 17.27)** | -2.00 min  (-9.30 to 5.30) | **RMS** |  |
| **15.33 min**  **(9.29 to 21.37)** | 1.33 min  (-6.54 to 9.20) | **3.33 min**  **(0.37 to 6.29)** | **Manual Traction** |

**Caption**: League tables showing the results of the network meta-analyses comparing the competing treatments. The table should be read from left to right. The mean difference (MD) and its corresponding 95% confidence interval (CI) for each comparison are displayed in the cell where the column-defining and row-defining treatments intersect. An MD greater than 0 means the top-left intervention increased fluoroscopy time. A 95% CI excluding 0 indicates statistical significance (p < 0.05). Statistically significant results are in bold. **Abbreviations:** RMS, Rotating Mechanical Sheaths; MD, Mean Difference; CI, Confidence Interval; min, minutes.

## **Supplemental Results 9.** Forest Plot Comparing Fluoroscopy Time Between Competing Arms


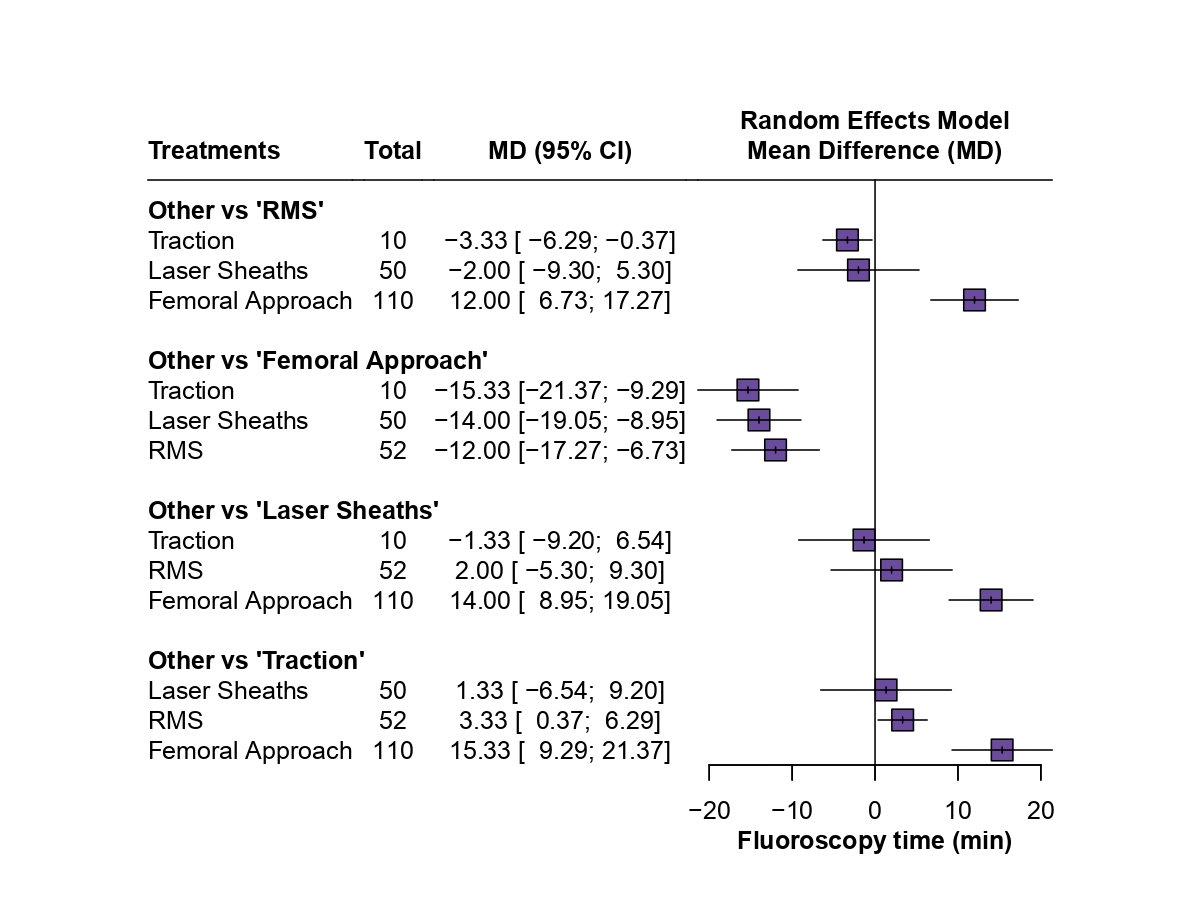


**Caption**: Forest plot displaying effect sizes for fluoroscopy time comparing competing treatment arms. An MD greater than 0 suggests that the respective intervention increased fluoroscopy time. A 95% CI excluding 0 indicates statistical significance (p < 0.05). **Abbreviations:** RMS, Rotating Mechanical Sheaths; MD, Mean Difference; CI, Confidence Interval; min, minutes.

## **Supplemental Results 10.** Beading Plot of P-score for Primary Endpoints


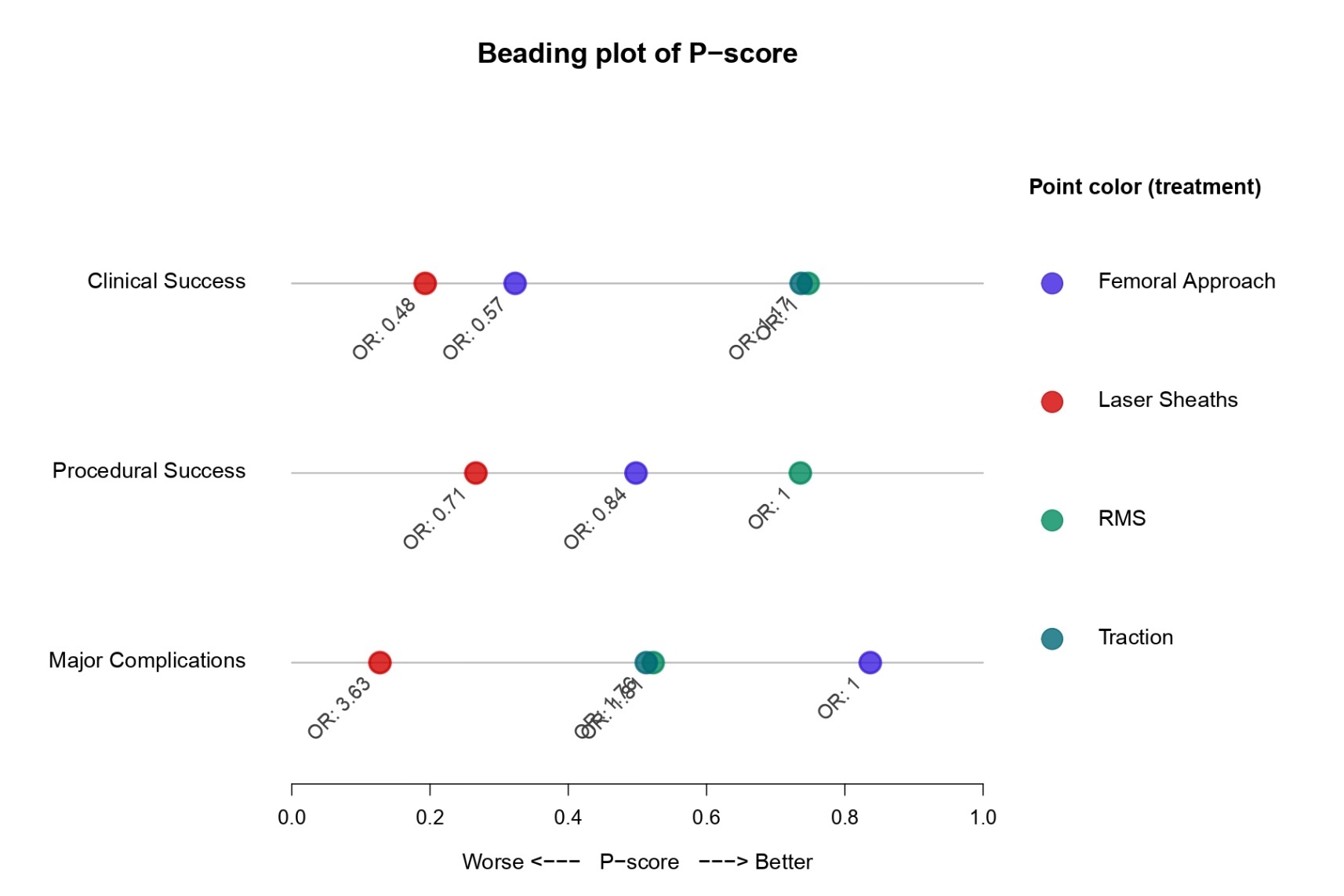


**Caption**: Beading plot of P-scores illustrating the relative ranking of treatments across different outcomes. The x-axis represents the P-score, where higher values indicate better treatment performance. Each point corresponds to a treatment-outcome pair, with colors distinguishing different treatments. The odds ratios (OR) are displayed next to each point. **Abbreviations:** RMS, Rotating Mechanical Sheaths; OR, Odds Ratio.

## **Supplemental Results 11.** Beading Plot of P-score for Secondary Endpoints


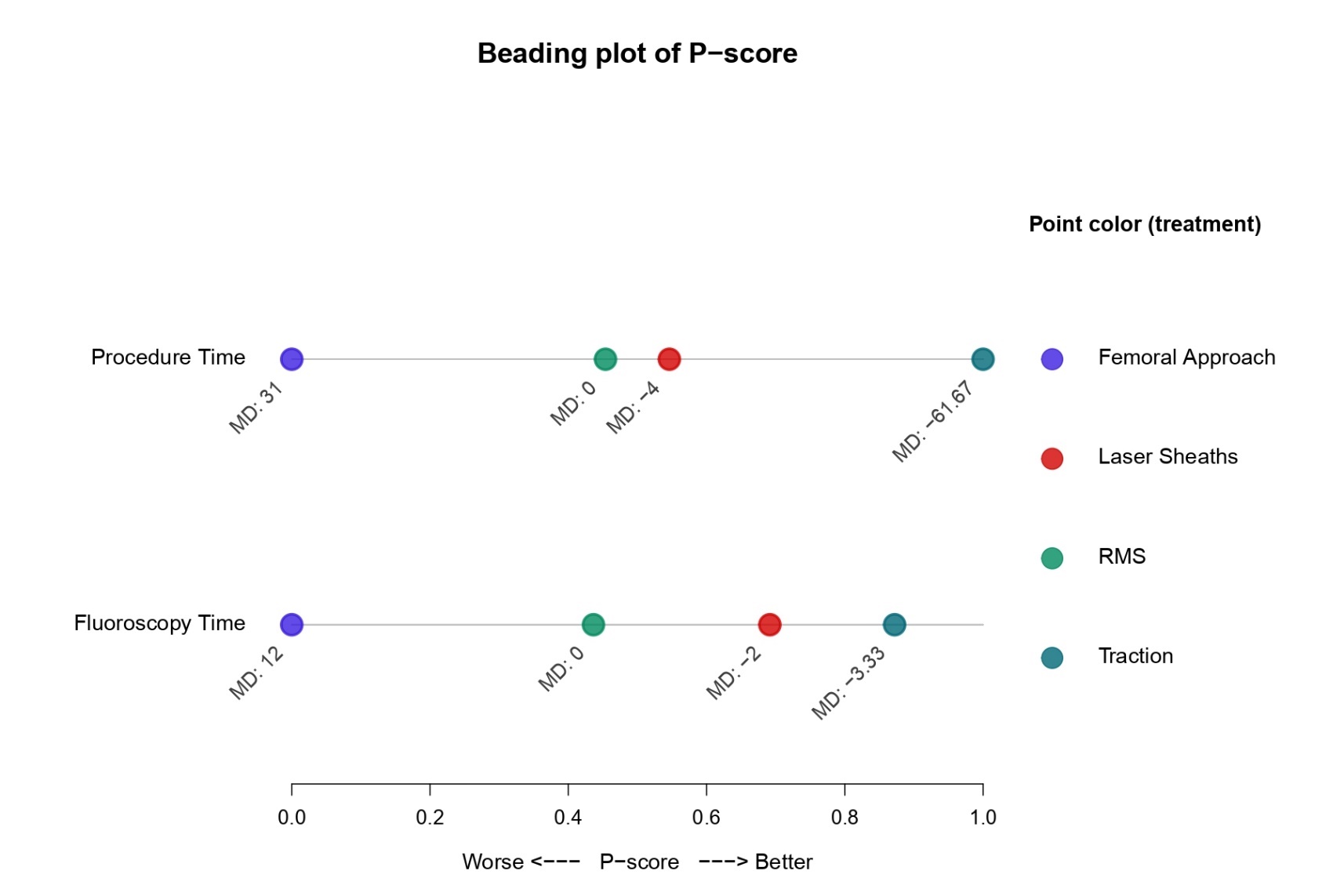


**Caption**: Beading plot of P-scores illustrating the relative ranking of treatments across different outcomes. The x-axis represents the P-score, where higher values indicate better treatment performance. Each point corresponds to a treatment-outcome pair, with colors distinguishing different treatments. The mean difference (MD) is displayed next to each point. **Abbreviations:** RMS, Rotating Mechanical Sheaths; MD, Mean Difference.

## **Supplemental Results 12.** SUCRA Rankogram for Primary and Secondary Endpoints

**Supplemental Results 12A.** Ranking Probabilities of Treatments in the Network Meta-Analysis for Major Complications


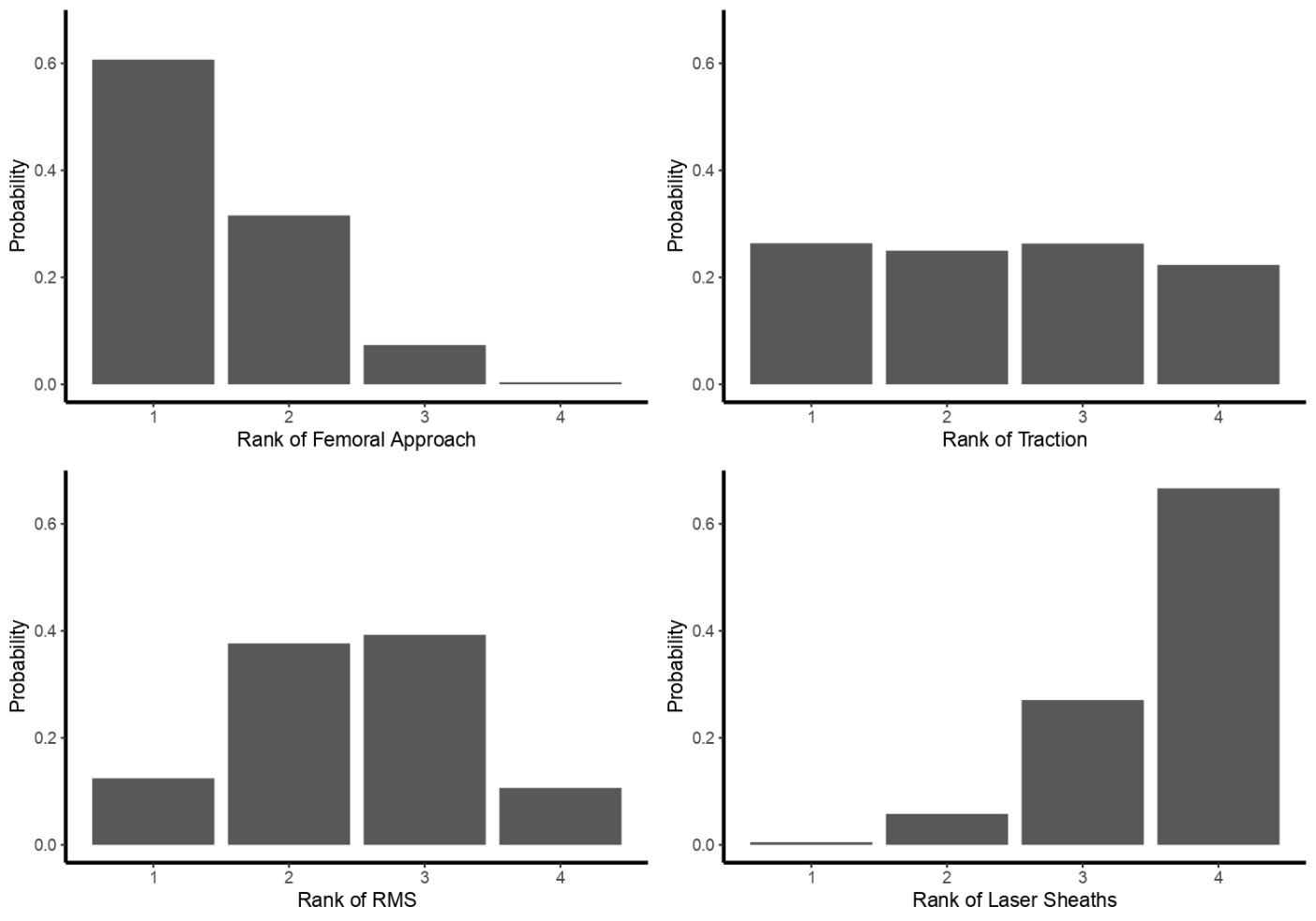


**Caption:** Rankogram displaying the probability distribution of each treatment occupying each possible rank in the major complications network. The x-axis represents the ranking positions (from best to worst), while the y-axis shows the probability of each treatment achieving a given rank. Treatments with a higher probability of ranking first are considered more effective, while a more uniform distribution indicates greater uncertainty in ranking. **Abbreviations:** RMS, Rotating Mechanical Sheaths.

**Supplemental Results 12B.** Ranking Probabilities of Treatments in the Network Meta-Analysis for Clinical Success


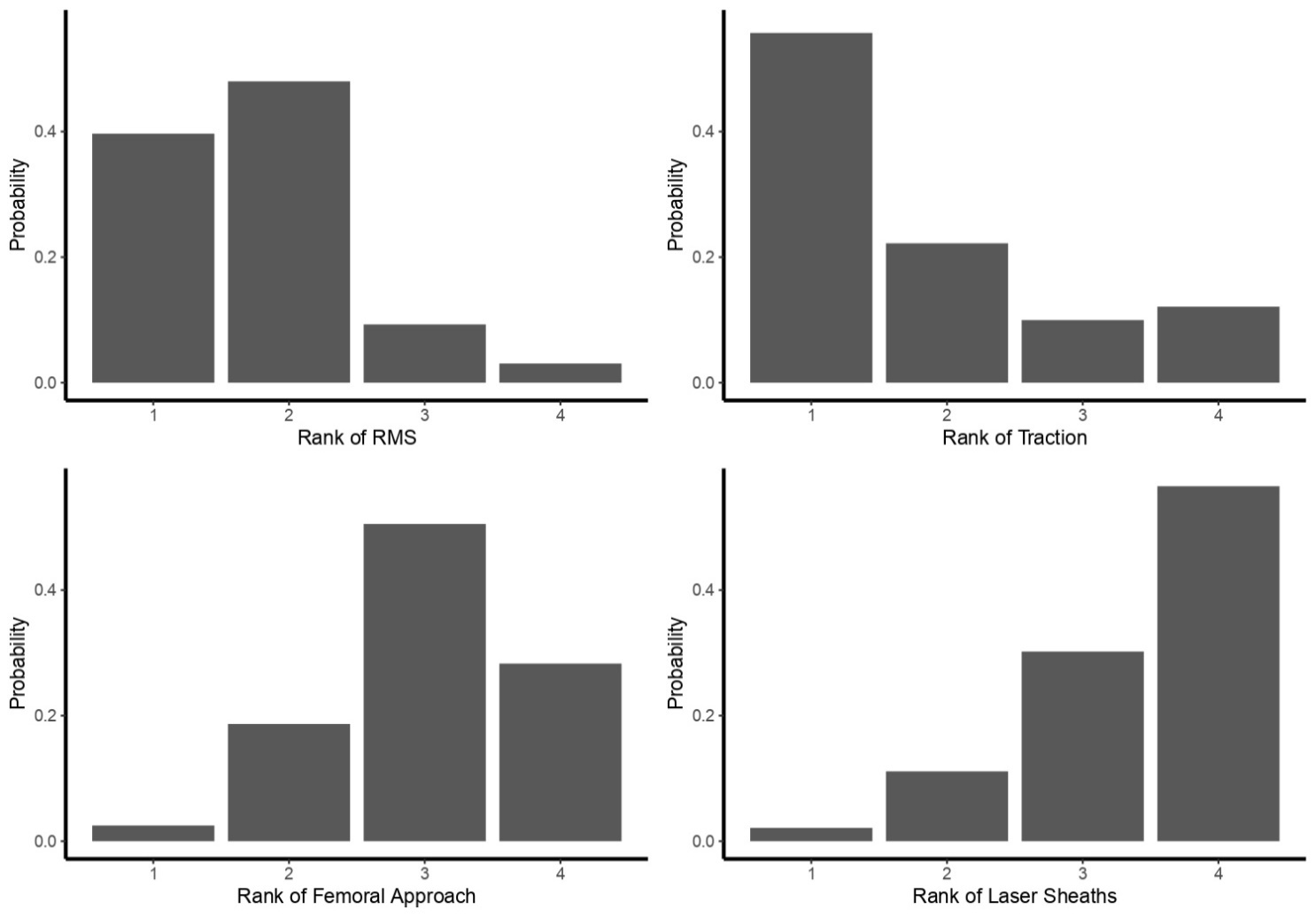


**Caption:** Rankogram displaying the probability distribution of each treatment occupying each possible rank in the clinical success network. The x-axis represents the ranking positions (from best to worst), while the y-axis shows the probability of each treatment achieving a given rank. Treatments with a higher probability of ranking first are considered more effective, while a more uniform distribution indicates greater uncertainty in ranking. **Abbreviations:** RMS, Rotating Mechanical Sheaths.

**Supplemental Results 12C.** Ranking Probabilities of Treatments in the Network Meta-Analysis for Procedural Success


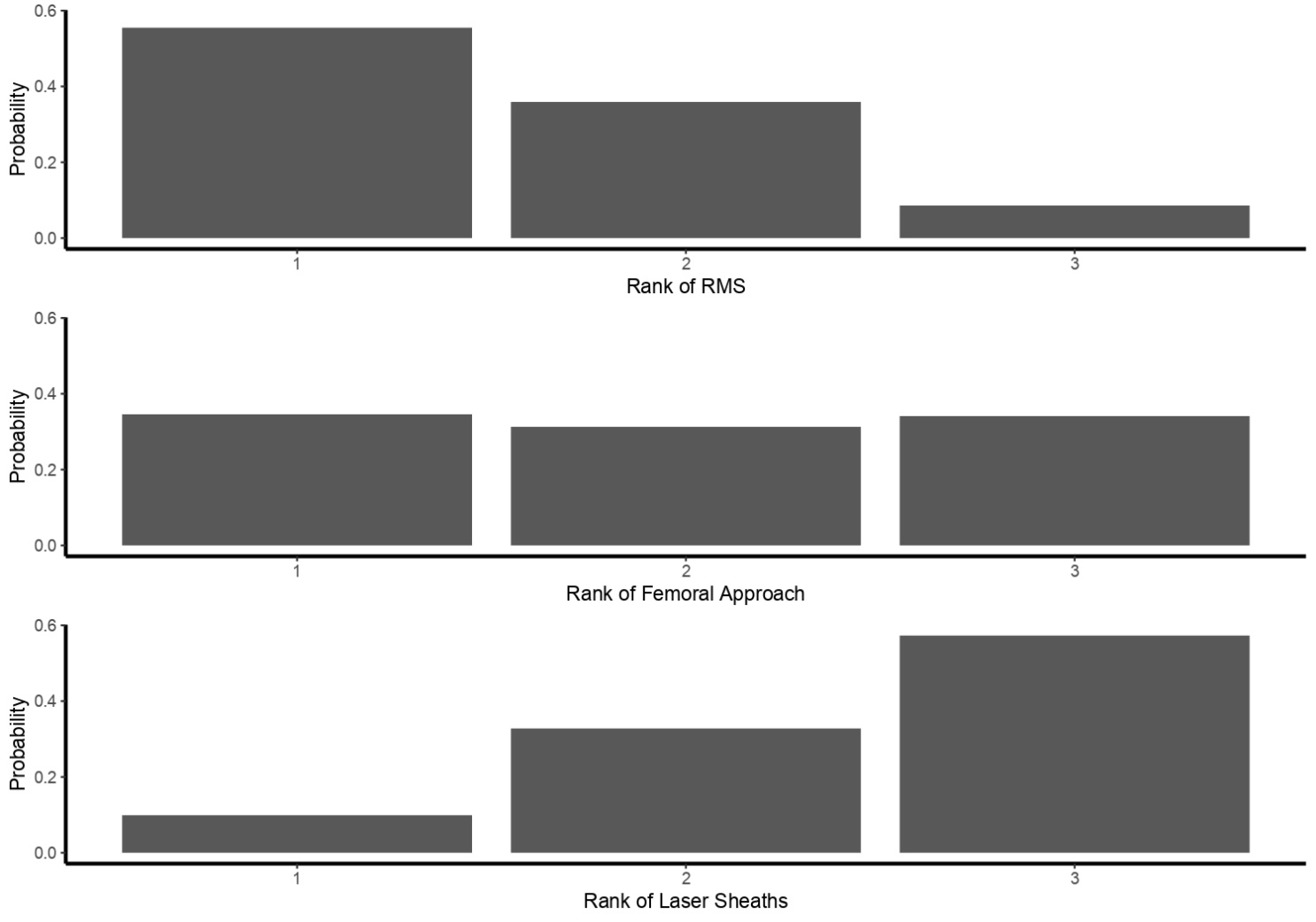


**Caption:** Rankogram displaying the probability distribution of each treatment occupying each possible rank in the procedural success network. The x-axis represents the ranking positions (from best to worst), while the y-axis shows the probability of each treatment achieving a given rank. Treatments with a higher probability of ranking first are considered more effective, while a more uniform distribution indicates greater uncertainty in ranking. **Abbreviations:** RMS, Rotating Mechanical Sheaths.

**Supplemental Results 12D.** Ranking Probabilities of Treatments in the Network Meta-Analysis for Fluoroscopy Time


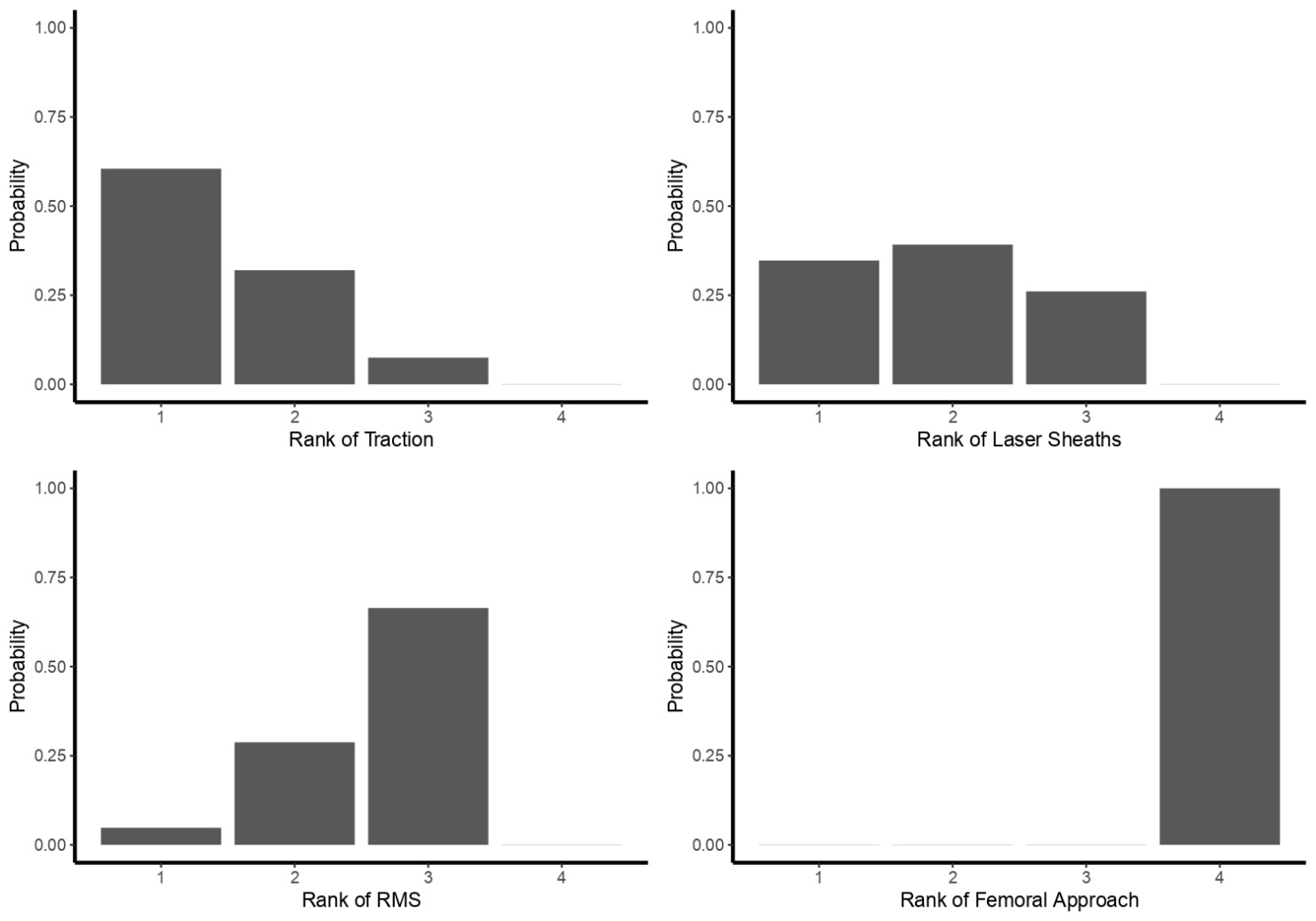


**Caption:** Rankogram displaying the probability distribution of each treatment occupying each possible rank in the fluoroscopy time network. The x-axis represents the ranking positions (from best to worst), while the y-axis shows the probability of each treatment achieving a given rank. Treatments with a higher probability of ranking first are considered more effective, while a more uniform distribution indicates greater uncertainty in ranking. **Abbreviations:** RMS, Rotating Mechanical Sheaths.

**Supplemental Results 12E.** Ranking Probabilities of Treatments in the Network Meta-Analysis for Procedure Time


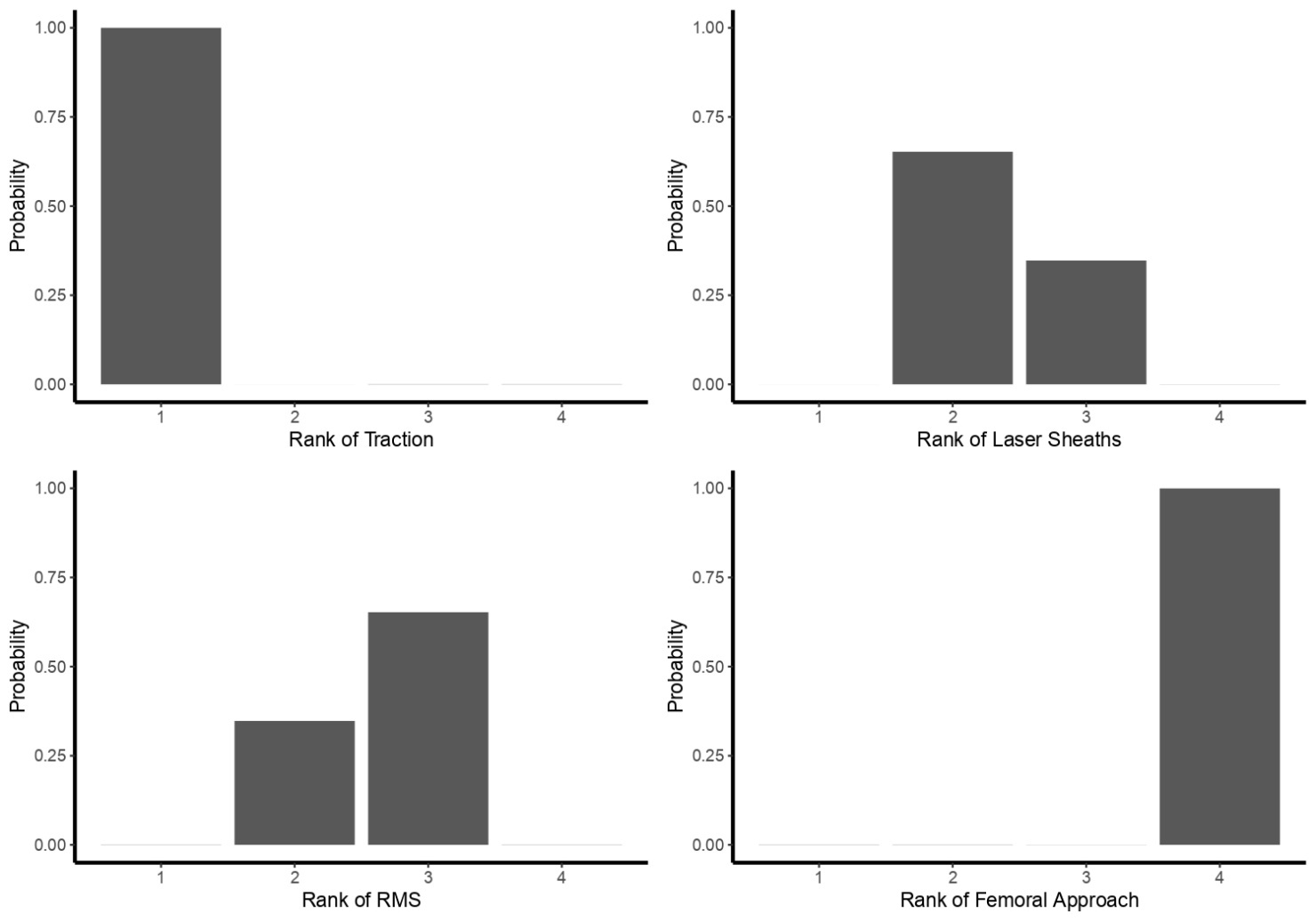


**Caption:** Rankogram displaying the probability distribution of each treatment occupying each possible rank in the procedure time network. The x-axis represents the ranking positions (from best to worst), while the y-axis shows the probability of each treatment achieving a given rank. Treatments with a higher probability of ranking first are considered more effective, while a more uniform distribution indicates greater uncertainty in ranking. **Abbreviations:** RMS, Rotating Mechanical Sheaths.

## **Supplemental Results 13.** Sensitivity Analysis for Primary Endpoints Restricted to Device Implant Time >12 Months

## **Supplemental Results 13A.** Sensitivity Analysis for Devices with Implant Time > 12 Months in Major Complications


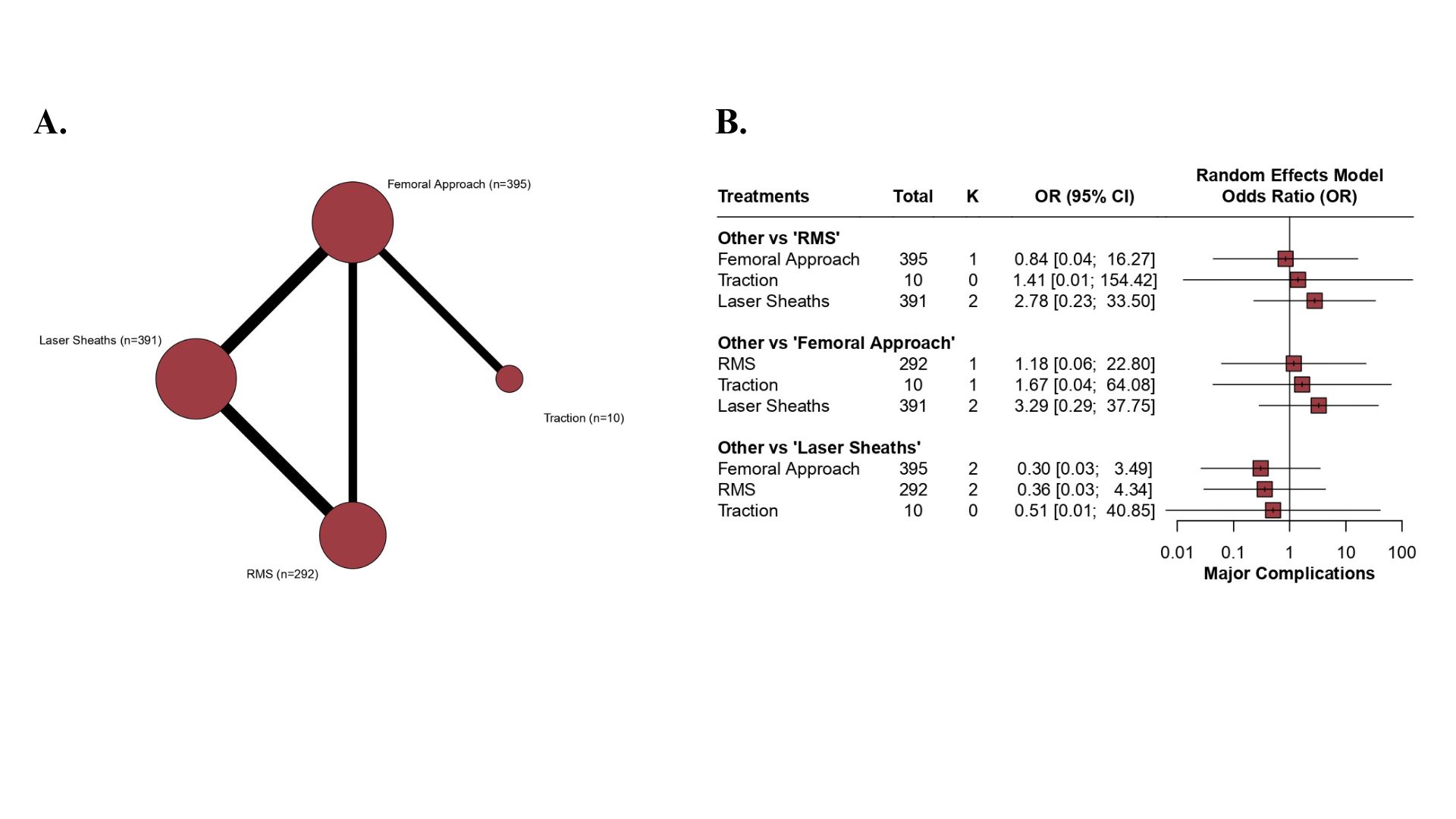


**Caption:** A. Network graph of the sensitivity analysis for devices with an implant time >12 months, assessing major complications. B. Forest plot displaying effect sizes for major complications, comparing competing treatment arms for devices with an implant time >12 months. K represents the number of studies providing direct evidence. **Abbreviations:** RMS, Rotating Mechanical Sheaths; OR, Odds Ratio.

## **Supplemental Results 13B.** Sensitivity Analysis for Devices with Implant Time > 12 Months in Clinical Success


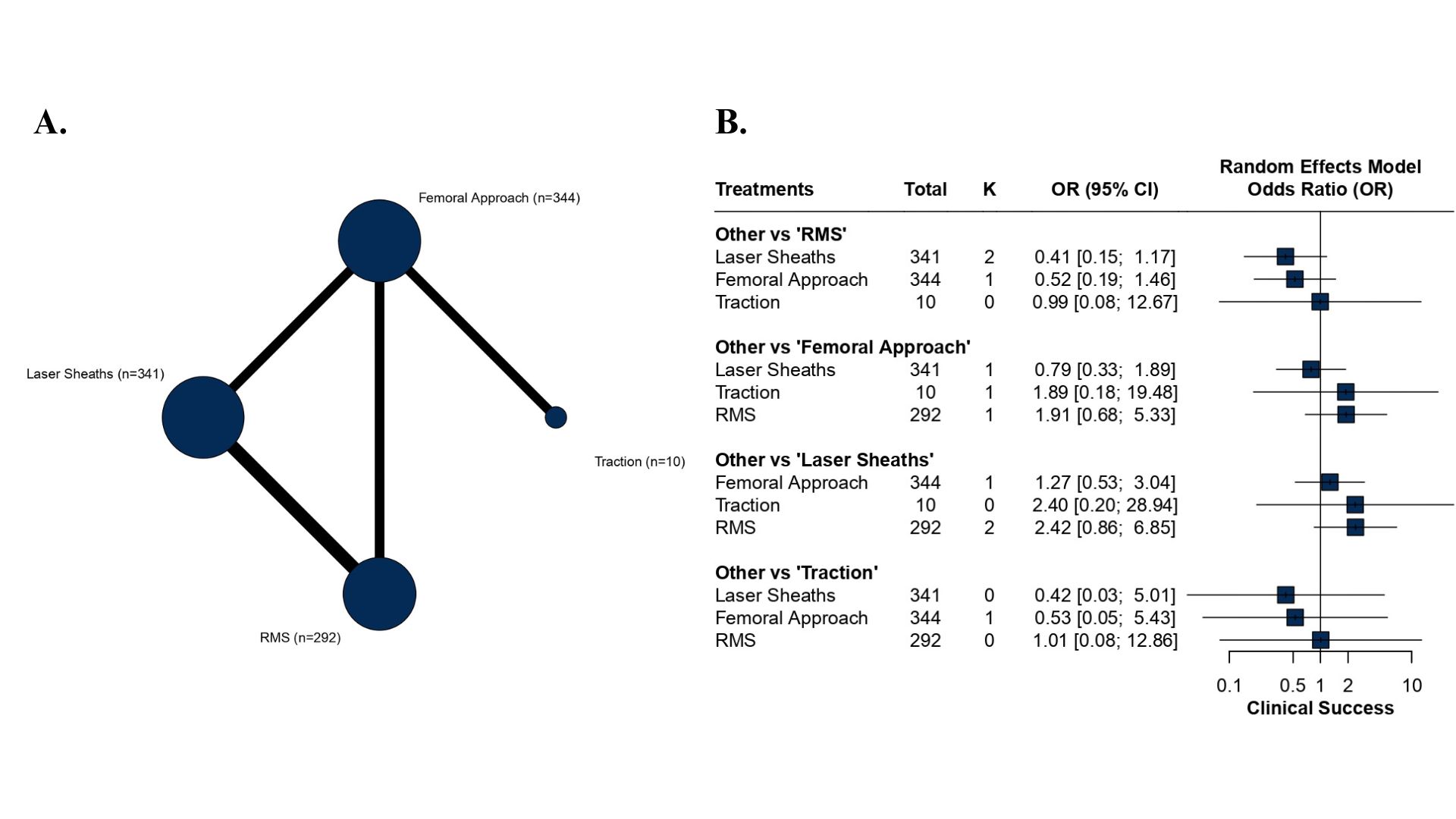


**Caption:** A. Network graph of the sensitivity analysis for devices with an implant time >12 months, assessing clinical success. B. Forest plot displaying effect sizes for clinical success, comparing competing treatment arms for devices with an implant time >12 months. K represents the number of studies providing direct evidence. **Abbreviations:** RMS, Rotating Mechanical Sheaths; OR, Odds Ratio.

## **Supplemental Results 13C.** Sensitivity Analysis for Devices with Implant Time > 12 Months in Procedural Success


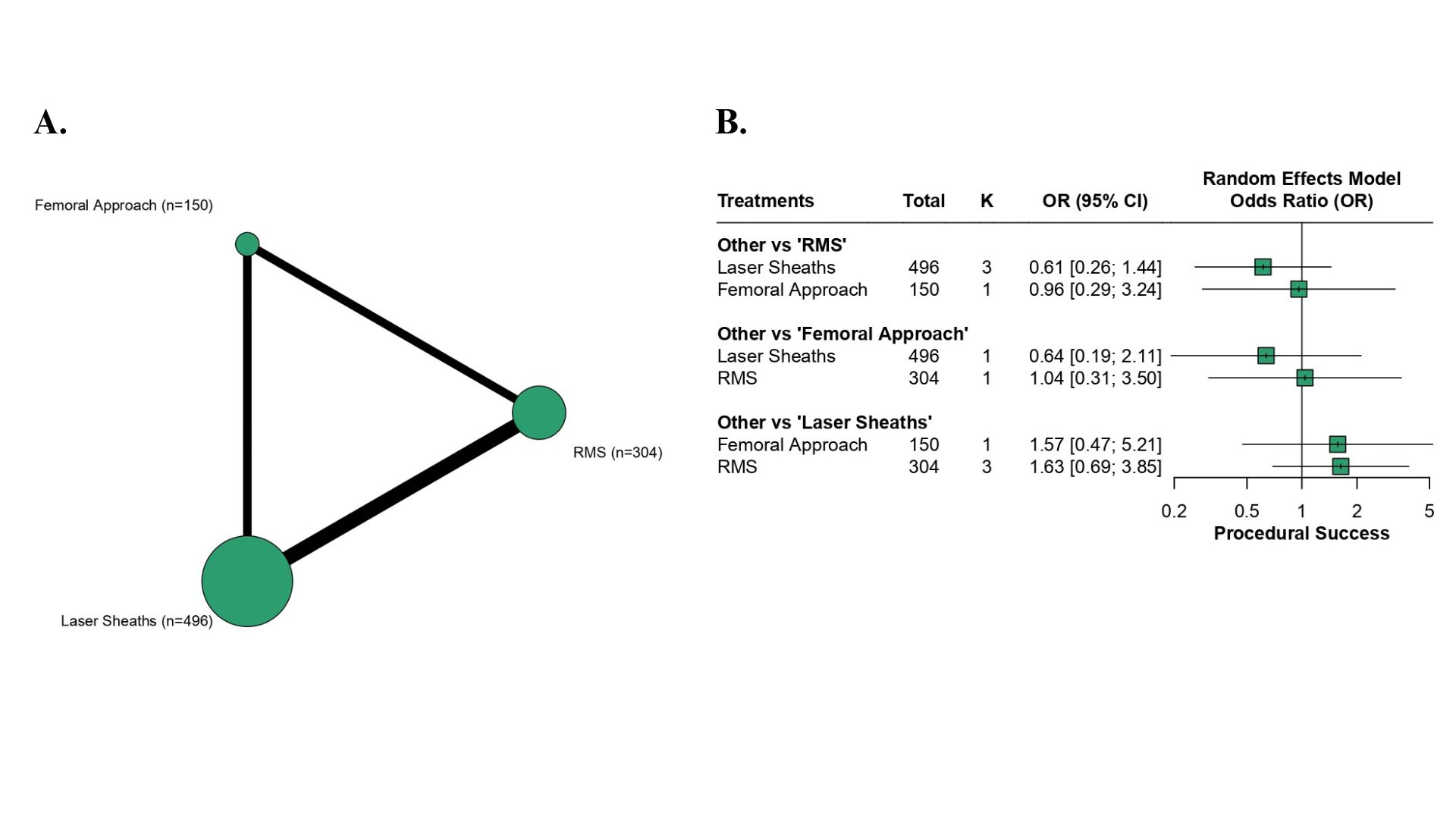


**Caption:** A. Network graph of the sensitivity analysis for devices with an implant time >12 months, assessing procedural success. B. Forest plot displaying effect sizes for procedural success, comparing competing treatment arms for devices with an implant time >12 months. K represents the number of studies providing direct evidence. **Abbreviations:** RMS, Rotating Mechanical Sheaths; OR, Odds Ratio.

## **Supplemental Results 14.** Sensitivity Analysis for Primary Endpoints Restricted to Study Sample Size >100

## **Supplemental Results 14A.** Sensitivity Analysis for Study Sample Size >100 in Major Complications


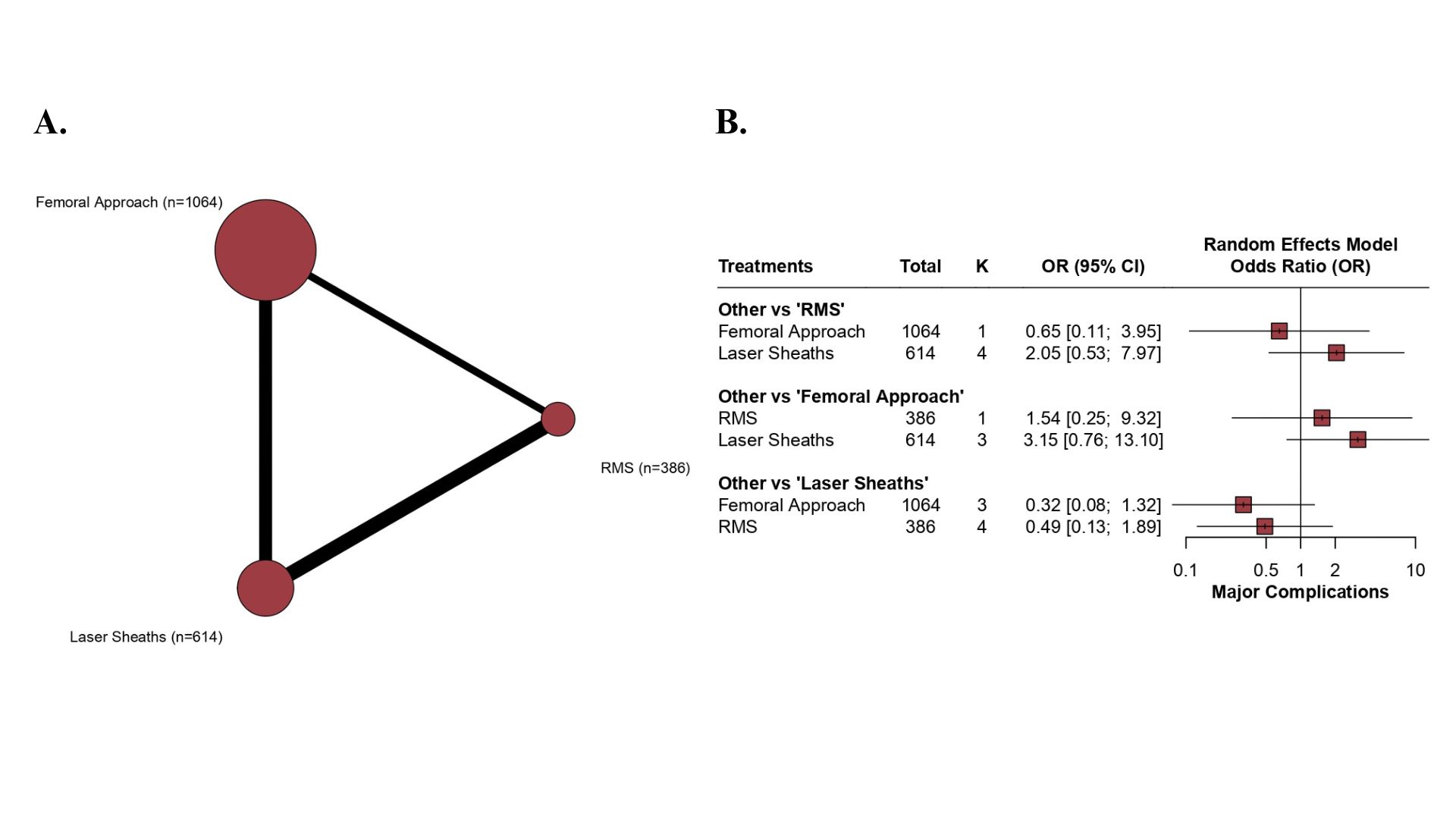


**Caption:** A. Network graph of the sensitivity analysis for study sample size >100 patients, assessing major complications. B. Forest plot displaying effect sizes for major complications, comparing competing treatment arms for study sample size >100 patients. K represents the number of studies providing direct evidence. **Abbreviations:** RMS, Rotating Mechanical Sheaths; OR, Odds Ratio.

## **Supplemental Results 14B.** Sensitivity Analysis for Study Sample Size >100 in Clinical Success


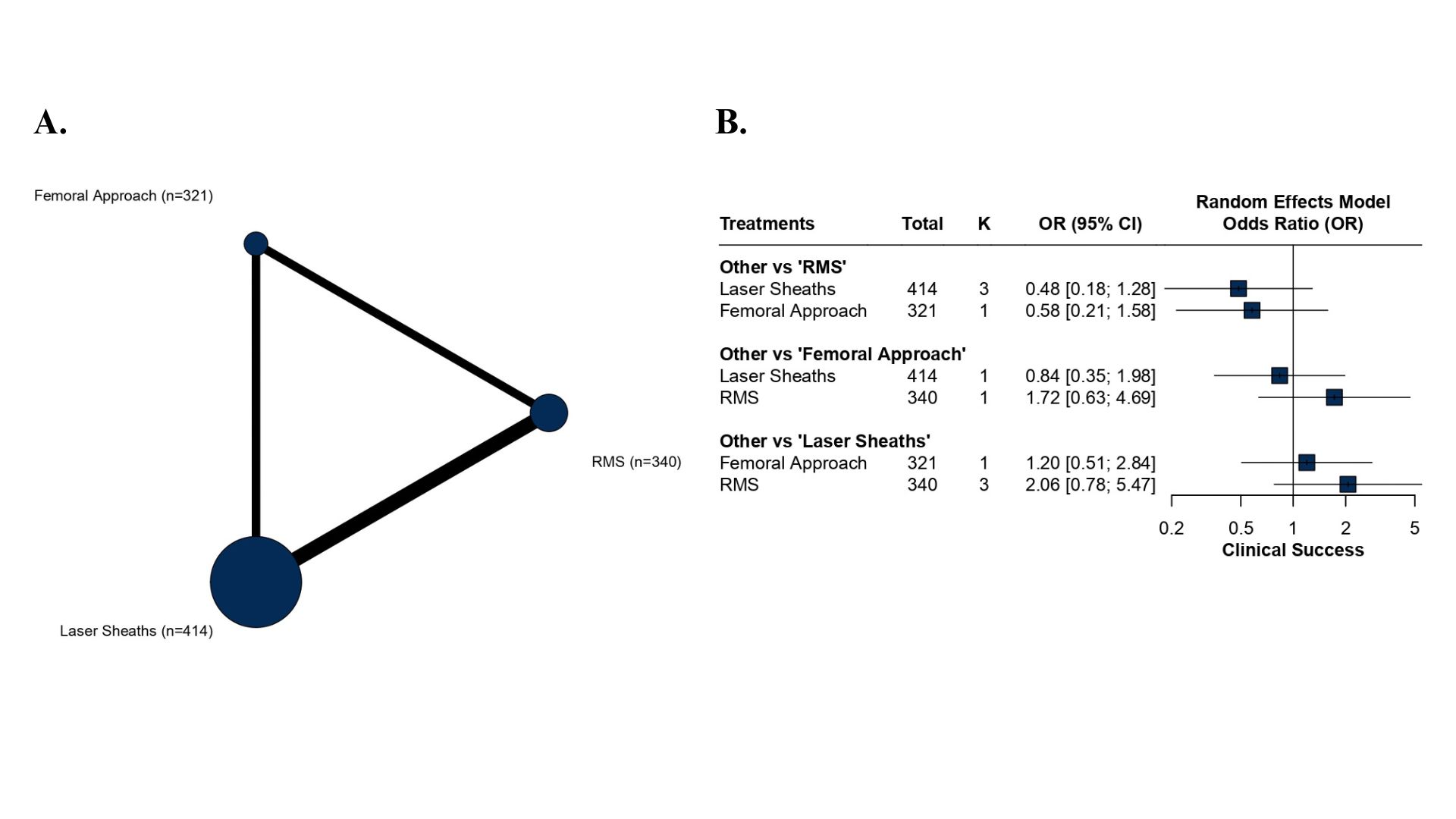


**Caption:** A. Network graph of the sensitivity analysis for study sample size >100 patients, assessing clinical success. B. Forest plot displaying effect sizes for clinical success, comparing competing treatment arms for study sample size >100 patients. K represents the number of studies providing direct evidence. **Abbreviations:** RMS, Rotating Mechanical Sheaths; OR, Odds Ratio.

## **Supplemental Results 14C.** Sensitivity Analysis for Study Sample Size >100 in Procedural Success


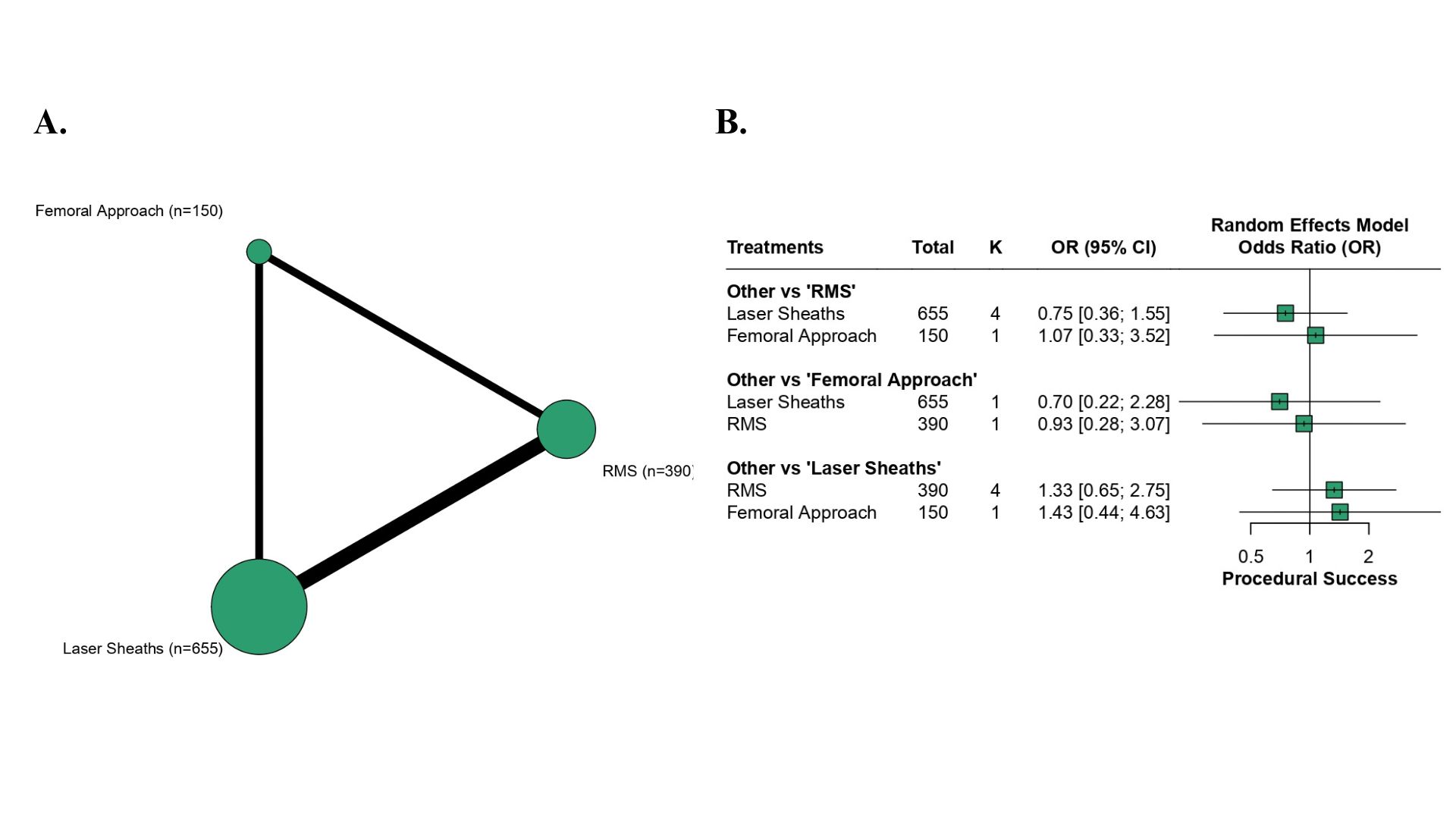


**Caption:** A. Network graph of the sensitivity analysis for study sample size >100 patients, assessing procedural success. B. Forest plot displaying effect sizes for procedural, comparing competing treatment arms for study sample size >100 patients. K represents the number of studies providing direct evidence. **Abbreviations:** RMS, Rotating Mechanical Sheaths; OR, Odds Ratio.

## **Supplemental Results 15.** Sensitivity Analysis for Primary Endpoints Excluding Studies with High/Serious Risk of Bias

## **Supplemental Results 15A.** Sensitivity Analysis for Risk of Bias in Major Complications


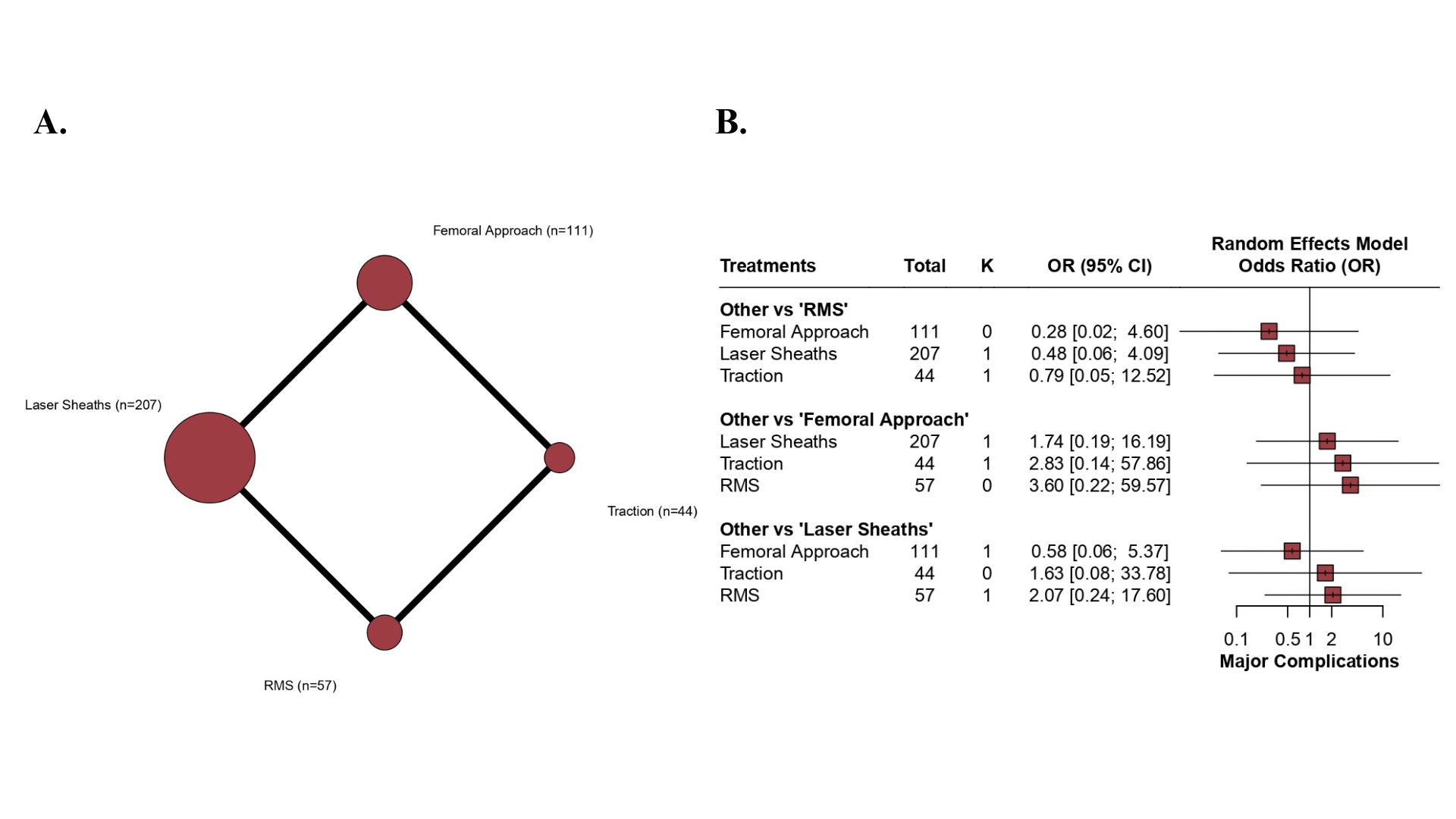


**Caption:** A. Network graph of the sensitivity analysis for risk of bias, assessing major complications. B. Forest plot displaying effect sizes for major complications, comparing competing treatment arms for risk of bias. K represents the number of studies providing direct evidence. **Abbreviations:** RMS, Rotating Mechanical Sheaths; OR, Odds Ratio.

## **Supplemental Results 15B.** Sensitivity Analysis for Risk of Bias in Clinical Success


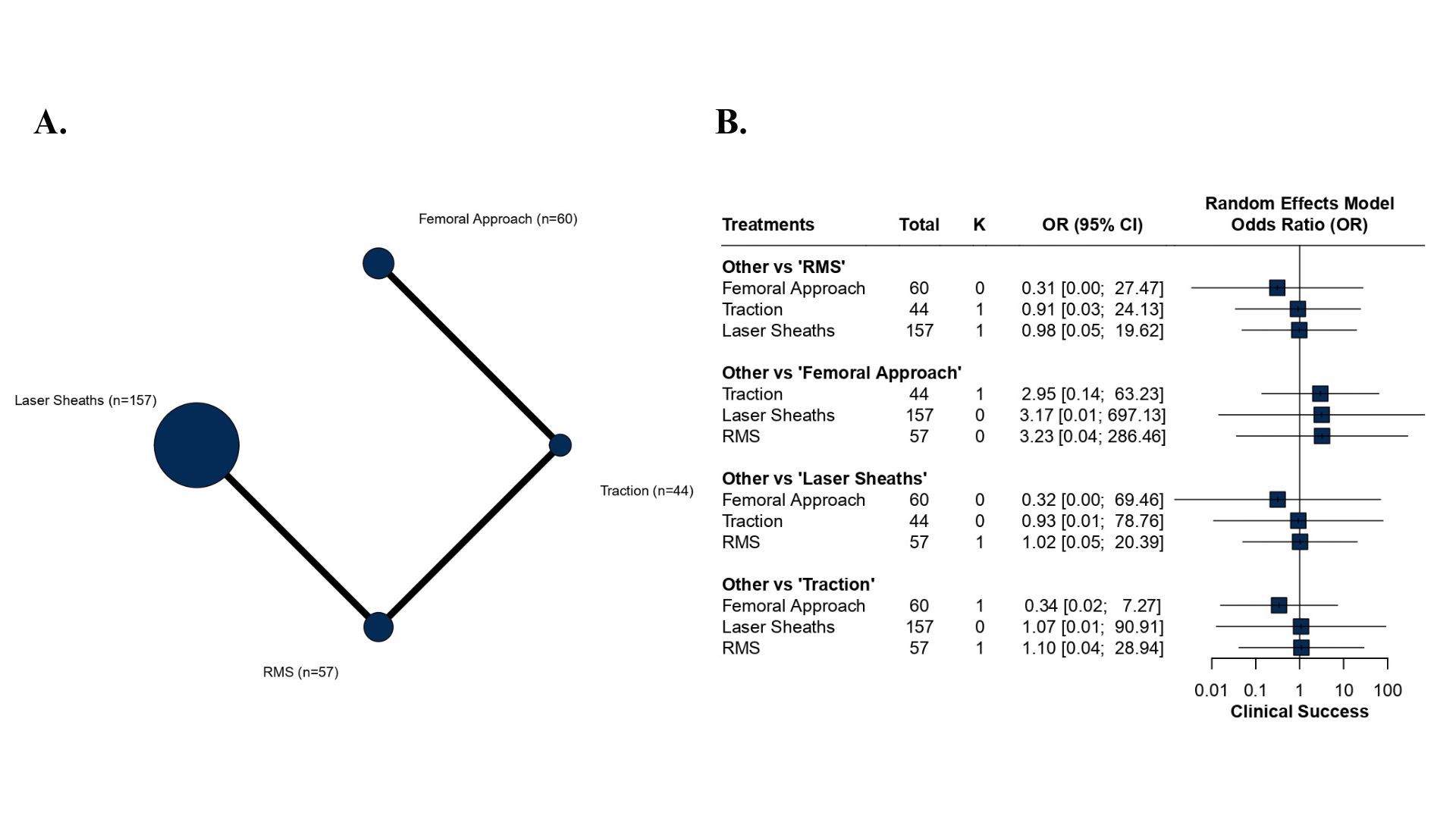


**Caption:** A. Network graph of the sensitivity analysis for risk of bias, assessing clinical success. B. Forest plot displaying effect sizes for clinical success, comparing competing treatment arms for risk of bias. K represents the number of studies providing direct evidence. **Abbreviations:** RMS, Rotating Mechanical Sheaths; OR, Odds Ratio.

## **Supplemental Results 15C.** Sensitivity Analysis for Risk of Bias in Procedural Success


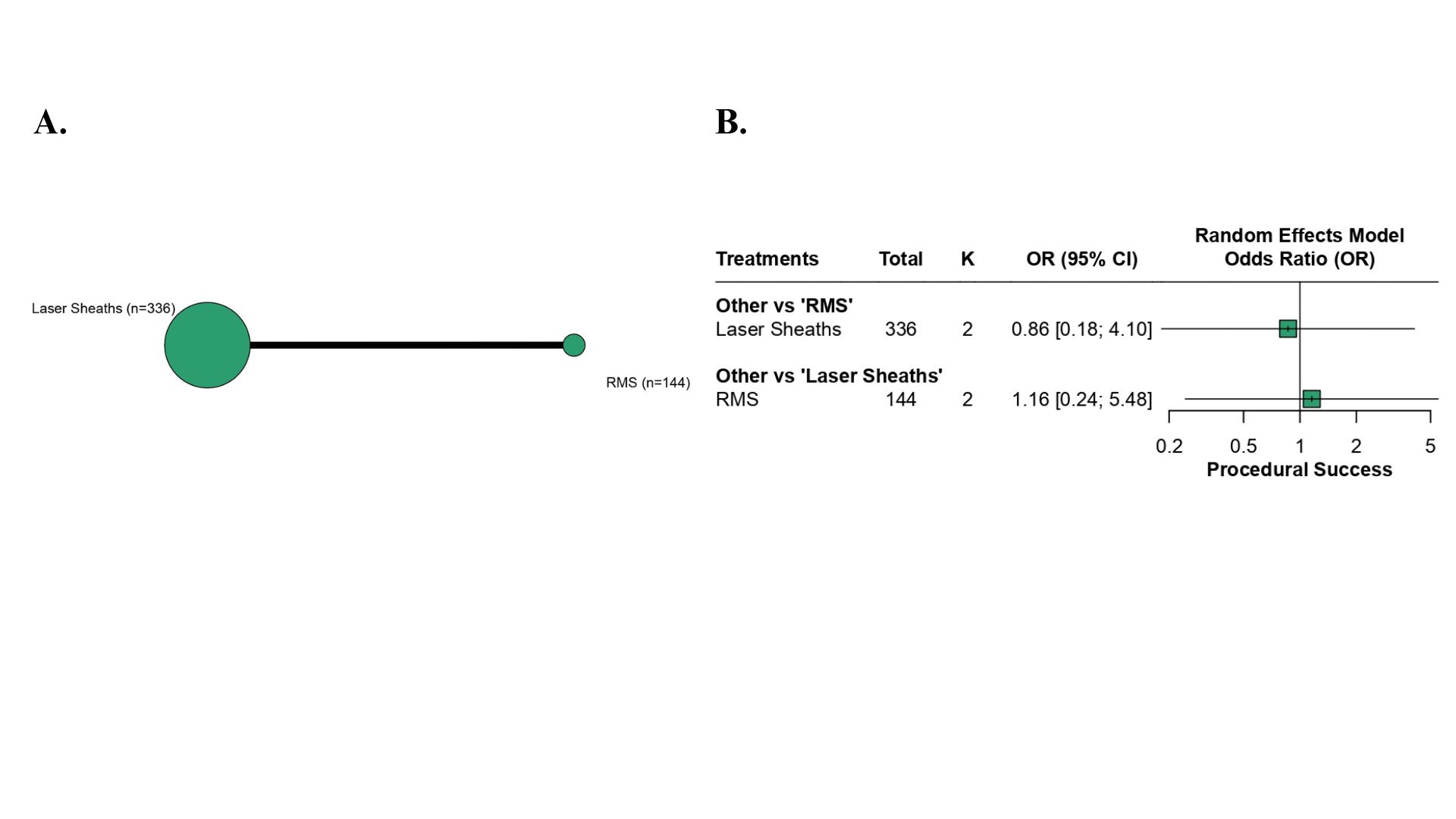


**Caption:** A. Network graph of the sensitivity analysis for risk of bias, assessing procedural success. B. Forest plot displaying effect sizes for procedural success, comparing competing treatment arms for risk of bias. K represents the number of studies providing direct evidence. **Abbreviations:** RMS, Rotating Mechanical Sheaths; OR, Odds Ratio.

## **Supplemental Results 16.** Exploring heterogeneity/inconsistency for primary endpoints

To assess heterogeneity and inconsistency within our network model, we first evaluated the I² statistics, which quantify the proportion of total variation attributable to between-study heterogeneity rather than chance. For Clinical Success, the overall heterogeneity was negligible (I² = 0%; Q = 1.19; p = 0.8803), suggesting minimal statistical inconsistency among the included studies. In contrast, the analysis of Procedural Success revealed substantial heterogeneity (I² = 60.3%; Q = 10.07; p = 0.0392), indicating significant variability in effect estimates across studies. For Major Complications, we observed moderate heterogeneity (I² = 29.5%; Q = 11.34; p = 0.1830), which did not reach statistical significance [Supplemental Results 17]. Subsequently, we decomposed heterogeneity into two distinct components: within-design heterogeneity and between-design inconsistency. Here, a design refers to a specific set of treatment comparisons evaluated within an individual study (e.g., Laser vs. RMS, or Laser vs. RMS vs. Femoral Approach). Within-design heterogeneity arises when true effect size differences exist among studies that examine identical treatment comparisons, reflecting variability in outcomes due to study-specific factors (e.g., patient populations, protocols). Between-design inconsistency, however, occurs when the relative treatment effects diverge across different study designs, potentially due to effect modification, interactions between treatments, or contextual differences in their application.^13–15^

For Clinical Success, the design-based decomposition of Cochran’s Q revealed non-significant between-study heterogeneity (Q = 1.09; p = 0.5797) and within-study inconsistency (Q = 0.10; p = 0.9531). In contrast, for Procedural Success, heterogeneity appeared to be more strongly associated with variability between study designs (Q = 5.48; p = 0.0646) than within designs (Q = 4.60; p = 0.1005), though neither reached statistical significance. Regarding major complications, a moderate degree of heterogeneity was observed, primarily driven by between-design variability (Q = 9.94; p = 0.0415) rather than within-design variations (Q = 1.38; p = 0.7104) [Supplemental Results 17]. To further investigate these findings, we computed total inconsistency using a full design-by-treatment interaction random-effects model with τ² estimated via the method of moments. In the analysis of Clinical Success, the design-by-treatment interaction model showed no evidence of inconsistency (Q = 1.09; p = 0.5797), with the Q statistic remaining identical to the original homogeneous network estimate, thereby confirming perfect consistency. For Procedural Success, the consistency assessment under this model yielded a non-significant Q statistic (Q = 1.78; p = 0.4109), representing a substantial reduction from the initial value of 10.07. This pronounced decrease indicates that between-design inconsistency was effectively resolved by the random-effects structure, suggesting that the model successfully accounted for the majority of effect size variability across studies. In contrast, the evaluation of major complications demonstrated more limited improvement, with the Q statistic decreasing only marginally from 11.34 to 9.94 (p = 0.0415). While the model partially explained the observed heterogeneity, the persistent residual variance implies the potential influence of unmeasured study-level covariates or additional sources of variation not captured by the current modeling framework [Supplemental Results 17].

To evaluate disagreement between direct and indirect comparisons, we employed two complementary approaches: First, we performed a net-split analysis to decompose network estimates into their direct and indirect evidence components, enabling assessment of inconsistency at the individual comparison level. Second, we quantified the relative contributions of direct versus indirect evidence for each comparison. This dual analytical framework additionally yields two key metrics: minimal parallelism and mean path length. Following the methodological guidance of König, Krahn, and Binder (2013), we note that a mean path length exceeding 2 suggests the corresponding comparison estimate requires cautious interpretation. While mixed estimates typically demonstrate superior precision compared to either direct or indirect evidence alone, we identified significant disagreement (indicating inconsistency) when the direct-indirect comparison test yielded P-values < 0.05, with both z-values and corresponding P-values reported for these assessments. Importantly, under random-effects modeling, result precision may be compromised in two distinct scenarios: when direct and indirect evidence demonstrate inconsistency, or when indirect evidence displays greater heterogeneity than its direct counterpart.^16,17^

For major complications, the test for disagreement between direct and indirect estimates showed no significant differences across all study designs [Supplemental Results 18]. Notably, the Laser Sheaths versus Traction comparison demonstrated a mean path length of 2, reflecting its derivation from extended indirect evidence pathways [Supplemental Results 19]. The net-split analysis revealed overall consistency between direct and indirect evidence in our network model, though the Laser Sheaths-Traction comparison warrants particular interpretive caution. For clinical success, the P-value for the test of disagreement between direct and indirect evidence indicated no statistically significant inconsistency across study designs [Supplemental Results 21]. When assessing the proportion of direct and indirect evidence, we observed a predominance of direct evidence in most comparisons. The mean path length was 2 for the comparison between laser sheaths and manual traction, which was based exclusively on indirect evidence [Supplemental Results 22]. For procedural success, the test for disagreement between direct and indirect evidence was nonsignificant for all study designs [Supplemental Results 24]. We observed a high proportion of direct evidence, with a mean path length of <2 across all comparisons [Supplemental Results 25].

To further assess local inconsistency and evaluate how study designs contribute to it, we generated a net heat plot, which visualizes hotspots of inconsistency between specific direct evidence across the entire network. This method produces a quadratic heatmap in which each design in a row is compared to other designs in the columns. Importantly, rows and columns represent specific study designs rather than individual treatment comparisons within the network. Consequently, the plot also includes rows and columns corresponding to the design used in multi-arm studies. Gray boxes indicate the relative importance of a treatment comparison in estimating another treatment comparison, with larger boxes signifying greater relevance. The colored backgrounds represent the level of inconsistency in the design of a given row that can be attributed to the design in the corresponding column. The color gradient ranges from deep red (indicating strong inconsistency) to blue (indicating that evidence from the given design supports the evidence in the row).^14,15,18^

For the net heat plot of major complications, we identified fields with an intense orange background in the upper left corner, indicating greater inconsistency within the network, particularly in the comparison of Femoral Approach vs. Laser Sheaths and the multi-arm design Femoral Approach vs. Laser Sheaths vs. RMS [Supplemental Results 20]. In the net heat plot of clinical success, we observed no fields with a dark red background, suggesting no substantial inconsistency. The yellow-highlighted areas in the lower right section indicate that these designs contribute moderately to the network’s inconsistency. The large boxes along the main diagonal demonstrate that direct comparisons had a strong influence within the network [Supplemental Results 23]. For the net heat plot of procedural success, we detected some degree of local inconsistency in the lower right corner, primarily in the RMS vs. Laser Sheaths comparison and the multi-arm design Femoral Approach vs. Laser Sheaths vs. RMS, represented by a dark yellow background. No fields with a dark red background were observed [Supplemental Results 26]. The observed inconsistencies in the net heat plots may stem from multiple confounding factors, including heterogeneity in study designs, variability in outcome definitions, differences in operator experience, lead-specific characteristics (e.g., implant duration, extent of fibrosis, or lead type), and procedural complexity (e.g., presence of multiple leads, severe adhesions, or prior extraction attempts).

## **Supplemental Results 17.** Heterogeneity/Inconsistency and Design-Based Decomposition of Cochran’s Q

| **Heterogeneity/Inconsistency** | **Clinical Success** | **Procedural Success** | **Major Complications** |
| --- | --- | --- | --- |
| **Overall Heterogeneity** *I² (Q; p-value)* | 0% (Q = 1.19; p = 0.8803) | 60.3% (Q = 10.07; p = 0.0392) | 29.5% (Q = 11.34; p = 0.1830) |
| **Between-study Heterogeneity** *Q; p-value* | Q = 1.09; p = 0.5797 | Q = 5.48; p = 0.0646 | Q = 9.94; p = 0.0415 |
| **Within-study Heterogeneity** *Q; p-value* | Q = 0.10; p = 0.9531 | Q = 4.60; p = 0.1005 | Q = 1.38; p = 0.7104 |
| **Full design-by-treatment interaction  random effects model** *Q; p-value* | Q = 1.09; p = 0.5797 | Q = 1.78; p = 0.4109 | Q = 9.94; p = 0.0415 |

**Caption:** Metrics of heterogeneity and inconsistency for primary endpoints in the network model. Overall heterogeneity is expressed as I², along with corresponding Q statistics and p-values. Within-design heterogeneity (differences in effect sizes within studies using the same treatment set) and between-design inconsistency (differences across treatment designs) are provided with their respective Q statistics and p-values. The Q statistics are also presented to assess consistency under the assumption of a full design-by-treatment interaction random effects model.

## **Supplemental Results 18.** Net-Split Plot of Direct vs. Indirect Evidence for Major Complications


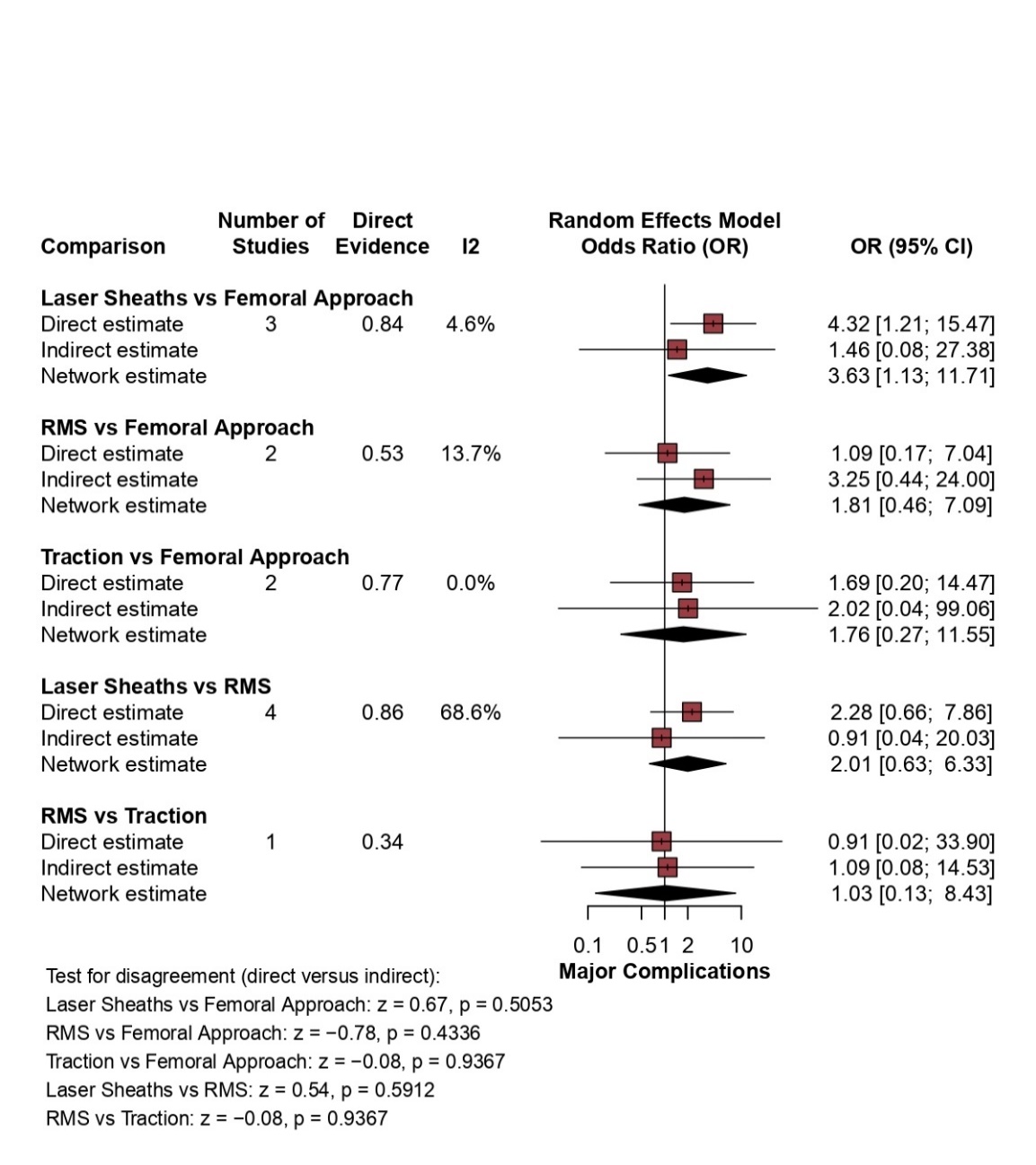


**Caption:** Forest plot of net split results for direct and indirect evidence, as well as network estimates for major complications. This method separates our network estimates into contributions from direct and indirect evidence, allowing us to assess and control for inconsistency in specific comparisons within the network. A p-value > 0.05 was considered to indicate good consistency, whereas a p-value < 0.05 indicated inconsistency. **Abbreviations:** RMS, Rotating Mechanical Sheaths; OR, odds ratio; CI, confidence interval.

## **Supplemental Results 19.** Proportion of Direct and Indirect Evidence for Major Complications


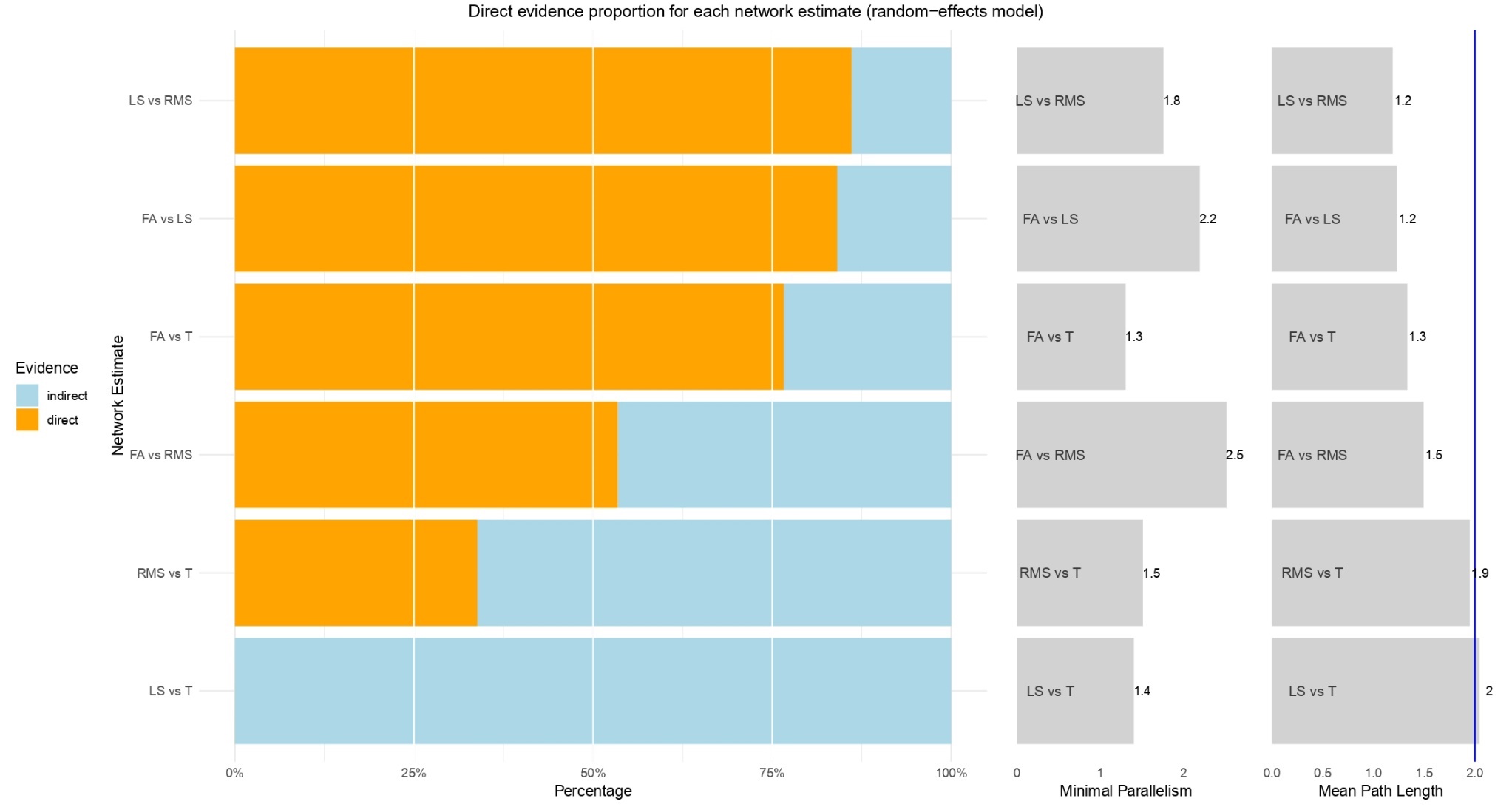


**Caption:** Plot illustrating and quantifying the direct evidence proportion, mean path length, and aggregated minimal parallelism in the frequentist network model. A mean path length > 2 indicates that comparison estimates should be interpreted with caution. **Abbreviations:** FA, Femoral Approach; LS, Laser Sheaths; RMS, Rotating Mechanical Sheaths; T, Traction.

## **Supplemental Results 20.** Net Heat Plot for Inconsistency Assessment in Network Meta-Analysis of Major Complications


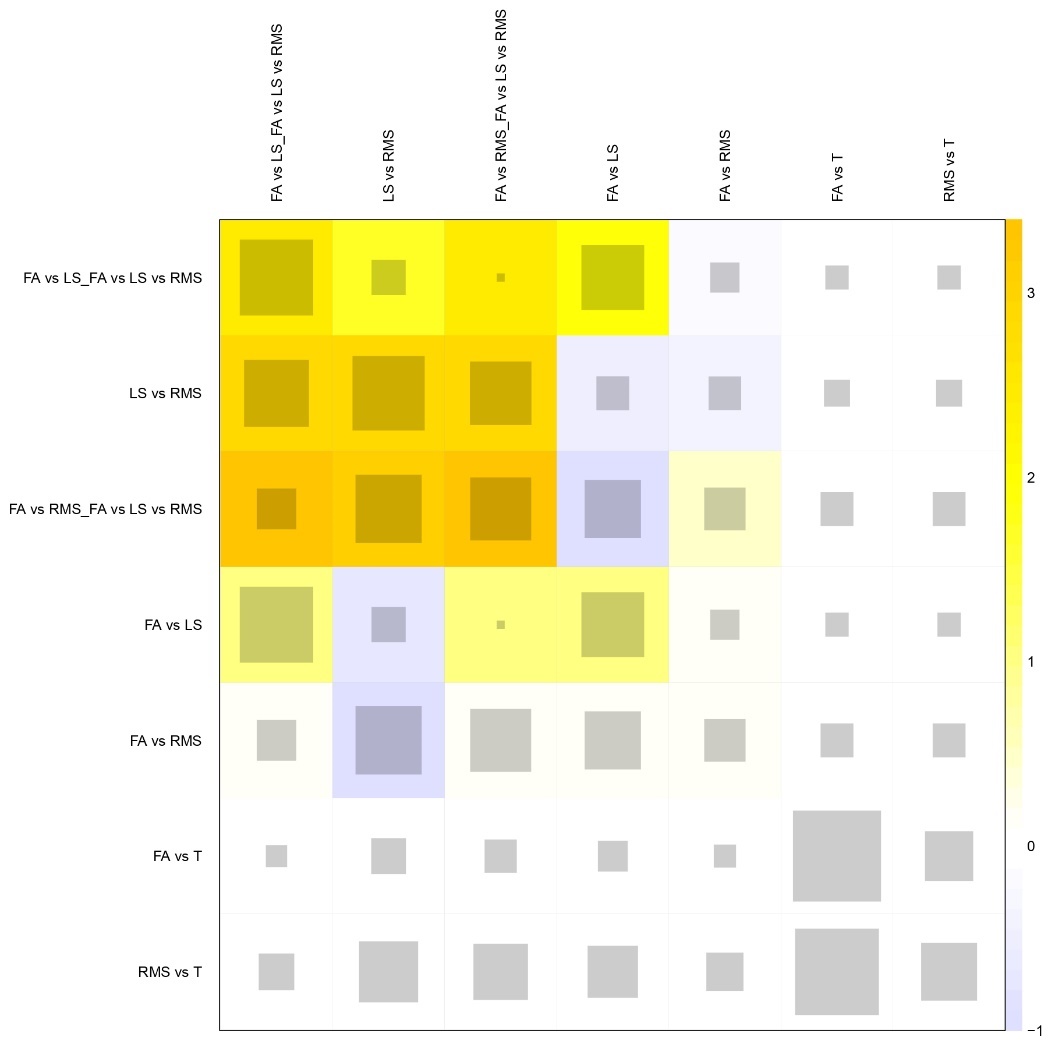


**Caption:** Heat plot visualizing the inconsistency across treatment designs in a network meta-analysis. Each cell represents the relationship between a row design and a column design, with gray boxes indicating the importance of each treatment comparison in the overall network estimate. The colored backgrounds reflect the level of inconsistency, with cooler colors (blue) supporting the network evidence and warmer colors (red) highlighting areas of disagreement. **Abbreviations:** FA, Femoral Approach; LS, Laser Sheaths; RMS, Rotating Mechanical Sheaths; T, Traction.

## **Supplemental Results 21.** Net-Split Plot of Direct vs. Indirect Evidence for Clinical Success


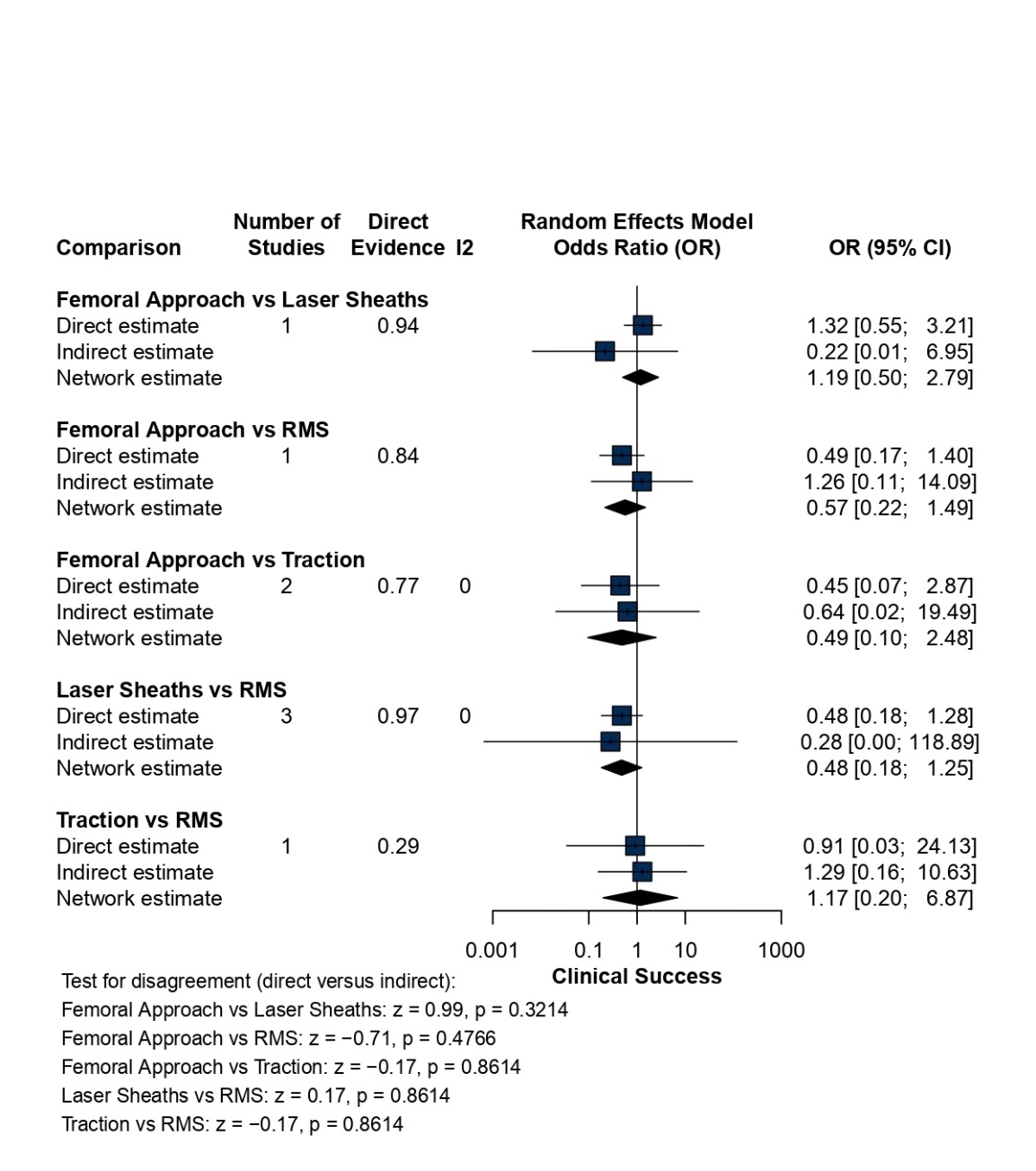


**Caption:** Forest plot of net split results for direct and indirect evidence, as well as network estimates for clinical success. This method separates our network estimates into contributions from direct and indirect evidence, allowing us to assess and control for inconsistency in specific comparisons within the network. A p-value > 0.05 was considered to indicate good consistency, whereas a p-value < 0.05 indicated inconsistency. **Abbreviations:** RMS, Rotating Mechanical Sheaths; OR, odds ratio; CI, confidence interval.

## **Supplemental Results 22.** Proportion of Direct and Indirect Evidence for Clinical Success


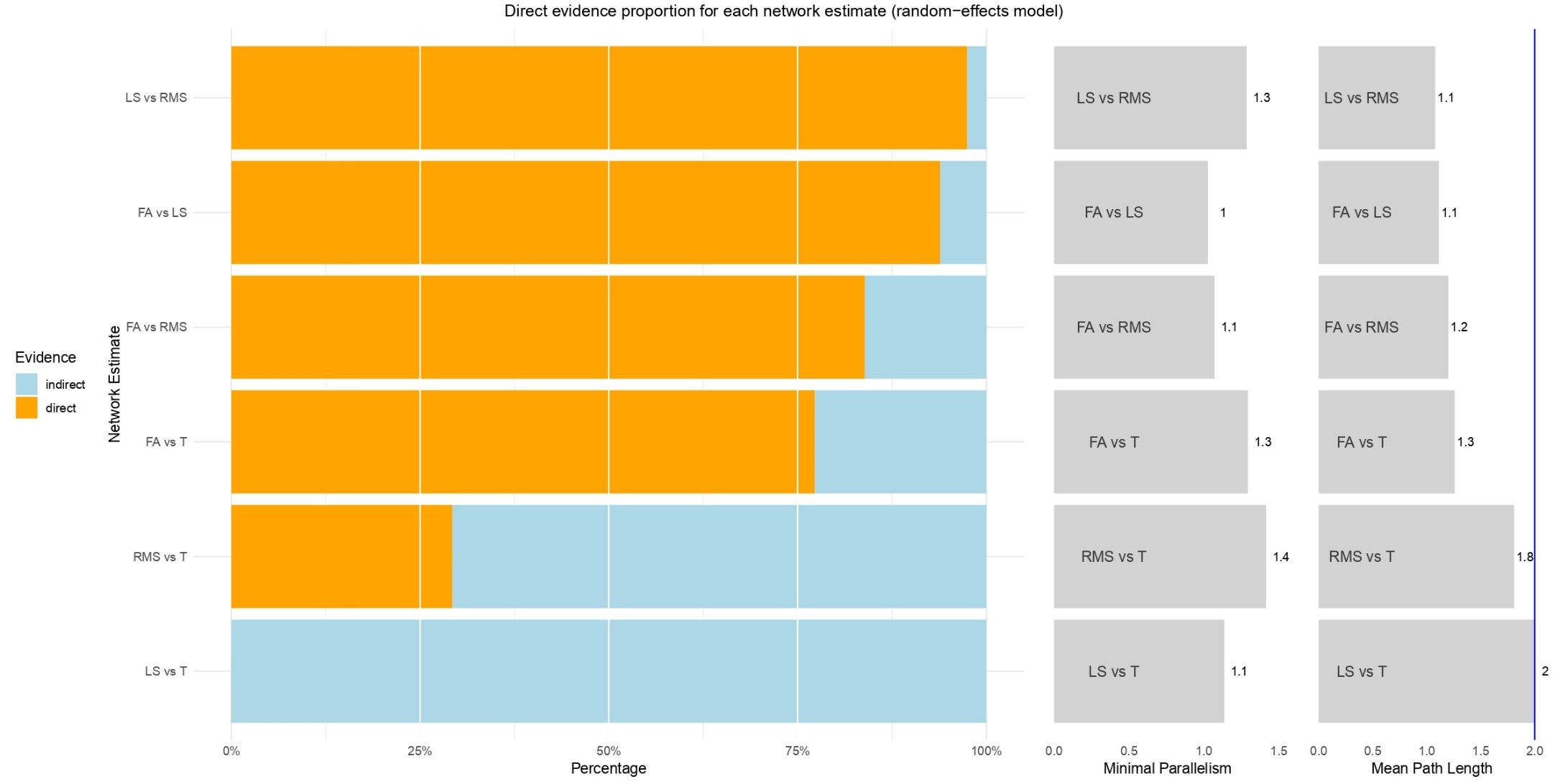


**Caption:** Plot illustrating and quantifying the direct evidence proportion, mean path length, and aggregated minimal parallelism in the frequentist network model. A mean path length > 2 indicates that comparison estimates should be interpreted with caution. **Abbreviations:** FA, Femoral Approach; LS, Laser Sheaths; RMS, Rotating Mechanical Sheaths; T, Traction.

## **Supplemental Results 23.** Net Heat Plot for Inconsistency Assessment in Network Meta-Analysis of Clinical Success


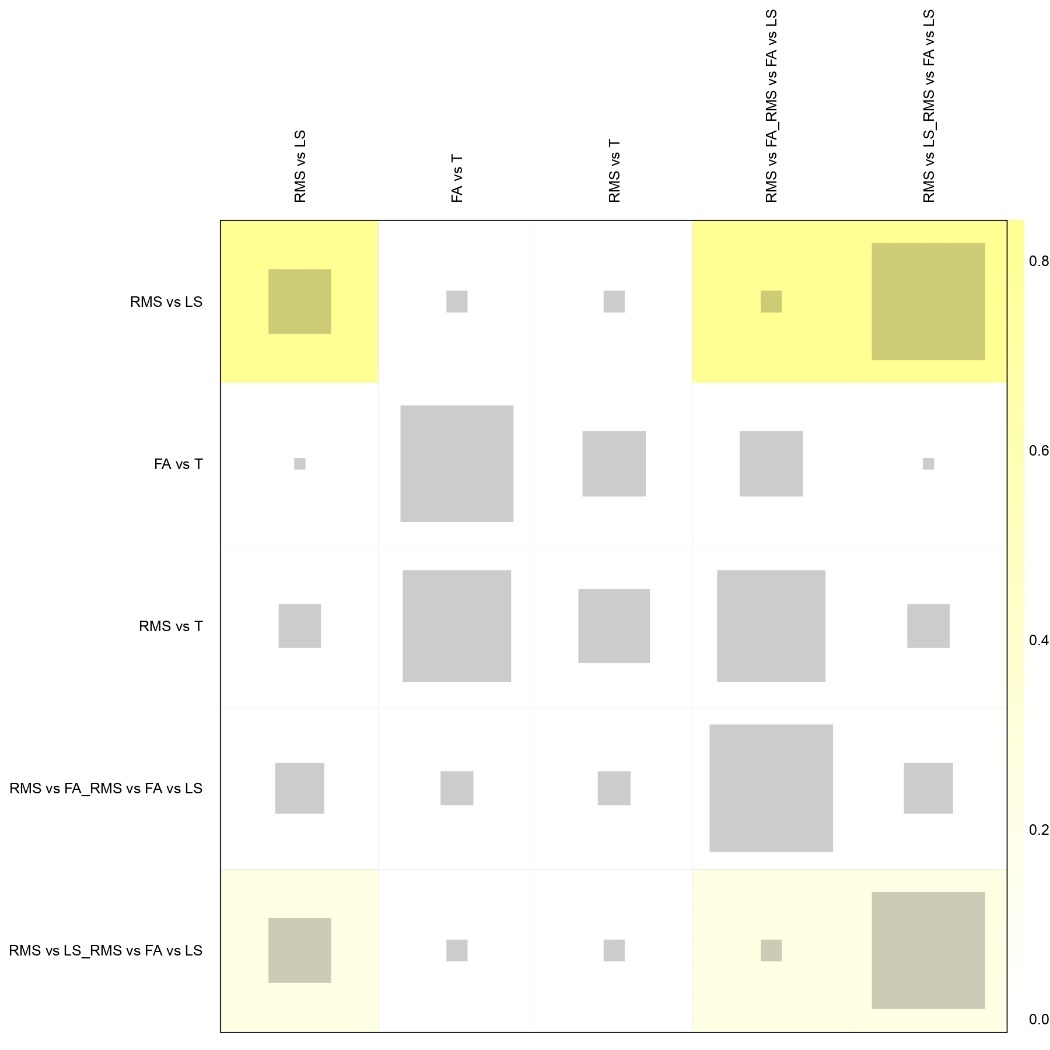


**Caption:** Heat plot visualizing the inconsistency across treatment designs in a network meta-analysis. Each cell represents the relationship between a row design and a column design, with gray boxes indicating the importance of each treatment comparison in the overall network estimate. The colored backgrounds reflect the level of inconsistency, with cooler colors (blue) supporting the network evidence and warmer colors (red) highlighting areas of disagreement. **Abbreviations:** FA, Femoral Approach; LS, Laser Sheaths; RMS, Rotating Mechanical Sheaths; T, Traction.

## **Supplemental Results 24.** Net-Split Plot of Direct vs. Indirect Evidence for Procedural Success


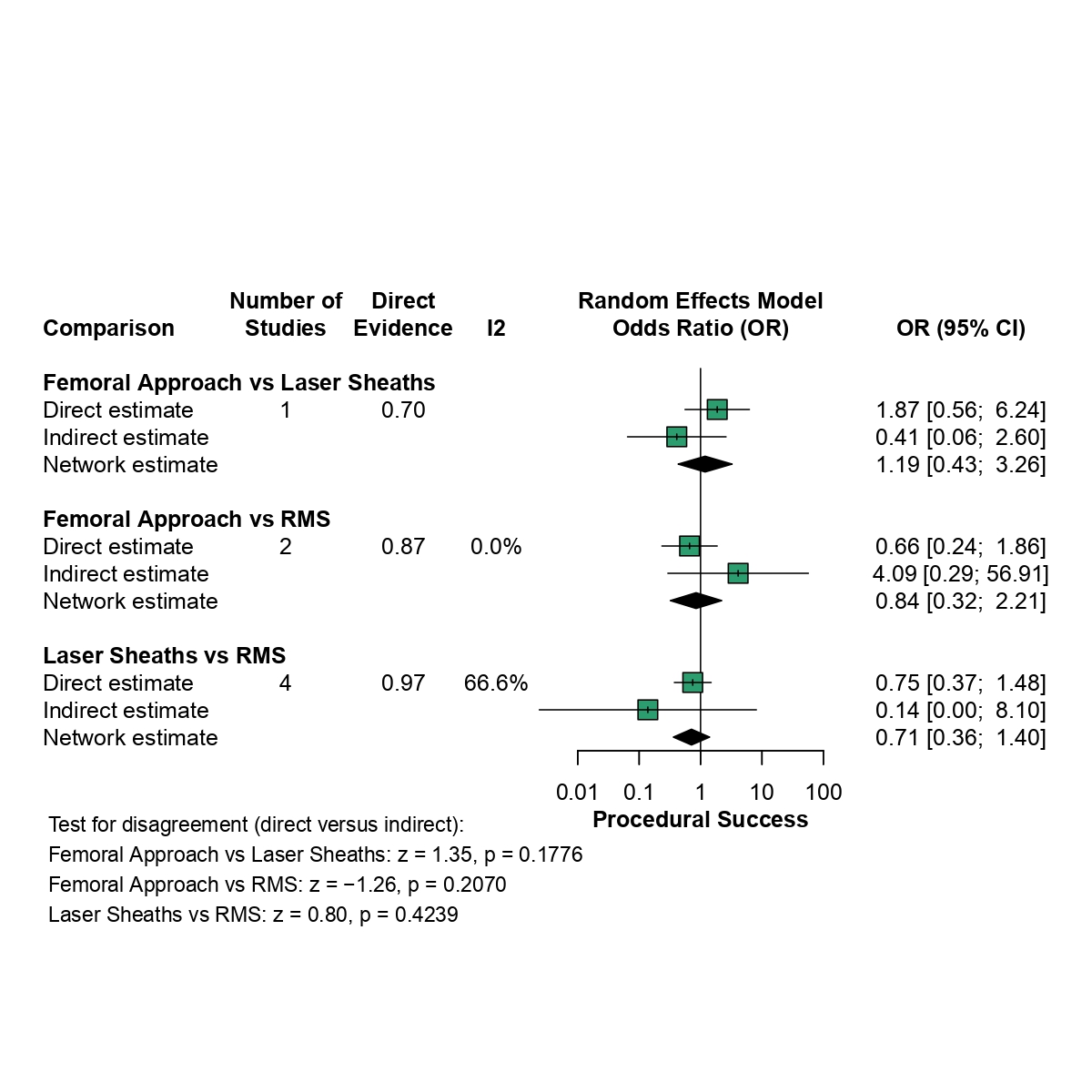


**Caption:** Forest plot of net split results for direct and indirect evidence, as well as network estimates for procedural success. This method separates our network estimates into contributions from direct and indirect evidence, allowing us to assess and control for inconsistency in specific comparisons within the network. A p-value > 0.05 was considered to indicate good consistency, whereas a p-value < 0.05 indicated inconsistency. **Abbreviations:** RMS, Rotating Mechanical Sheaths; OR, odds ratio; CI, confidence interval.

## **Supplemental Results 25.** Proportion of Direct and Indirect Evidence for Procedural Success


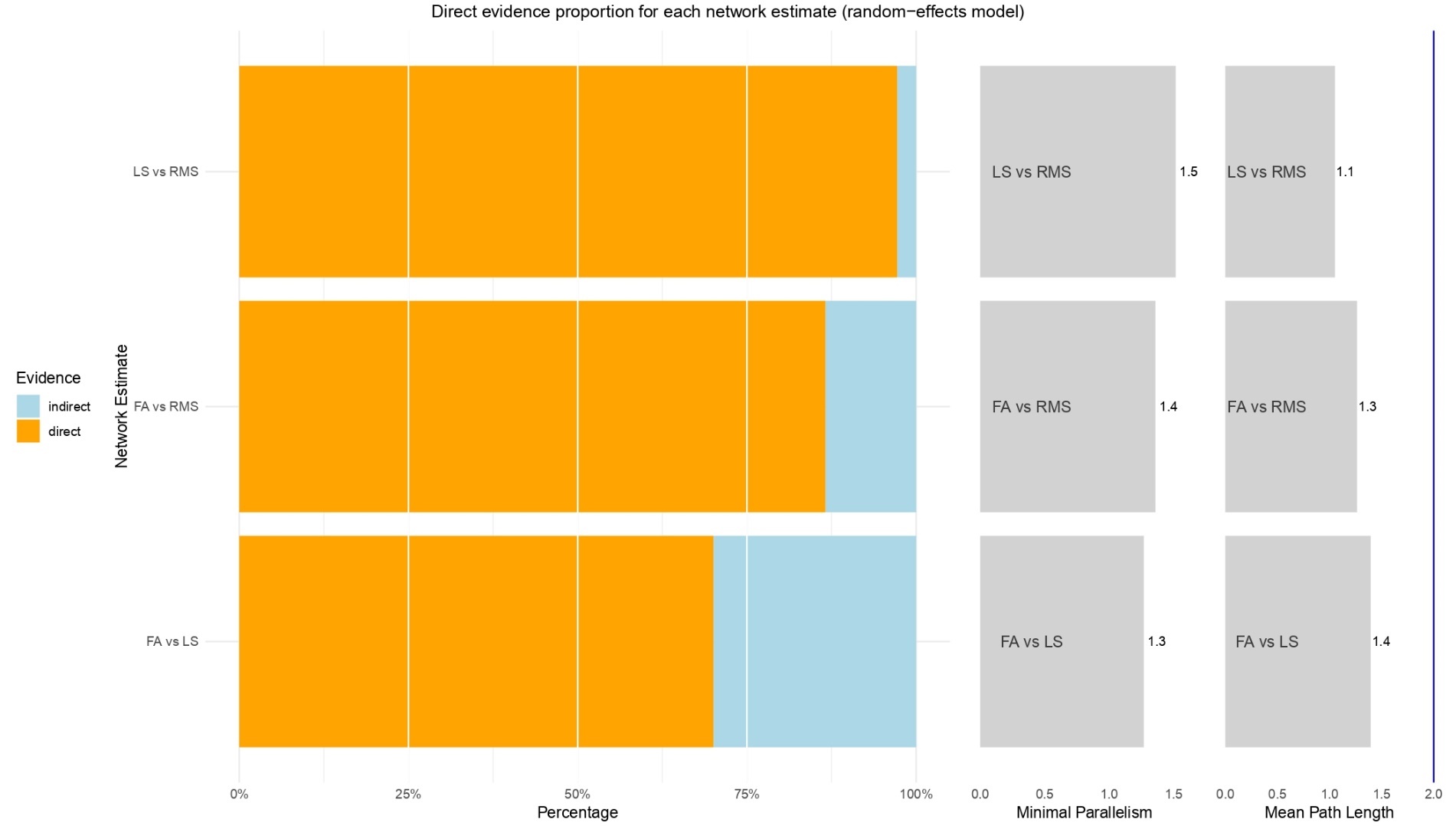


**Caption:** Plot illustrating and quantifying the direct evidence proportion, mean path length, and aggregated minimal parallelism in the frequentist network model. A mean path length > 2 indicates that comparison estimates should be interpreted with caution. **Abbreviations:** FA, Femoral Approach; LS, Laser Sheaths; RMS, Rotating Mechanical Sheaths; T, Traction.

## **Supplemental Results 26.** Net Heat Plot for Inconsistency Assessment in Network Meta-Analysis of Procedural Success


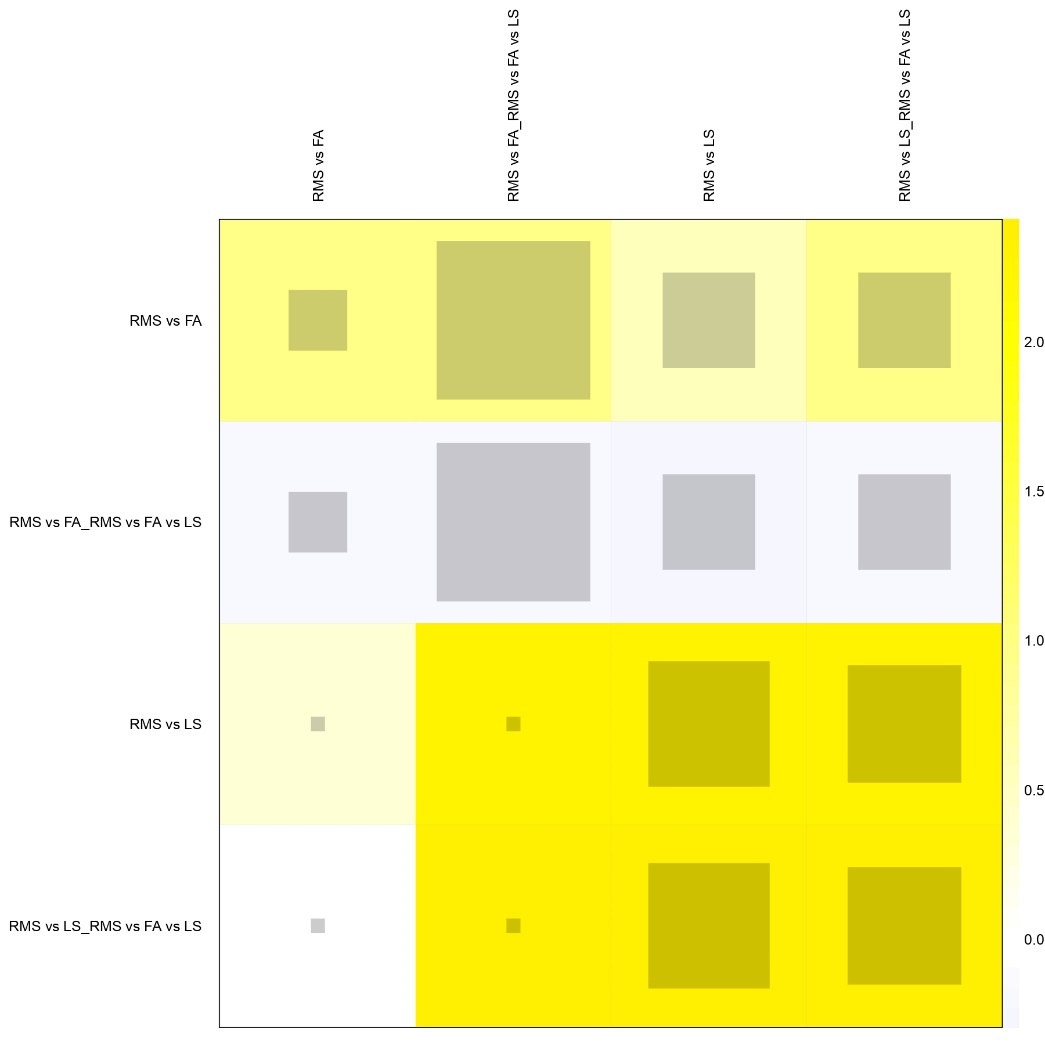


**Caption:** Heat plot visualizing the inconsistency across treatment designs in a network meta-analysis. Each cell represents the relationship between a row design and a column design, with gray boxes indicating the importance of each treatment comparison in the overall network estimate. The colored backgrounds reflect the level of inconsistency, with cooler colors (blue) supporting the network evidence and warmer colors (red) highlighting areas of disagreement. **Abbreviations:** FA, Femoral Approach; LS, Laser Sheaths; RMS, Rotating Mechanical Sheaths; T, Traction.

## **Supplemental Results 27.** Risk of Bias Assessment

## **Supplemental Results 27A.** ROBINS-I Traffic Light Plot for Bias Assessment


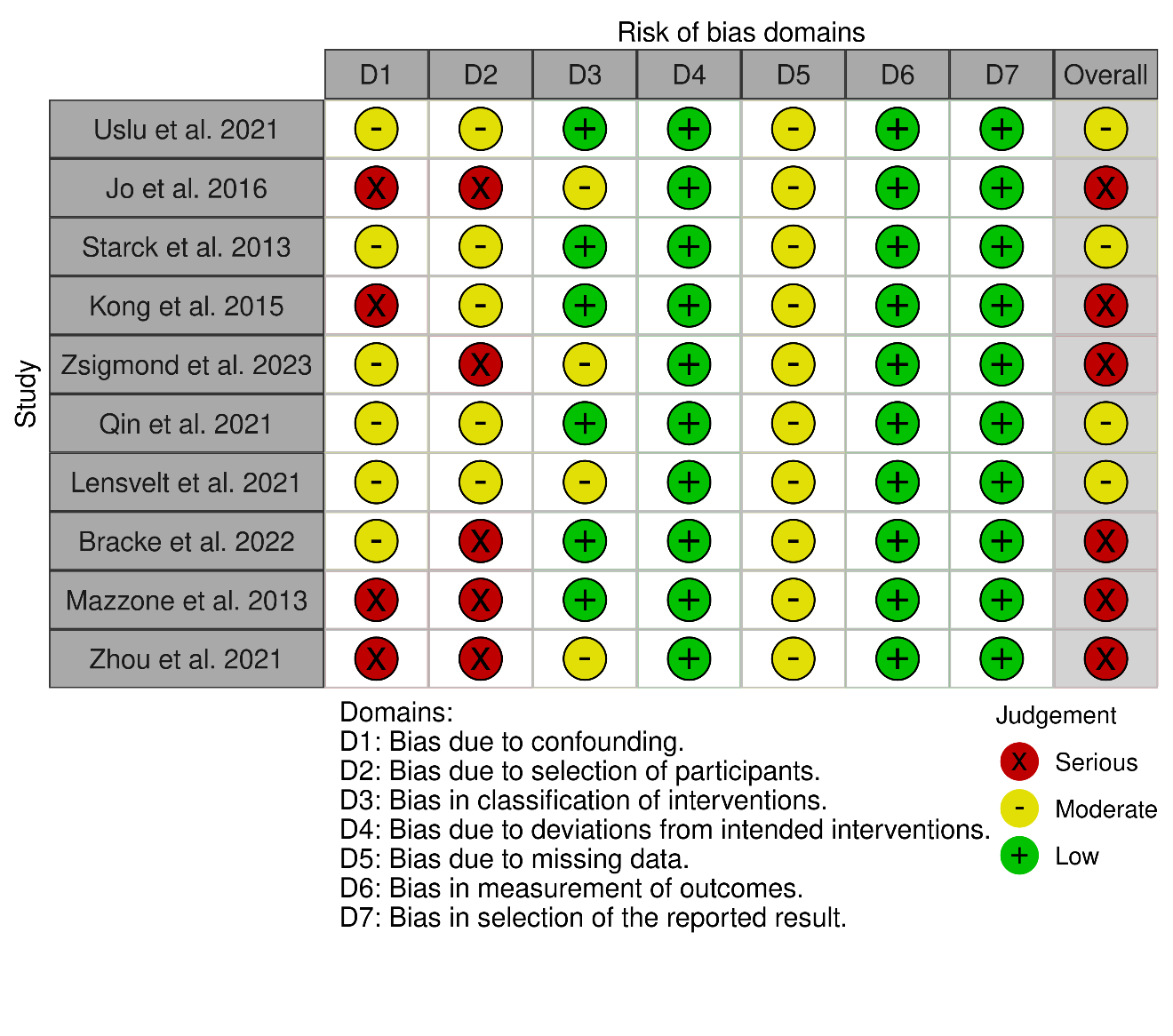


## **Supplemental Results 27B.** ROBINS-I Summary Bar Plot for Bias Assessment


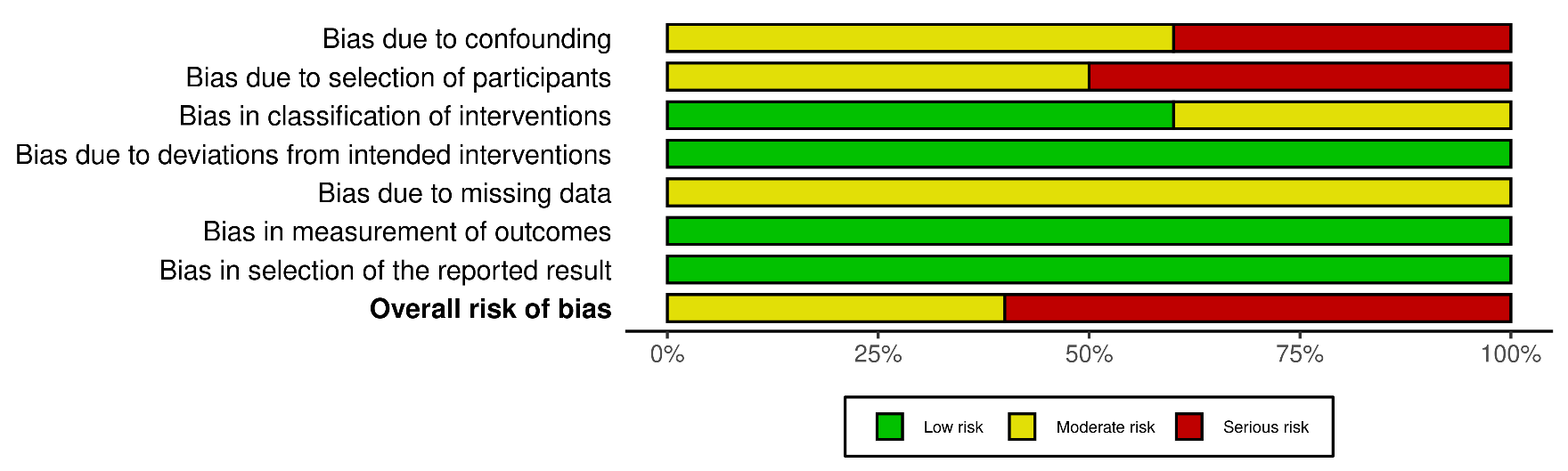


## **Supplemental Results 27C.** RoB 2 Traffic Light Plot for Bias Assessment


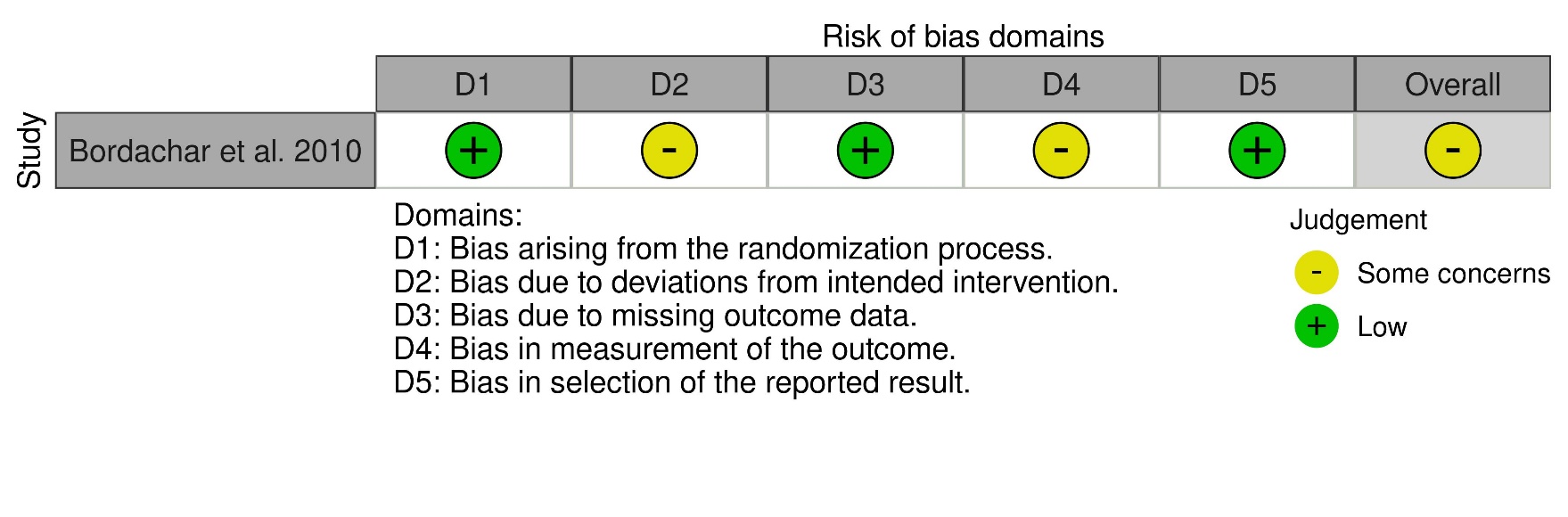


## **Supplemental Results 28.** Publication Bias Assessment

## **Supplemental Results 28A.** Comparison-Adjusted Funnel Plot for Major Complications


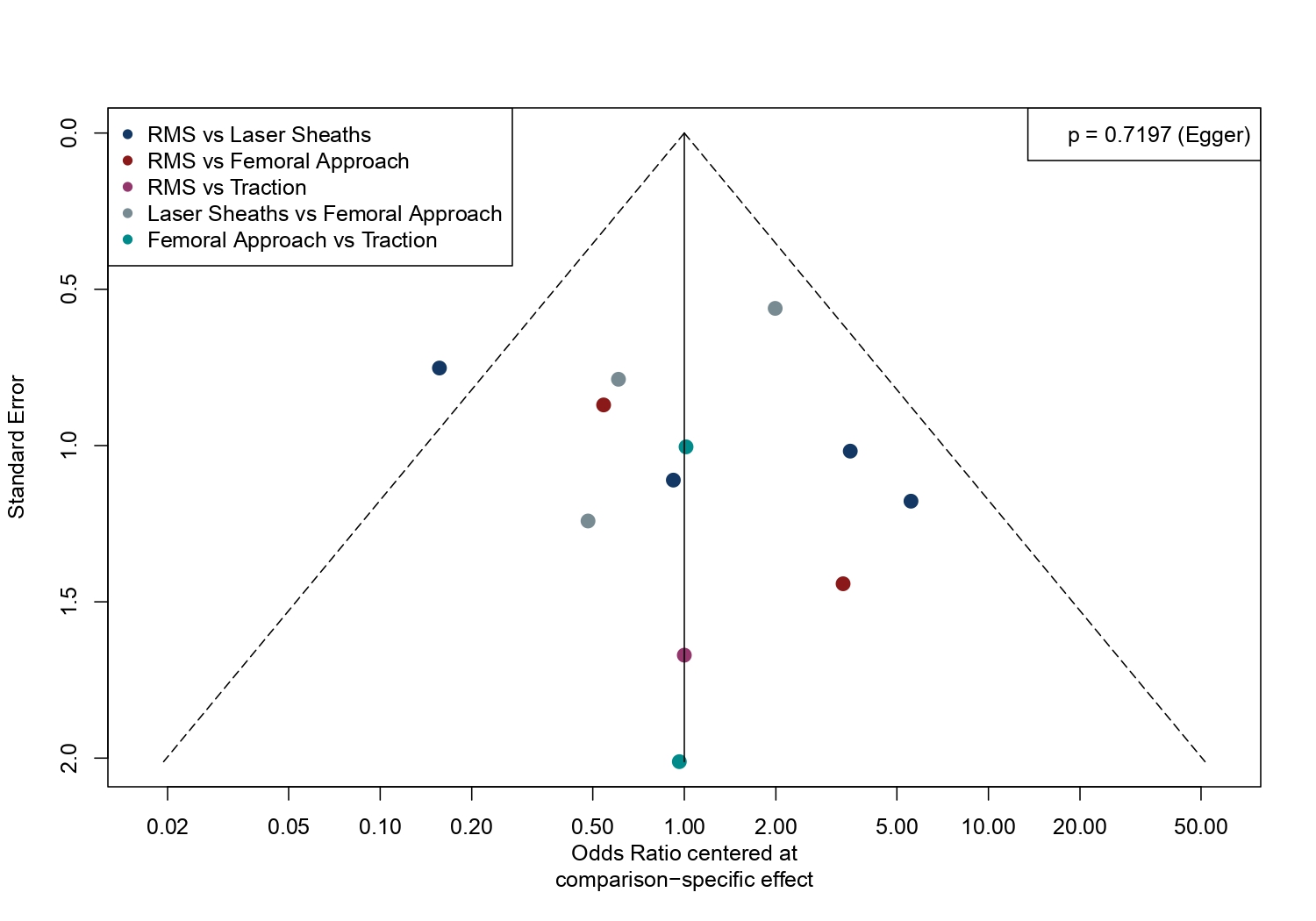


**Caption:** Comparison-adjusted funnel plot for the major complications network, representing the same pairwise direct comparisons as in the original study. This scatter plot shows the effect size of each study plotted against a measure of its precision. The black vertical line represents the null hypothesis, indicating no difference between independent effect size estimates and comparison-specific pooled estimates. Egger’s test for asymmetry was not significant (p = 0.7197). **Abbreviations:** RMS, Rotating Mechanical Sheaths.

## **Supplemental Results 28B.** Comparison-Adjusted Funnel Plot for Clinical Success


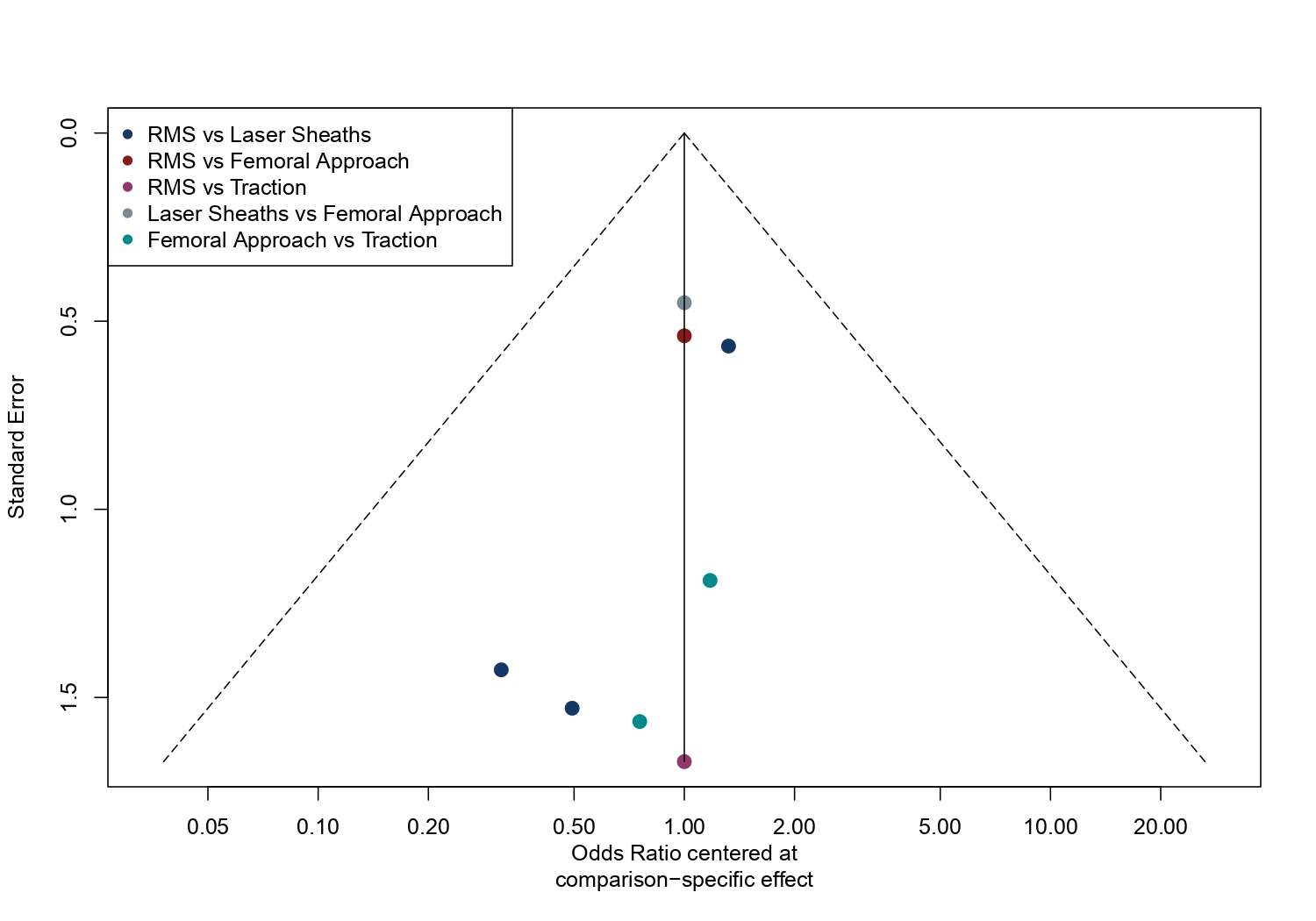


**Caption:** Comparison-adjusted funnel plot for the clinical success network, representing the same pairwise direct comparisons as in the original study. This scatter plot shows the effect size of each study plotted against a measure of its precision. The black vertical line represents the null hypothesis, indicating no difference between independent effect size estimates and comparison-specific pooled estimates. Egger’s test for asymmetry was not performed due to the limited number of studies (k = 6). **Abbreviations:** RMS, Rotating Mechanical Sheaths.

## **Supplemental Results 28C.** Comparison-Adjusted Funnel Plot for Procedural Success


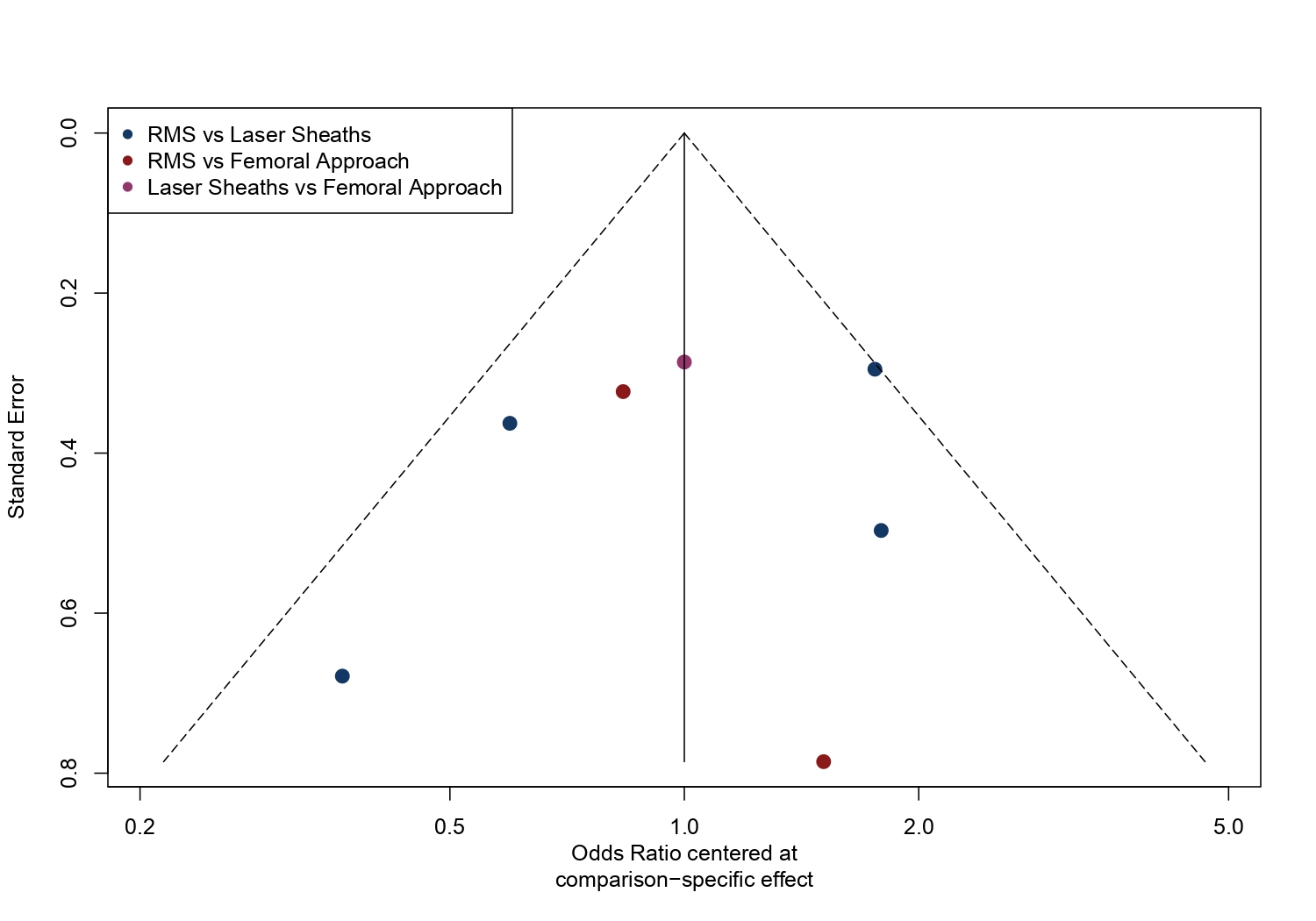


**Caption:** Comparison-adjusted funnel plot for the procedural success network, representing the same pairwise direct comparisons as in the original study. This scatter plot shows the effect size of each study plotted against a measure of its precision. The black vertical line represents the null hypothesis, indicating no difference between independent effect size estimates and comparison-specific pooled estimates. Egger’s test for asymmetry was not performed due to the limited number of studies (k = 5). **Abbreviations:** RMS, rotating mechanical sheaths.

## **Supplemental References**

1. Uslu A, Küp A, Kanar BG, et al. Transvenous extraction of pacemaker leads via femoral approach using a gooseneck snare. *Herz*. 2021;46(1):82-88. doi:10.1007/s00059-020-04987-z

2. Jo U, Kim J, Hwang YM, et al. Transvenous Lead Extraction via the Inferior Approach Using a Gooseneck Snare versus Simple Manual Traction. *Korean Circ J*. 2016;46(2):186. doi:10.4070/kcj.2016.46.2.186

3. Starck CT, Rodriguez H, Hurlimann D, et al. Transvenous lead extractions: comparison of laser vs. mechanical approach. *Europace*. 2013;15(11):1636-1641. doi:10.1093/europace/eut086

4. Bordachar P, Defaye P, Peyrouse E, et al. Extraction of Old Pacemaker or Cardioverter-Defibrillator Leads by Laser Sheath Versus Femoral Approach. *Circ Arrhythm Electrophysiol*. 2010;3(4):319-323. doi:10.1161/CIRCEP.109.933051

5. Kong J, Tian Y, Guo F, et al. Snare sheath versus evolution sheath in transvenous lead extraction. *Int J Clin Exp Med*. 2015;8(11):21975-21980.

6. Zsigmond EJ, Saghy L, Benak A, et al. A head-to-head comparison of laser vs. powered mechanical sheaths as first choice and second line extraction tools. *EP Europace*. 2023;25(2):591-599. doi:10.1093/europace/euac200

7. Misra S, Swayampakala K, Coons P, et al. Outcomes of transvenous lead extraction using the TightRailTM mechanical rotating dilator sheath and excimer laser sheath. *J Cardiovasc Electrophysiol*. 2021;32(7):1969-1978. doi:10.1111/jce.15105

8. Qin D, Chokshi M, Sabeh MK, et al. Comparison between TightRail rotating dilator sheath and GlideLight laser sheath for transvenous lead extraction. *Pacing and Clinical Electrophysiology*. 2021;44(5):895-902. doi:10.1111/pace.14206

9. Lensvelt LMH, Egorova AD, Schalij MJ, et al. Mechanical extraction of cardiac implantable electronic devices leads with long dwell time: Efficacy and safety of the step up approach. *Pacing and Clinical Electrophysiology*. 2021;44(1):120-128. doi:10.1111/pace.14094

10. Bracke FA, Rademakers N, Verberkmoes N, Van ’t Veer M, van Gelder BM. Comparison between laser sheaths, femoral approach and rotating mechanical sheaths for lead extraction. *Netherlands Heart Journal*. 2022;30(5):267-272. doi:10.1007/s12471-021-01652-w

11. Mazzone P, Tsiachris D, Marzi A, et al. Advanced techniques for chronic lead extraction: heading from the laser towards the evolution system. *Europace*. 2013;15(12):1771-1776. doi:10.1093/europace/eut126

12. Zhou X, Ze F, Li X, Wang B. Efficacy and safety of transvenous lead extraction in the Chinese octogenarian patients. *Clin Cardiol*. 2021;44(7):971-977. doi:10.1002/clc.23629

13. Jackson D, Barrett JK, Rice S, White IR, Higgins JPT. A design‐by‐treatment interaction model for network meta‐analysis with random inconsistency effects. *Stat Med*. 2014;33(21):3639-3654. doi:10.1002/sim.6188

14. Jackson D, White IR, Riley RD. Quantifying the impact of between‐study heterogeneity in multivariate meta‐analyses. *Stat Med*. 2012;31(29):3805-3820. doi:10.1002/sim.5453

15. Higgins JPT, Jackson D, Barrett JK, Lu G, Ades AE, White IR. Consistency and inconsistency in network meta‐analysis: concepts and models for multi‐arm studies. *Res Synth Methods*. 2012;3(2):98-110. doi:10.1002/jrsm.1044

16. Dias S, Welton NJ, Caldwell DM, Ades AE. Checking consistency in mixed treatment comparison meta‐analysis. *Stat Med*. 2010;29(7-8):932-944. doi:10.1002/sim.3767

17. König J, Krahn U, Binder H. Visualizing the flow of evidence in network meta‐analysis and characterizing mixed treatment comparisons. *Stat Med*. 2013;32(30):5414-5429. doi:10.1002/sim.6001

18. Krahn U, Binder H, König J. A graphical tool for locating inconsistency in network meta-analyses. *BMC Med Res Methodol*. 2013;13(1):35. doi:10.1186/1471-2288-13-35
